# Safety of budesonide/glycopyrronium/formoterol fumarate dihydrate delivered by HFO-1234ze versus HFA-134a in chronic obstructive pulmonary disease: a phase 3, multi-site, randomised, double-blind, parallel-group, active-comparator study

**DOI:** 10.1016/j.eclinm.2025.103402

**Published:** 2025-08-12

**Authors:** Omar S. Usmani, Fernando J. Martinez, Hitesh Pandya, Matthew Camiolo, Artur Bednarczyk, Kinga Kucz, Marek Kokot, Christer Gottfridsson, Magnus Aurivillius, Lars Pettersson, Jie Mei, Karin Skansen, Jennifer L. Bell, David Petullo, Kathryn Collison, Patrik Bondarov, Mandeep Jassal, Mehul Patel

**Affiliations:** a Imperial College London and Royal Brompton Hospital, London, UK; b Division of Pulmonary, Allergy, and Critical Care Medicine, University of Massachusetts, Chan Medical School, Worcester, MA, USA; c Respiratory and Immunology, Clinical Development, BioPharmaceuticals R&D, AstraZeneca, Cambridge, UK; d Respiratory and Immunology, Clinical Development, BioPharmaceuticals R&D, AstraZeneca, Boston, MA, USA; e Respiratory and Immunology, Clinical Development, BioPharmaceuticals R&D, AstraZeneca Pharma Poland, Warsaw, Poland; f Cardiovascular Safety Centre of Excellence, Global Patient Safety, Chief Medical Office, Oncology R&D, AstraZeneca, Gothenburg, Sweden; g Global Patient Safety BioPharmaceuticals, Chief Medical Office, R&D, AstraZeneca, Gothenburg, Sweden; h Biostatistics, Phastar UK, London, UK; i Respiratory and Immunology, Biometrics, BioPharmaceuticals R&D, AstraZeneca, Gaithersburg, MD, USA; j Respiratory and Immunology, Clinical Operations, BioPharmaceuticals R&D, AstraZeneca, Durham, NC, USA; k Respiratory and Immunology, Clinical Operations, BioPharmaceuticals R&D, AstraZeneca, Gothenburg, Sweden; l Respiratory and Immunology, Clinical Development, BioPharmaceuticals R&D, AstraZeneca, Gaithersburg, MD, USA

**Keywords:** Budesonide/glycopyrronium/formoterol fumarate dihydrate (BGF), Chronic obstructive pulmonary disease (COPD), Inhaled triple therapy, Safety, Hydrofluoroalkane-134a (HFA-134a), Hydrofluoroolefin-1234ze (HFO-1234ze)

## Abstract

**Background:**

Pressurised metered dose inhalers (pMDIs) contain a hydrofluorocarbon propellant, such as hydrofluoroalkane-134a (HFA-134a), which is known to have global warming potential (GWP). Transitioning pMDIs to propellants with lower GWP will reduce the environmental impact of pMDIs. This study assessed the safety of a near-zero GWP propellant, hydrofluoroolefin-1234ze (HFO-1234ze), compared with HFA-134a when used in the delivery of budesonide/glycopyrronium/formoterol fumarate dihydrate (BGF) in participants with chronic obstructive pulmonary disease (COPD). The results of this study advance our understanding of the safety of HFO-1234ze compared with HFA-134a.

**Methods:**

This phase 3, double-blind, parallel-group study (ClinicalTrials.govNCT05573464) across 9 countries (Argentina, Bulgaria, Canada, Germany, Mexico, Poland, Turkey, the United Kingdom, the United States) included participants (aged 40–80 years) with physician-diagnosed COPD using dual or triple inhaled maintenance therapies, COPD Assessment Test score ≥10, ≥10 pack-years smoking history, and no comorbid diagnosis of asthma or other clinically significant diseases impacting study outcomes. Participants were randomised (1:1) to receive either BGF HFO-1234ze or BGF HFA-134a (two inhalations of 160/7·2/5·0 μg twice daily) for 12 weeks in the main safety analysis set (or 52 weeks [first 120 participants per treatment]). Safety endpoints included the incidence of adverse events (AEs), measures of vital signs, clinical laboratory tests, and electrocardiograms.

**Findings:**

Participants were recruited between 27 September 2022 and 19 May 2023. A total of 874 participants were screened. Of 558 treated participants (mean [standard deviation] age, 67·0 [7·4] years; male, 315 [56·5%]) in the 12-week safety analysis set, 280 received BGF HFO-1234ze, and 278 received BGF HFA-134a. The AE incidence was balanced between formulations in the 12-week (HFO-1234ze, 124 [44·3%]; HFA-134a, 114 [41·0%]) and 52-week (HFO-1234ze, 80 [66·7%]; HFA-134a, 94 [78·3%]) safety analysis sets.

**Interpretation:**

These findings support the potential for HFO-1234ze to replace HFA-134a in pMDIs containing BGF, which could be evaluated further in a real-world setting.

**Funding:**

The study was supported by 10.13039/100004325AstraZeneca.

**Research in context**

**Evidence before this study**

Like most other propellants used in pressurised metered dose inhalers (pMDIs), hydrofluoroalkane-134a (HFA-134a) is a hydrofluorocarbon known to have global warming potential (GWP). As such, next generation propellants with lower GWP are needed to reduce the environmental impact of pMDIs whilst safeguarding access to essential medicines. Hydrofluoroolefin-1234ze (HFO-1234ze) is a near-zero GWP propellant. Ahead of our study, a search was conducted within ClinicalTrials.gov to identify previous studies pertaining to the following areas: a) GWP of the HFA-134a versus HFO-1234ze propellant; and b) key safety considerations for the HFO-1234ze propellant (search terms included “Condition: Chronic Obstructive Pulmonary Disease (COPD)”, “Other Terms: Safety and Tolerability”, “Study Status: Completed”, “Study Results: With Results”, “Study Phase: Phase 3”). A total of 21 studies were identified, 14 of which had published manuscripts; six of these manuscripts were written with a primary intent to discuss safety data; the remaining eight focused first on efficacy but did include some safety results. Here, to further understand the safety of current versus HFO-1234ze propellants, and in accordance with regulatory guidance, we reported on the safety of HFO-1234ze, compared with HFA-134a when used in the delivery of budesonide/glycopyrronium/formoterol fumarate dihydrate (BGF) in participants with COPD.

**Added value of this study**

Participants with COPD receiving BGF formulated with HFO-1234ze or HFA-134a (two inhalations of 160/7·2/5·0 μg twice daily) experienced generally similar rates of adverse events (including respiratory events of special interest) and had no differences in laboratory outcomes or electrocardiogram monitoring; this suggests a comparable safety profile between propellants.

**Implications of all the available evidence**

These results support the use of HFO-1234ze versus the currently marketed HFA-134a in the BGF pMDI. Further evaluation in a real-world setting would provide information on the safety and tolerability of the HFO-1234ze propellant in broader populations and over longer term chronic usage.

## Introduction

Many people living with respiratory diseases, such as chronic obstructive pulmonary disease (COPD), rely on therapies delivered by pressurised metered dose inhalers (pMDIs).^1^ Those with moderate-to-severe disease may be better treated with pMDI-based therapies as they often cannot generate sufficiently high inspiratory flow rate to use dry powder inhalers.^2^ All pMDIs use propellants to facilitate pulmonary drug delivery.^1^ Healthcare's environmental impact is under growing scrutiny, posing challenges in balancing sustainability, patient safety and autonomy.^3^ Inhaler prescribing with respect to their propellants, exemplifies tensions between reducing environmental footprint and maintaining treatment quality.^3^

Budesonide/glycopyrronium/formoterol fumarate dihydrate (BGF) is a triple therapy pMDI approved as a maintenance therapy for patients with COPD and is formulated with a hydrofluoroalkane-134a (HFA-134a) propellant.^4^ Like most other propellants used in pMDIs, HFA-134a is a hydrofluorocarbon known to have global warming potential (GWP).^5^^,^^6^ As such, next generation propellants with lower GWP are needed to reduce the environmental impact of pMDIs whilst safeguarding access to essential medicines.^5^^,^^7^^,^^8^

In pivotal phase 3 studies (ETHOS and KRONOS), BGF 320/14·4/10 μg (equivalent to budesonide/glycopyrrolate/formoterol fumarate 320/18/9·6 μg) improved lung function^9^^,^^10^ and reduced the annual rate of COPD exacerbations^10^^,^^11^ in patients with moderate-to-very severe COPD versus dual therapy with the long-acting muscarinic antagonist (LAMA)/long-acting β_2_-agonist (LABA) glycopyrronium/formoterol fumarate dihydrate (GFF) 14·4/10 μg and the inhaled corticosteroid (ICS)/LABA budesonide/formoterol fumarate dihydrate (BFF) 320/10 μg. The safety profile of BGF versus GFF and BFF was similar across ETHOS and KRONOS studies.^10^^,^^11^

Hydrofluoroolefin-1234ze (HFO-1234ze) is in development for use as a propellant in pMDIs and has a GWP 99·9% lower than that of HFA-134a.^12^^,^^13^ In preclinical studies, HFO-1234ze was only detected in the blood transiently despite continued nebulisation with high doses of the propellant and was not associated with safety findings considered to pose a risk to patients at exposure levels associated with pMDI use.^14^ In phase 1 studies of healthy adults, total systemic^15^ and lung exposure^16^ to BGF components was bioequivalent when formulated with HFO-1234ze versus the HFA-134a propellant; a similar safety profile was observed for HFO-1234ze and HFA-134a, with no new or unexpected findings.^8^^,^^15^^,^^16^

Here, we assessed whether the safety profiles were similar for participants with COPD receiving BGF formulated with the HFO-1234ze versus HFA-134a propellant for 12 and 52 weeks.

## Methods

### Study design

This was a phase 3, multi-site, multi-country, randomised, double-blind, parallel-group, active-comparator, 12-week treatment study (with a prespecified cohort assigned to 52 weeks of treatment) comparing the safety of BGF 320/14·4/10 μg (equivalent to budesonide/glycopyrrolate/formoterol fumarate 320/18/9·6 μg) with HFO-1234ze versus HFA-134a (both delivered via pMDI) in adults with COPD (ClinicalTrials.gov registry: NCT05573464). The study was conducted in 92 research sites across 9 countries (Argentina, Bulgaria, Canada, Germany, Mexico, Poland, Turkey, the United Kingdom, the United States).

### Ethics statement

The study was conducted in accordance with the ethical principles of the Declaration of Helsinki and consistent with International Council for Harmonisation/Good Clinical Practice, applicable regulatory requirements, and AstraZeneca policy on bioethics. The study protocol and participant informed consent documents were reviewed and approved by an institutional review board or independent ethics committee (see Supplementary Table S1). Participants or their legally authorised representatives were required to sign informed consent statements, with participants informed their participation was voluntary.

### Participants

A full listing of the inclusion and exclusion criteria is provided in the Supplementary Material. Briefly, to be eligible for inclusion, patients had to be: aged 40–80 years (inclusive); with a documented history of physician-diagnosed COPD; stable dosing of dual (ICS/LABA or LAMA/LABA) or triple (ICS/LAMA/LABA, open or fixed-dose combinations) inhaled COPD maintenance therapies for ≥6 weeks before screening; a pre-bronchodilator forced expiratory volume in 1 s (FEV_1_) of <80% predicted normal at visit 1 (screening); a post-bronchodilator FEV_1_/forced vital capacity (FVC) ratio of <0·70 and post-bronchodilator FEV_1_ of ≥25%–<80% predicted normal at visit 2 (during the screening period); a COPD Assessment Test (CAT) score ≥10 at visit 1 (during the screening period); a current/former smoker with a history of ≥10 pack-years of tobacco smoking; and demonstrate acceptable pMDI administration and spirometry techniques.

Key exclusion criteria included a diagnosis of asthma within 5 years of visit 1; alpha-1 antitrypsin deficiency; a requirement for chronic oxygen therapy or positive pressure ventilatory support; a moderate or severe exacerbation of COPD, or a respiratory infection ending within 4 weeks before visit 1, or during screening; and unstable cardiovascular (CV) disease.

Information on sex/gender was self-reported by the participant and then entered by the Investigator into the Interactive Response Technology (IRT) system. No genetic testing was required to define sex/gender.

### Randomisation and masking

Participants were randomised (1:1) using a centralised IRT/Randomization and Trial Supply Management (RTSM) system. Randomisation was stratified by study region (Americas versus Europe), and COPD disease severity (percent predicted FEV_1_ ≥50% versus <50%). The sponsor, investigator, all clinical staff involved in the clinical study, participants, and the study monitor were blinded, with the IRT programmed with blind-breaking instructions, unless safety concerns or a regulatory requirement necessitate unblinding. The Supply Chain Study Manager, who is unblinded to manage drug supply effectively and perform transactions that take effect immediately, may have access to the RTSM system.

Packaging and labelling materials for both HFO-1234ze and HFA-134a are identical in appearance, thus there is no possibility to recognise the treatment arm based on physical appearance.

### Procedures

Randomised participants received the approved BGF dose for the maintenance treatment of COPD (BGF 160/7·2/5·0 μg, two inhalations twice daily) using HFO-1234ze or HFA-134a as the propellant. To evaluate any safety considerations that may be more relevant with longer-term dosing, the first 120 participants randomised to each treatment (total of 240) were prespecified to receive 52 weeks of double-blinded study intervention (52-week safety analysis set); all other participants received 12 weeks of double-blinded study intervention, representing the main safety analysis set (Supplementary Fig. S1). Study site personnel were provided instructions on appropriate inhaler technique using a training video and printed instructions; staff were responsible for teaching and enforcing appropriate inhaler usage for study participants throughout the trial. Participants had access to printed inhaler usage instructions and proper technique was assessed using placebo pMDIs. Inhaler usage and maintenance was assessed at all scheduled, in-person clinic visits, and re-training was provided if necessary. Spacers were not used. All participants received a country-approved, locally sourced, study-provided albuterol/salbutamol pMDI with an HFA-based propellant as a rescue medication for use as needed throughout the screening and treatment periods.

### Outcomes

#### Primary endpoints

Primary safety endpoints assessed were overall incidence of adverse events (AEs), including serious AEs (SAEs), severe AEs, discontinuations due to an AE, AEs of special interest (respiratory events such as e.g., COPD, dysphonia, cough, dyspnoea, and increased sputum), and most frequently reported AEs by Preferred Term based on Medical Dictionary for Regulatory Activities (MedDRA) version 26·1. AEs of special interest were selected given that respiratory AEs were considered more relevant to the evaluation of an inhaled propellant with very limited systemic exposure, in the setting of equivalent pharmacokinetics for the active pharmaceutical ingredients. AEs were recorded from randomisation (SAEs were recorded from the time the informed consent form was signed), throughout the treatment period (every 2 weeks via telephone in between on-site visits) and the follow-up period, up to the final visit (follow-up call, 2 weeks after last investigational medicinal product/study intervention dose).

Continuous 24-h digital 12-lead Holter electrocardiogram (ECG) monitoring occurred at randomisation to detect baseline cardiac rhythms/arrhythmias. The recording also covered the first inhaled dosing occasion (to detect potential immediate post-dose cardiac events linked to the propellant) and continued for up to 6 h post-dosing when the patient left the clinic. The Holter ECG recordings were initiated 1 day prior to scheduled visits 3 and 6 at approximately the same time (13:00 ± 2 h) for both visits covering 4 h post dosing. Continuous 24-h digital 12-lead Holter ECG monitoring was then repeated at the 12-week visit, and change from baseline in rhythm parameters were evaluated.

Triplicate 12-lead ECGs (stand-alone resting digital ECGs or extracted from the 12-lead Holter digital ECGs described above) were assessed at visits 3 (week 1), 4 (week 4), 5 (week 8), and 6 (week 12); and at visit 9 (week 52) for participants treated for 52 weeks. Parameters assessed (absolute values and change from baseline at each post-baseline visit) included evaluation of clinically significant ECG changes, such as new-onset arrhythmias and QT interval prolongation.

Clinical laboratory tests (haematology, clinical chemistry, estimated glomerular filtration rate) were performed and vital signs were assessed (absolute values and change from baseline) throughout the treatment and follow-up periods.

#### Exploratory endpoints

The following exploratory endpoints were evaluated: a) spirometry to measure change from baseline (randomisation) in FEV_1_ at 12 and 52 weeks (specifically, values at 30 min pre-dose and 5, 15, 30, and 60 min post-dose were compared with the pre-dose value obtained at randomisation); and b) change from baseline in CAT total score at 12 and 52 weeks.^17^

### Statistical analysis

Analyses focused on comparisons between BGF formulated with the HFO-1234ze versus HFA-134a. Statistical analyses were performed using SAS® Version 9·4. There was no data monitoring committee for this study.

The 12-week safety analysis set consisted of all participants randomised to study treatment and who received ≥1 study drug dose, irrespective of protocol adherence and whether they were prespecified to continue treatment to 52 weeks. It was estimated that a sample size of 542 randomised 1:1 provided 90% probability of observing ≥1 AE in each treatment group with an expected cumulative incidence of 1%, and assuming a 15% dropout rate.

The 52-week safety analysis set consisted of the first 120 participants randomised to each treatment group from the 12-week safety analysis set who received ≥1 dose and were prespecified to continue treatment for 52 weeks, irrespective of protocol adherence. Accounting for dropouts, the aim was to achieve approximately 100 participants per treatment group completing 52 weeks.

The 12- and 52-week safety analysis sets were not mutually exclusive; for example, if a participant prespecified to receive 52 weeks of treatment had an AE during the first 12 weeks of treatment, the AE would be reported in both the 12-week and 52-week safety analysis sets.

FEV_1_ and change from baseline in CAT total score were evaluated descriptively. Changes in CAT total score and FEV_1_ at a given time point were compared with baseline values obtained when participants received their standard treatment during the run-in period. Post hoc analyses of changes from baseline in trough FEV_1_ and FEV_1_ area under the curve from 0 to 60 min post-dose (FEV_1_ AUC_0‒60_), with adjusted absolute differences in mean change and 95% confidence intervals (CIs) for HFO-1234ze relative to HFA-134a, are reported for weeks 12 and 52. Baseline CAT values and spirometry measures were obtained during established treatment regimens prior to randomisation to HFO-1234ze or HFA-134a regimens.

### Role of the funding source

The study sponsor was involved in the study design; the collection, analysis, and interpretation of data; and in the writing of the manuscript. The decision to publish was a joint decision among the authors, including those employed by the sponsor.

## Results

### Participant disposition and demographics

**Fig. 1:**
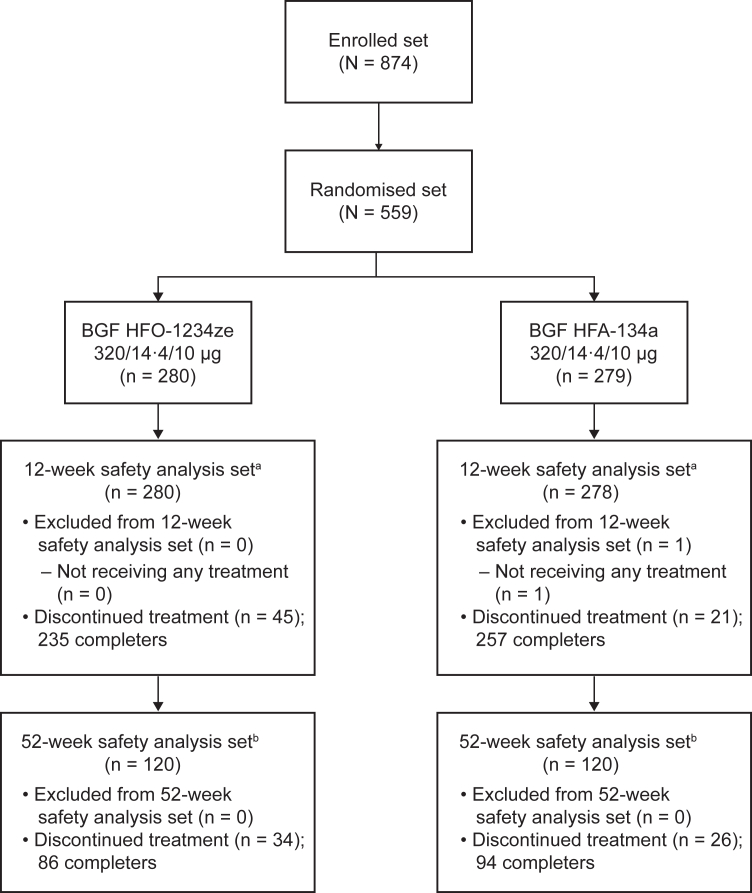
**Participant disposition.**^a^The 12-week safety analysis set is defined as all participants who were randomised to study treatment and received ≥1 inhalation of study drug, irrespective of their protocol adherence and whether they would continue the extended study. ^b^The 52-week safety analysis set is defined as all participants who were randomised to study treatment received ≥1 inhalation of study drug and were selected to continue treatment for a total of 52 weeks. The same participant could have been excluded from an analysis set for more than one reason. BGF, budesonide/glycopyrronium/formoterol fumarate dihydrate; HFA-134a, hydrofluoroalkane-134a; HFO-1234ze, hydrofluoroolefin-1234ze.

Participants were recruited between 27 September 2022 and 19 May 2023. Participant disposition is summarised in Fig. 1. A total of 874 participants were recruited. Of the 559 randomised participants (HFO-1234ze, n = 280; HFA-134a, n = 279), 558 received treatment and were included in the 12-week safety analysis set (HFO-1234ze, n = 280; HFA-134a, n = 278), and 240 were included in the 52-week safety analysis set (HFO-1234ze, n = 120; HFA-134a, n = 120). There were more discontinuations in the HFO-1234ze arm (n = 45 in the 12-week safety analysis set and n = 34 in the 52-week safety analysis set) compared with the HFA-134a arm (n = 21 in the 12-week safety analysis set and n = 26 in the 52-week safety analysis set); there was no overall pattern observed in the reasons for participant discontinuations.

Demographics, disease characteristics, medical history, and other baseline assessments were similar between treatment groups and the 12- and 52- week safety analysis sets (Table 1). In the 12 and 52-week safety analysis sets, respectively, mean age (standard deviation [SD]) was 67·0 (7·4) and 67·4 (7·2) years; and just over half of participants were male (315 [56·5%] and 137 [57·1%]) and former smokers (324 [58·1%] and 131 [54·6%]). Given the advanced age of the target population and the severity of their COPD, there were several comorbidities, with the majority of participants in each cohort having a CV history at randomisation (data not shown).

**Table 1:** Demographics, disease characteristics, medical history, and other baseline assessments in the 12- and 52-week safety analysis sets.

	BGF HFO-1234ze 320/14·4/10 μg	BGF HFA-134a 320/14·4/10 μg	Total
***12-week safety analysis set***	**N = 280**	**N = 278**	**N = 558**

**Age, years, mean (SD)**	67·1 (7·7)	67·0 (7·0)	67·0 (7·4)
**Sex, male, n (%)**	168 (60·0)	147 (52·9)	315 (56·5)
**Race, n (%)**			
Black or African American	7 (2·5)	4 (1·4)	11 (2·0)
American Indian or Alaska Native	0	1 (0·4)	1 (0·2)
White	273 (97·5)	272 (97·8)	545 (97·7)
Multiple	0	1 (0·4)	1 (0·2)
**COPD medications at screening,**^a^**n (%)**			
ICS/LABA	109 (38·9)	105 (37·8)	214 (38·4)
LAMA/LABA	87 (31·1)	90 (32·4)	177 (31·7)
ICS/LAMA/LABA	90 (32·1)	85 (30·6)	175 (31·4)
SABA	237 (84·6)	238 (85·6)	475 (85·1)
**Baseline pre-bronchodilator FEV_1_,%predicted, mean (SD)**	46·9 (13·5)	46·2 (15·5)	46·6 (14·5)
**Number of pack-years smoked,**^b^**mean (SD)**	45·3 (22·7)	46·7 (23·6)	46·0 (23·1)
**Smoking history, n (%)**			
Current	120 (42·9)	114 (41·0)	234 (41·9)
Former	160 (57·1)	164 (59·0)	324 (58·1)
**COPD severity, n (%)**			
Mild	0	1 (0·4)	1 (0·2)
Moderate	154 (55·0)	144 (51·8)	298 (53·4)
Severe	111 (39·6)	109 (39·2)	220 (39·4)
Very severe	15 (5·4)	24 (8·6)	39 (7·0)
***52-week safety analysis set***	**N = 120**	**N = 120**	**N = 240**

**Age, years, mean (SD)**	67·2 (7·7)	67·7 (6·6)	67·4 (7·2)
**Sex, male, n (%)**	69 (57·5)	68 (56·7)	137 (57·1)
**Race, n (%)**			
Black or African American	4 (3·3)	2 (1·7)	6 (2·5)
American Indian or Alaska Native	0	1 (0·8)	1 (0·4)
White	116 (96·7)	117 (97·5)	233 (97·1)
**COPD medications at screening,**^a^**n (%)**			
ICS/LABA	49 (40·8)	45 (37·5)	94 (39·2)
LAMA/LABA	42 (35·0)	49 (40·8)	91 (37·9)
ICS/LAMA/LABA	32 (26·7)	27 (22·5)	59 (24·6)
SABA	107 (89·2)	103 (85·8)	210 (87·5)
**Baseline pre-bronchodilator FEV_1_,%predicted, mean (SD)**	47·4 (13·6)	46·4 (13·8)	46·9 (13·7)
**Number of pack-years smoked,**^b^**mean (SD)**	46·3 (23·9)	48·6 (25·5)	47·4 (24·7)
**Smoking history, n (%)**			
Current	53 (44·2)	56 (46·7)	109 (45·4)
Former	67 (55·8)	64 (53·3)	131 (54·6)
**COPD severity, n(%)**			
Moderate	68 (56·7)	65 (54·2)	133 (55·4)
Severe	47 (39·2)	45 (37·5)	92 (38·3)
Very severe	5 (4·2)	10 (8·3)	15 (6·3)

BGF, budesonide/glycopyrronium/formoterol fumarate dihydrate; COPD, chronic obstructive pulmonary disease; FEV_1_, forced expiratory volume in 1 s; HFA-134a, hydrofluoroalkane-134a; HFO-1234ze, hydrofluoroolefin-1234ze; ICS, inhaled corticosteroid; LABA, long-acting β_2_-agonist; LAMA, long-acting muscarinic antagonist; N, total number of participants in treatment group; n, number of participants in subcategory; SABA, short-acting β_2_-agonist; SD, standard deviation.

a Single or combination chronic obstructive pulmonary disease (COPD) medication regimens at screening included the history of usage 6 weeks before screening; maintenance COPD medications mapped to eligibility criteria and resulted in some participants being assigned to ≥2 regimens if medications were altered during the 6 months before enrolment. All eligible participants were on a fixed COPD medication regimen for ≥6 weeks before prior to screening.

b Number of pack-years smoked = (number of cigarettes per day ÷ 20) × number of years smoked.

### AEs

#### Overall summary of AEs

**Fig. 2:**
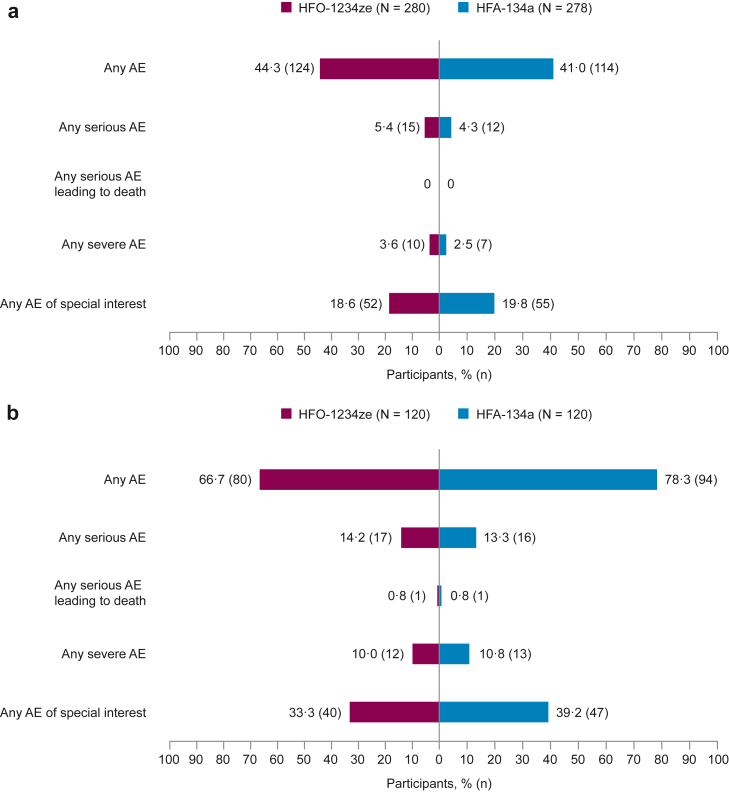
**Overall summary of adverse events (AEs) in the 12-week safety analysis set (a) and the 52-week safety analysis set (b).** Adverse events (AEs) with an onset date on or after the date of the first dose of investigational product (IP) throughout the treatment period up to and including the date of the last IP dose + 1 day. Participants with multiple occurrences in the same category are counted once per category regardless of the number of occurrences. AE, adverse event; HFA-134a, hydrofluoroalkane-134a; HFO-1234ze, hydrofluoroolefin-1234ze; IP, investigational product; N, total number of participants in treatment group; n, number of participants with AE.

In the 12-week safety analysis set, a similar proportion of participants reported ≥1 AE in the two treatment groups (HFO-1234ze, 124 [44·3%]; HFA-134a, 114 [41·0%]) (Fig. 2a). The proportion of participants experiencing any severe AE (HFO-1234ze, 10 [3·6%]; HFA-134a, 7 [2·5%]) or any AE of special interest (HFO-1234ze, 52 [18·6%]; HFA-134a, 55 [19·8%]) was also similar in the two treatment groups (Fig. 2a).

In the 52-week safety analysis set, 66·7% of participants (n = 80) receiving HFO-1234ze and 78·3% (n = 94) receiving HFA-134a reported ≥1 AE (Fig. 2b). The proportion of participants experiencing any severe AE (HFO-1234ze, 12 [10·0%]; HFA-134a, 13 [10·8%]) or any AE of special interest (HFO-1234ze, 40 [33·3%]; HFA-134a, 47 [39·2%]) was also similar in the two treatment groups.

Investigators assessed the maximum intensity of AEs for most participants as mild or moderate, with similar proportions across treatment groups.

#### Severe AEs

Severe AEs accounted for a small proportion of all reported AEs in both treatment groups in the 12- and 52-week safety analysis sets (as shown in Fig. 2). Table 2 shows severe AEs occurring in ≥2 participants in any treatment group by the System Organ Class and Preferred Term. In the 12-week analysis set, there were three participants with six severe cardiac AEs in the HFO-1234ze group and no participants with severe cardiac AEs in the HFA-134a group. In the 52-week analysis set, there was one participant with a severe cardiac event in the HFO-1234ze group and two participants with four severe cardiac events in the HFA-134a group. All participants with severe cardiac AEs had a history of CV disease prior to study enrolment.

**Table 2:** Participants with any severe adverse event (AE) and severe AEs by System Organ Class and Preferred Term (Medical Dictionary for Regulatory Activities [MedDRA] version 26·1) across 12 and 52 weeks^a^^,^^b^ (reported in ≥2 participants in any treatment group by System Organ Class).

***12-week safety analysis set***	BGF HFO-1234ze 320/14·4/10 μg		BGF HFA-134a 320/14·4/10 μg	
	**N = 280** **n (%)**^b^	Number of events per category	**N = 278** **n (%)**^b^	Number of events per category

**Any severe AE**	10 (3·6)	18	7 (2·5)	7
**Infections and infestations**	4 (1·4)	4	1 (0·4)	1
COVID-19	1 (0·4)	1	1 (0·4)	1
Pneumonia	1 (0·4)	1	0	–
Pneumonia haemophilus	1 (0·4)	1	0	–
Pneumonia streptococcal	1 (0·4)	1	0	–
**Cardiac disorders**	3 (1·1)	6	0	–
Acute myocardial infarction	2 (0·7)	2	0	–
Atrial fibrillation	2 (0·7)	2	0	–
Atrial flutter	1 (0·4)	1	0	–
Cardiac failure	1 (0·4)	1	0	–
**Respiratory thoracic and mediastinal disorders**	4 (1·4)	4	5 (1·8)	5
Acute pulmonary oedema	1 (0·4)	1	0	–
COPD	2 (0·7)	2	5 (1·8)	5
Cough	1 (0·4)	1	0	–
***52-week safety analysis set***	**N = 120** **n (%)**^b^		**N = 120** **n (%)**^b^	

**Any severe AE**	12 (10·0)	17	13 (10·8)	24
**Infections and infestations**	4 (3·3)	5	3 (2·5)	4
Atypical pneumonia	1 (0·8)	1	0	–
COVID-19	0	–	2 (1·7)	2
Pneumonia	1 (0·8)	1	1 (0·8)	1
Pneumonia haemophilus	1 (0·8)	1	0	–
Pneumonia pneumococcal	0	–	1 (0·8)	1
Bacterial respiratory tract infection	1 (0·8)	1	0	–
Sepsis	1 (0·8)	1	0	–
**Cardiac disorders**	1 (0·8)	1	2 (1·7)	4
Acute myocardial infarction	0	–	1 (0·8)	1
Angina unstable	0	–	1 (0·8)	1
Atrial fibrillation	1 (0·8)	1	0	–
Atrial flutter	0	–	1 (0·8)	1
Cardiac failure congestive	0	–	1 (0·8)	1
**Respiratory thoracic and mediastinal disorders**	7 (5·8)	8	8 (6·7)	8
Acute respiratory failure	1 (0·8)	1	0	–
COPD	5 (4·2)	5	6 (5·0)	6
Cough	1 (0·8)	1	0	–
Haemothorax	0	–	1 (0·8)	1
Pulmonary embolism	0	–	1 (0·8)	1
Respiratory distress	1 (0·8)	1	0	–
**Musculoskeletal and connective tissue disorders**	1 (0·8)	1	2 (1·7)	2
Mobility decreased	0	–	1 (0·8)	1
Osteoarthritis	1 (0·8)	1	1 (0·8)	1
**Injury, poisoning, and procedural complications**	0	–	2 (1·7)	3
Fall	0	–	2 (1·7)	2
Hip fracture	0	–	1 (0·8)	1

AE, adverse event; BGF, budesonide/glycopyrronium/formoterol fumarate dihydrate; COPD, chronic obstructive pulmonary disease; COVID-19, Coronavirus Disease 2019; HFA-134a, hydrofluoroalkane-134a; HFO-1234ze, hydrofluoroolefin-1234ze; IP, investigational product; MedDRA, Medical Dictionary for Regulatory Activities; N, total number of participants in treatment group; n, number of participants with AE.

a Adverse events (AEs) with an onset date on or after the date of the first dose of investigational product (IP) throughout the treatment period up to and including the date of the last IP dose + 1 day.

b Participants with multiple occurrences are counted once per system organ class and preferred term regardless of the number of occurrences.

#### SAEs

The pattern of SAEs was similar to severe AEs between HFO-1234ze and HFA-134a treatments for both the 12- and 52-week safety analysis sets (Supplementary Table S2).

#### Discontinuations due to an AE

In the 12-week safety analysis set, there was a higher proportion of discontinuations due to an AE reported in the HFO-1234ze group (20 [7·1%]) compared with the HFA-134a group (9 [3·2%]) (Supplementary Table S3). In the 52-week safety analysis set, the incidence of discontinuations due to an AE in the two treatment arms was similar (HFO-1234ze, 11 [9·2%]; HFA-134a, 9 [7·5%]). However, within the 52-week safety analysis set, after completing the 12-week treatment period, discontinuations due to an AE were only reported in those assigned to HFA-134a (incidence: 6 [5·0%]), with no reports of discontinuations due to an AE in those assigned to HFO-1234ze (incidence: 0 [0·0%]). For both the 12- and 52-week safety analysis sets, there was no overall pattern to reasons for participant discontinuations between treatment groups when discontinuations due to an AE were evaluated by System Organ Class and Preferred Term (Supplementary Table S3).

#### AEs of special interest

All AEs of special interest occurring in the 12- and 52-week analysis sets are shown in Supplementary Table S4. The most frequently reported AEs of special interest were comparable between treatment groups in both 12- and 52-week safety analysis sets and included COPD (defined by worsening symptoms or exacerbations) (12-week: HFO-1234ze, 39 [13·9%]; HFA-134a, 40 [14·4%], 52-week: HFO-1234ze, 34 [28·3%]; HFA-134a, 43 [35·8%]) and dysphonia (12-week: HFO-1234ze, 11 [3·9%]; HFA-134a, 8 [2·9%], 52-week: HFO-1234ze, 5 [4·2%]; HFA-134a, 3 [2·5%]) (Supplementary Table S4).

#### Most frequently reported AEs

**Fig. 3:**
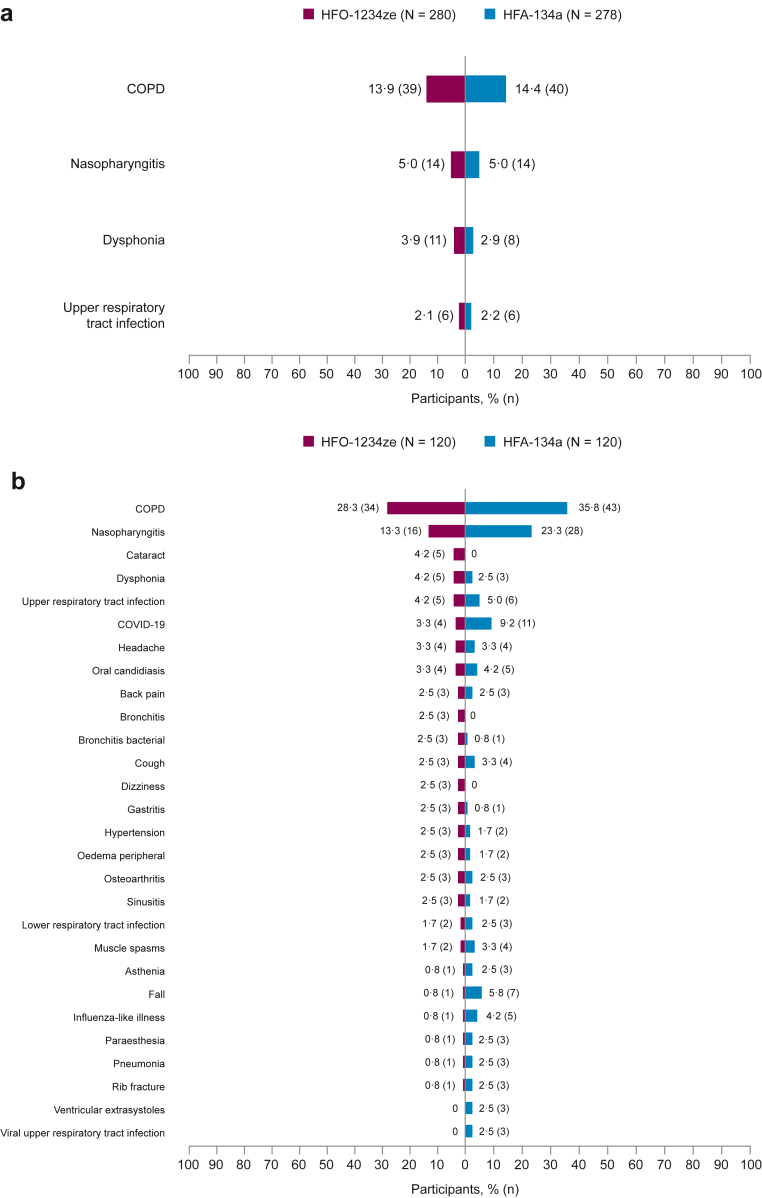
**Most frequently reported adverse events (AEs) by Preferred Term (Medical Dictionary for Regulatory Activities [MedDRA] version 26·1) in the 12-week safety analysis (a) and 52-week safety analysis set (b) (reported in ≥2 participants in any treatment group).** Adverse events (AEs) with an onset date on or after the date of the first dose of investigational product (IP) throughout the treatment period up to and including the date of the last IP dose + 1 day. Participants with multiple occurrences in the same category are counted once per category regardless of the number of occurrences. AE, adverse event; COVID-19, Coronavirus Disease 2019; HFA-134a, hydrofluoroalkane-134a; HFO-1234ze, hydrofluoroolefin-1234ze; IP, investigational product; MedDRA, Medical Dictionary for Regulatory Activities; N, total number of participants in treatment group; n, number of participants with AE.

The most frequently reported AEs (those occurring in ≥2 of participants in any treatment group by Preferred Term) occurred in similar proportions in both treatment groups for the 12-week safety analysis set; and included COPD (defined by worsening symptoms or exacerbations), nasopharyngitis, dysphonia, and upper respiratory tract infection (Fig. 3a). In both treatment groups of the 52-week safety analysis set, COPD, nasopharyngitis, Coronavirus Disease 2019 (COVID-19), and upper respiratory tract infection were the most frequently reported AEs (Fig. 3b); however, fewer participants receiving HFO-1234ze versus HFA-134a reported COPD, nasopharyngitis, and upper respiratory tract infection. Other AEs reported in ≥2 of participants in any treatment group by Preferred Term in the 52-week safety analysis set occurred in similar proportions for those receiving HFO-1234ze versus HFA-134a.

### Other safety endpoints

There were no clinically meaningful changes between treatment groups in the digital 12-lead Holter ECG monitoring (in the 12-week safety analysis set) or in 12-lead digital ECGs (in the 12- and 52-week analysis sets), in clinical laboratory tests (in the 12- and 52-week analysis sets), or in vital signs (in the 12- and 52-week analysis sets). There were no deaths in the 12-week safety analysis set. Two participants in the 52-week safety analysis set, one in each treatment arm, died after completing 12 weeks of treatment. One participant (HFO-1234ze group) died following an ischaemic stroke, at day 296 after the first dose of treatment. A second participant (HFA-134a group) died following an acute myocardial infarction at day 265 after the first dose of treatment. These deaths were deemed not related to treatment.

### Exploratory endpoints

#### Lung function change from baseline

Changes from baseline in trough FEV_1_ over the treatment periods were similar between treatment groups in the 12-week (least squares mean [LSM] (standard error [SE]): HFO-1234ze, −0·009 L (0·012); HFA-134a, 0·003 L (0·012)) and 52-week (LSM (SE): HFO-1234ze, −0·031 L (0·017); HFA-134a, −0·018 L (0·016)) safety analysis sets, with the difference in mean changes from baseline for HFO-1234ze relative to HFA-134a being −0·012 L (95% CI: −0·045, 0·021) and −0·013 L (95% CI: −0·060, 0·033), respectively. Changes from baseline in FEV_1_ AUC_0‒60_ over the treatment periods were also similar between treatment groups in the 12-week (LSM (SE): HFO-1234ze, 0·132 L (0·010); HFA-134a, 0·143 L (0·010)) and 52-week (LSM (SE): HFO-1234ze, 0·111 L (0·015); HFA-134a, 0·127 L (0·015)) safety analysis sets, with the difference in mean changes from baseline for HFO-1234ze relative to HFA-134a being −0·011 L (95% CI: −0·039, 0·017) and −0·015 L (95% CI: −0·058, 0·027), respectively.

#### Change from baseline in CAT total score

Clinical response to study treatment, assessed by changes from baseline in CAT total score, in participants from the 12-week safety analysis set trended toward improvement. The mean (SD) change from baseline CAT total score at 12 weeks was −3·4 (5·6) for HFO-1234ze and −2·7 (5·7) for HFA-134a. Similar trends were observed among participants in the 52-week safety analysis set, in whom mean (SD) changes from baseline in CAT total score were −3·1 (6·0) for HFO-1234ze and −2·4 (5·4) for HFA-134a at 12 weeks and −2·7 (7·0) for HFO-1234ze and −3·0 (6·9) for HFA-134a at 52 weeks.

## Discussion

This phase 3 study assessed the safety of BGF pMDI formulated with the next generation propellant HFO-1234ze in patients with COPD. The study found that the safety of BGF HFO-1234ze, which has a GWP >99·9% lower than HFA-134a,^12^^,^^13^ is comparable to the currently approved BGF HFA-134a formulation across 12 weeks and 52 weeks of treatment. Importantly, there were no new or unexpected safety signals in those receiving BGF HFO-1234ze. The results give confidence to healthcare professionals and COPD patients reliant on their essential pMDIs for everyday disease management, towards transitioning to pMDIs with a lower GWP, thus safeguarding environmental concerns. Indeed, patients with COPD are a vulnerable population recognised to experience poor health outcomes through a combination of factors, including pulmonary disease, comorbidities, and advancing age.^18^, ^19^, ^20^ Obtaining optimal clinical benefit from the drugs by virtue of their inhaler device, whilst achieving a good therapeutic ratio is critical.

Per the previous ETHOS and KRONOS studies,^10^^,^^11^ a variety of AEs were reported in the 12- and 52-week safety analysis sets of this study. The most frequently reported AEs in this study were generally comparable to the ETHOS and KRONOS studies, and included COPD, nasopharyngitis, and upper respiratory tract infections.^10^^,^^11^ Here, exploratory clinical measures of lung function and of COPD impact on health status indicated that HFO-1234ze and HFA-134a had comparable therapeutic effects. The design of this study also facilitated assessment of AEs of special interest specifically related to an HFO propellant switch and included COPD, dysphonia, cough, dyspnoea, and increased sputum. Reassuringly, there was no difference in the frequency of AEs of special interest between the two treatment groups. The similar safety profile for both BGF formulations observed here aligns with data from lung and systemic bioequivalence studies of BGF HFO-1234ze versus BGF HFA-134a.^15^^,^^16^ Specifically, in studies of healthy adults, all three active pharmaceutical ingredients in BGF HFO-1234ze were bioequivalent to BGF HFA-134a, with no new or unexpected safety findings.^8^^,^^15^^,^^16^

In the 12-week safety analysis set, three participants had six severe cardiac AEs in the HFO-1234ze group. However, all three participants had pre-existing medical histories of CV disease. Further, there were no clinically relevant post-dose changes in 12-lead ECGs in the overall study population. Additionally, participants had ECG monitoring for CV safety assessments at the time of first treatment, after treatment initiation, and after multiple treatments over the course of 12 weeks. There was no difference in CV safety based on the ECG monitoring; this was expected given the transient propellant having an extremely short exposure time. These findings align with previous nonclinical/preclinical reports showing a lack of acute ECG effects with HFO-1234ze and no CV events at this dosing.^14^ Moreover, across 52 weeks of treatment, more instances of severe AEs related to cardiac disorders were observed in the HFA-134a group. Lastly, the observed CV-related AEs in this study were either known or expected comorbidities in a population of individuals with COPD. Therefore, the numerical imbalance in severe cardiac AEs in the 12-week cohort is most likely explained by CV issues existing before randomisation.

It was noted that a greater proportion of participants receiving BGF with HFO-1234ze than with HFA-134a experienced discontinuations due to an AE in the 12-week safety analysis set and also most patients discontinued from the study for any reason, including non-clinical reasons, in both 12- and 52-weeks data sets. The discontinuations due to an AE were due to a broad set of reasons, with no single category driving the results (See Supplementary Table S3). Similarly, there was no clear pattern in the study discontinuations for other reasons. Difference in physico-chemical properties between HFO-1234ze and HFA-134a could potentially account for overall pattern in discontinuations. In a prior study, healthy volunteers did not perceive differences in taste between HFO-1234ze and HFA-134a^8^; however, differences in physico-chemical perception were noted following the switch from chlorofluorocarbon to HFA propellants from the late 2000s.^21^ Furthermore, all participants were very likely to have had experience of using the HFA-134a propellant driven inhalers as further supported by the provision of available marketed HFA albuterol/salbutamol for reliever use.

A strength of this study is that there were limited barriers for inclusion, and the cohorts were representative of the general population of patients with COPD. In addition, participants had detailed cardiac rhythm and ECG monitoring (including 12-lead digital Holter monitoring) throughout the study, which enabled detection of potential adverse CV effects from the propellant having an extremely short exposure time. In terms of limitations, since participants did not stop their standard COPD treatment until visit 3 (week 1), their baseline lung function assessment occurred on heterogenous background therapy, so any changes from baseline in lung function should be interpreted with caution. However, it should be noted that the HFO-1234ze and HFA-134a groups showed comparable values for trough and post-dose assessments. Secondly, most of the study participants were White, possibly limiting the generalisability of the findings. Lastly, the number of participants included with very severe COPD was low, rendering it difficult to draw conclusions about differences in safety between propellants in this population.

In conclusion, the safety findings from this study indicate a similar benefit-risk profile for both HFO-1234ze and HFA-134a propellant pMDIs, thus supporting the use of HFO-1234ze versus the currently marketed HFA-134a in the BGF pMDI. Importantly, the data support transitioning to pMDIs with propellants that have a lower GWP, thus reducing the environmental impact of pMDIs.

## Contributors

Author contributions to the development of the current manuscript are as follows: **accessed and verified the data** (Omar S. Usmani, Hitesh Pandya, Matthew Camiolo, Artur Bednarczyk, Christer Gottfridsson, Lars Pettersson, Jie Mei, Karin Skansen, Jennifer L. Bell, David Petullo, Mandeep Jassal, Mehul Patel), **conceptualisation** (Omar S. Usmani, Artur Bednarczyk, Christer Gottfridsson, Kathryn Collison, Mehul Patel), **data curation** (Hitesh Pandya, Matthew Camiolo, Mandeep Jassal, Mehul Patel), **formal analysis** (Christer Gottfridsson, Lars Pettersson, Jie Mei, Karin Skansen, Jennifer L. Bell, David Petullo), **funding acquisition** (Kathryn Collison, Patrik Bondarov, Mehul Patel), **investigation** (Hitesh Pandya, Artur Bednarczyk, Kinga Kucz, Matthew Camiolo, Christer Gottfridsson, Lars Pettersson, Jie Mei, Karin Skansen, Mandeep Jassal, Mehul Patel), **methodology** (Omar S. Usmani, Hitesh Pandya, Artur Bednarczyk, Christer Gottfridsson, Jennifer L. Bell, David Petullo, Mehul Patel), **project administration** (Hitesh Pandya, Artur Bednarczyk, Marek Kokot, Kathryn Collison, Patrik Bondarov, Mehul Patel), **resources** (Hitesh Pandya, Kathryn Collison, Patrik Bondarov, Mehul Patel), **supervision** (Hitesh Pandya, Artur Bednarczyk, Kinga Kucz, Marek Kokot, Christer Gottfridsson, Lars Pettersson, Jie Mei, Karin Skansen, Jennifer L. Bell, David Petullo, Kathryn Collison, Patrik Bondarov, Mehul Patel), **validation** (Omar S. Usmani, Fernando J. Martinez, Hitesh Pandya, Artur Bednarczyk, Marek Kokot, Christer Gottfridsson, Lars Pettersson, Jie Mei, Karin Skansen, Jennifer L. Bell, David Petullo, Mandeep Jassal, Mehul Patel), **visualisation** (Hitesh Pandya, Mandeep Jassal, Mehul Patel), **writing – reviewing and editing** (Omar S. Usmani, Fernando J. Martinez, Hitesh Pandya, Matthew Camiolo, Artur Bednarczyk, Kinga Kucz, Marek Kokot, Christer Gottfridsson, Magnus Aurivillius, Lars Pettersson, Jie Mei, Karin Skansen, Jennifer L. Bell, David Petullo, Kathryn Collison, Patrik Bondarov, Mandeep Jassal, Mehul Patel). All authors decided to submit the manuscript.

## Data sharing statement

Data underlying the findings described in this manuscript may be obtained in accordance with AstraZeneca's data sharing policy described at https://astrazenecagrouptrials.pharmacm.com/ST/Submission/Disclosure. Data for studies directly listed on Vivli can be requested through Vivli at http://www.vivli.org. Data for studies not listed on Vivli could be requested through Vivli at https://vivli.org/members/enquiries-about-studies-not-listed-on-the-vivli-platform. The AstraZeneca Vivli member page is also available outlining further details: https://vivli.org/ourmember/astrazeneca/.

## Declaration of interests

Omar S. Usmani has received personal fees from AstraZeneca, Boehringer Ingelheim, Chiesi, Cipla, Covis, Deva, GlaxoSmithKline, Kamada, Menarini, Mundipharma, Novartis, Orion, Sandoz, Takeda, Trudell Medical, and UCB; has received research grants from AstraZeneca, Boehringer Ingelheim, Chiesi, and GlaxoSmithKline; and has received consulting fees from AstraZeneca, Cipla, and Mereo Biopharma. He is also President of the International Society of Aerosols in Medicine, Chair of the UK Inhaler Group, the European Respiratory Society Council Chair elect 2024–2025, and was the Assembly 5 Head of the European Respiratory Society from 2020 to 2023. Fernando J. Martinez has consulted for AstraZeneca, Chiesi, DevPro, GlaxoSmithKline, Novartis, and Roche; has received honoraria from Sanofi/Regeneron, and UpToDate; and received payment or honoraria for lectures and presentations from AstraZeneca, GlaxoSmithKline, and Roche. Support for the study and for development of the current manuscript was provided by AstraZeneca. Jennifer L. Bell is contracted by AstraZeneca. Hitesh Pandya, Matthew Camiolo, Artur Bednarczyk, Christer Gottfridsson, Magnus Aurivillius, Lars Pettersson, Jie Mei, Karin Skansen, Kathryn Collison, Patrik Bondarov, Mandeep Jassal, and Mehul Patel are employees of AstraZeneca and hold stock and/or stock options in the company. Kinga Kucz, Marek Kokot, and David Petullo are employees of AstraZeneca.

## Appendix A Supplementary data

Redacted Protocol
