# Supplementary material for: Safety of budesonide/glycopyrronium/formoterol fumarate dihydrate delivered by HFO-1234ze versus HFA-134a in chronic obstructive pulmonary disease: a phase 3, multi-site, randomised, double-blind, parallel-group, active-comparator study
Source: eClinicalMedicine. 2025 Aug 12;87:103402. doi: 10.1016/j.eclinm.2025.103402 (PMC12359160; doi:10.1016/j.eclinm.2025.103402)
Supplement: Redacted Protocol [file mmc1.pdf]

**Clinical Study Protocol**	
Study Intervention	PT010
Study Code	D5985C00003
Version	2.0
Date	14 December 2023

A Randomized, Double-Blind, 12-Week (with an Extension to 52 Weeks in a subset of Participants), Multi-Center Study to Assess the Safety of Budesonide, Glycopyrronium, and Formoterol Fumarate (BGF) Delivered by MDI HFO Compared to BGF delivered by MDI HFA in Participants with Moderate to Very Severe Chronic Obstructive Pulmonary Disease (COPD)

Sponsor Name: AstraZeneca AB

Legal Registered Address: 151, 85 Södertälje, Sweden

Regulatory Agency Identifier Number(s)

IND Number: IND118313

EudraCT Number: 2022-001476-33

This Clinical Study Protocol has been subject to a peer review according to AstraZeneca Standard procedures. The Clinical Study Protocol is publicly registered and the results are disclosed and/or published according to the AstraZeneca Global Policy on Bioethics and in compliance with prevailing laws and regulations.

Redacted for Public Disclosure

Protocol Number: D5985C00003

Amendment Number: Amendment 1.0

Study Intervention: PT010

Study Phase: III

Short Title: A Study to Assess the Safety of Budesonide/Glycopyrronium/Formoterol Fumarate with the Hydrofluoroolefin Propellant in Participants with Moderate to Very Severe Chronic Obstructive Pulmonary Disease

International co-ordinating investigator (ICI)
Name: PPD
Center address:
PPD

Study Physician Name and Contact Information will be provided separately

SUMMARY OF CHANGES TABLE

DOCUMENT HISTORY	
**Document**	**Date**
CSP Version 2.0	14 December 2023
CSP Version 1.0	03 June 2022

CSP Version 2.0, 14 December 2023

This modification is considered to be substantial based on the criteria set forth in Article 10(a) of Directive 2001/20/EC of the European Parliament and the Council of the EU and in the EU CTR Article 2, 2 (13).

Overall Rationale for the Modification:

The CSP has been amended primarily to comply with EU CTR requirements for drug abuse and misuse handling, SAE reporting, data archiving and breach reporting, and study results submissions to trial registries. The CSP has also been revised to remove the planned clinical data lock after 12 weeks of treatment. The study will now be unblinded only once at the end of the study (after the last subject completes the 52-week treatment period). Both the 12-week analysis and the 52-week analysis will be completed after unblinding occurs at the end of the 52-week treatment period.

A summary of changes is presented below. Where applicable, the changes were also applied to the synopsis.

Summary of Changes:

List of Substantial Modifications

Section Number and Name	Description of Change	Brief Rationale
Section 1.1<br>Synopsis<br>Section 4.1<br>Overall Design	Removed interim 12-week clinical data lock.	To simplify logistics of maintaining the double blind through the 52-week treatment period. Separate blinded and unblinded study teams (at AstraZeneca and at its representatives performing data analysis) are no longer required in order to maintain the blind after the 12-week treatment period. Only one clinical data lock will occur, after the last participant completes 52 weeks of treatment. Unblinding and analysis for both 12 weeks and 52 weeks of treatment will occur at this time.

List of Non-substantial Modifications

Section Number and Name	Description of Change	Brief Rationale
Global change	Removed “treatment-emergent” as AE descriptor.	To align with global company standard for data outputs.
Section 1.3<br>Schedule of Activities<br>Section 8.2.4<br>ECGs and Holter Monitoring…	Added measures implemented to standardize ECG/Holter data acquisition and interpretation via centrally-provided device type and centralized assessment. Clarified this addition in new footnote o of Schedule of Activities.	Clarification
Section 4.1.1<br>Study Conduct Mitigation During Study Disruptions due to Cases of Civil Crisis, Natural Disaster, or Public Health Crisis	Removed interferon-gamma release assay from rescreening assessments.	Interferon-gamma release assay is not performed in this study.
Section 4.4<br>End of Study Definition	Clarified definition of the end of study according to EU and FDA requirements.	For consistency and alignment in terms of posting study results.
Section 5.1<br>Inclusion Criteria	Replaced list of acceptable contraception methods.	Aligned wording with a proposed UK local amendment requested by MHRA.
Section 6.1.1<br>Investigational Products	Added description of how investigational products will be supplied.	Global company requirement

Section Number and Name	Description of Change	Brief Rationale
Section 6.3<br>Measures to Minimize Bias: Randomization and Blinding	Added description of blinding procedures.	Addition of blinding procedures is for completeness of CSP; details of blinding and blind-breaking processes were previously provided to sites in IRT manual. No changes to the procedures have been made.
Section 6.5.2<br>Prohibited Medications<br>Section 6.5.3<br>Restricted Medications	Moved footnote describing seizure-free interval to correct table (restricted concomitant medications).	Clarification
Section 7.1<br>Discontinuation of Study Intervention<br>Section 8.2.4<br>ECGs and Holter Monitoring…	Specified that safety evaluations of changes in 12-lead ECG and eGFR should be made relative to the Visit 1 values.	Clarification
Section 8.2.5<br>Spirometry	Emphasized that 60-minute post-bronchodilator spirometry at Visit 2 should be performed (it should not be viewed as optional).	Clarification
Section 8.3.5<br>Adverse Events Based on Examinations and Tests	Added procedure for reporting abnormal findings from 12-lead ECG and from digital 12-lead Holter ECG.	Clarification
Section 8.3.8<br>Reporting of Serious Adverse Events	Specified that the reference document for definition of expectedness/listedness is the IB for both the AstraZeneca investigational product and the AstraZeneca active comparator.	Clarification
Section 8.3.10<br>Medication Error, Drug Abuse, and Drug Misuse<br>Appendix B 4<br>Medication Error	Added detailed Drug Abuse and Drug Misuse definition and examples.	Update required to comply with EU CTR.
Section 9.4.3<br>Safety Analysis<br>Section 9.4.3.1<br>Adverse Events	Changed planned presentation of AEOSIs to include exposure-adjusted incidence rate.	Clarification
Section 9.4.3.2<br>12-lead ECG	Updated definition of baseline to the mean of the last available triplicate measurements extracted from the Holter monitor up to 60 minutes prior to dosing at Visit 3.	Corrected baseline definition to align with planned analysis method as described in the SAP.

Section Number and Name	Description of Change	Brief Rationale
Section 9.4.3.3<br>Digital 12-lead Holter ECG	Replaced definition of baseline with statement that baseline will be defined in SAP.	Removed incorrect baseline definition.
Section 9.4.3.4<br>Clinical laboratory values	Clarified definition of baseline to last available value prior to dosing on day of or prior to randomization.	Clarification to increase flexibility in baseline value acquisition.
Section 9.4.3.5<br>Vital signs	Updated definition of baseline to last available value prior to dosing on day of or prior to randomization.	Corrected baseline definition to align with SAP and clarified to increase flexibility in baseline value acquisition.
Appendix A 1<br>Regulatory and Ethical Considerations	Added sub-section "Regulatory Reporting Requirements for Serious Breaches".	Update required to comply with EU CTR and global company requirement.
Appendix A 4<br>Data Protection	Added further detail relating to handling of personal data breaches.	Update required to comply with EU CTR.
Appendix A 6<br>Dissemination of Clinical Study Data	Updated information about submission of trial results summaries to registries including EU CTIS.	Update required to comply with EU CTR.
Appendix A 7<br>Data Quality Assurance	Updated information about retention timelines of records and documents (25 years after study archiving or as required by local regulations).	Update required to comply with EU CTR and global company requirement.
Appendix B 4<br>Medication Error, Drug Abuse, and Drug Misuse	Deleted inapplicable table of AE intensity grading scales not used in this study.	Clarification
Appendix D 2<br>Genetic Research Plan and Procedures	Corrected timing of genetic research blood collection to Visit 3 after randomization.	Alignment with SoA and other sections of the CSP.
Appendix F<br>Changes Related to Mitigation of Study Disruptions Due to Cases of Civil Crisis, Natural Disaster, or Public Health Crisis	Inserted hyperlinks to cross-referenced CSP sections regarding rescreening procedures.	Clarification
Appendix G	Removed "eDiary" entry from List of Abbreviations.	Clarification
Throughout	Minor editorial and document formatting revisions.	Minor, therefore have not been summarized.

TABLE OF CONTENTS

TITLE PAGE ... 1
SUMMARY OF CHANGES TABLE ... 3
TABLE OF CONTENTS ... 7
1 PROTOCOL SUMMARY ... 11
1.1 Synopsis ... 11
1.2 Schema ... 15
1.3 Schedule of Activities ... 16
2 INTRODUCTION ... 23
2.1 Study Rationale ... 23
2.2 Background ... 23
2.3 Benefit/Risk Assessment ... 24
2.3.1 Risk Assessment ... 25
2.3.2 Benefit Assessment ... 25
2.3.3 Overall Benefit: Risk Conclusion ... 25
3 OBJECTIVES AND ENDPOINTS ... 26
4 STUDY DESIGN ... 27
4.1 Overall Design ... 27
4.1.1 Study Conduct Mitigation During Study Disruptions Due to Cases of Civil Crisis, Natural Disaster, or Public Health Crisis ... 28
4.2 Scientific Rationale for Study Design ... 29
4.3 Justification for Dose ... 30
4.4 End of Study Definition ... 30
5 STUDY POPULATION ... 31
5.1 Inclusion Criteria ... 31
5.2 Exclusion Criteria ... 33
5.3 Inclusion Criteria Confirmation Prior to Randomization at Visit 3 ... 36
5.4 Lifestyle Considerations ... 36
5.4.1 Caffeine, Alcohol, and Tobacco ... 36
5.4.2 Activity ... 36
5.5 Screen Failures ... 36
6 STUDY INTERVENTION ... 38
6.1 Study Intervention(s) Administered ... 38
6.1.1 Investigational Products ... 38
6.1.1.1 HFO excipient ... 39
6.1.1.2 HFA excipient ... 39
6.1.2 Medical Devices ... 40

6.1.3 Active Pharmaceutical Ingredient (API): BGF (PT010) Triple Combination Dose ... 40
6.1.3.1 Budesonide ... 40
6.1.3.2 Glycopyrronium ... 40
6.1.3.3 Formoterol fumarate ... 40
6.2 Preparation/Handling/Storage/Accountability ... 40
6.3 Measures to Minimize Bias: Randomization and Blinding ... 41
6.4 Study Intervention Compliance ... 42
6.5 Concomitant Therapy ... 43
6.5.1 Permitted During the Course of the Study ... 43
6.5.2 Prohibited Medications ... 43
6.5.3 Restricted Medications ... 44
6.5.4 Rescue Medicine ... 45
6.5.4.1 SABA ... 45
6.6 Intervention After the End of the Study ... 45
7 DISCONTINUATION OF STUDY INTERVENTION AND PARTICIPANT DISCONTINUATION/WITHDRAWAL ... 46
7.1 Discontinuation of Study Intervention ... 46
7.1.1 Temporary Discontinuation ... 47
7.2 Participant Withdrawal from the Study ... 47
7.3 Lost to Follow Up ... 48
8 STUDY ASSESSMENTS AND PROCEDURES ... 48
8.1 Efficacy Assessments ... 49
8.1.1 Exploratory COPD Assessment Test (CAT) ... 49
8.2 Safety Assessments ... 49
8.2.1 Physical Examinations ... 49
8.2.2 Vital Signs ... 49
8.2.2.1 Weight and height ... 50
8.2.3 Smoking Status ... 50
8.2.4 Electrocardiograms and Holter Monitoring - 24-Hour Continuous Electrocardiography ... 50
8.2.5 Spirometry ... 51
8.2.6 Clinical Safety Laboratory Assessments ... 52
8.2.6.1 Hematology ... 54
8.2.6.2 Clinical chemistry ... 54
8.2.6.3 Pregnancy test ... 54
8.3 Adverse Events and Serious Adverse Events ... 54
8.3.1 Time Period and Frequency for Collecting AE and SAE Information ... 55
8.3.2 Follow-up of AEs and SAEs ... 55
8.3.3 Causality Collection ... 56
8.3.4 Adverse Events Based on Signs and Symptoms ... 56
8.3.5 Adverse Events Based on Examinations and Tests ... 57
8.3.6 Adverse Events of Special Interest ... 57

8.3.6.1 Paradoxical bronchospasm ... 58
8.3.6.2 Level of COPD severity by airflow limitation ... 58
8.3.6.3 COPD exacerbation assessment ... 58
8.3.7 Disease Under Study ... 60
8.3.8 Reporting of Serious Adverse Events ... 60
8.3.9 Pregnancy ... 61
8.3.9.1 Maternal exposure ... 61
8.3.9.2 Paternal exposure ... 62
8.3.10 Medication Error, Drug Abuse, and Drug Misuse ... 62
8.3.10.1 Timelines ... 62
8.3.10.2 Medication Error ... 62
8.3.10.3 Drug Abuse ... 62
8.3.10.4 Drug Misuse ... 62
8.3.11 Medical Device Deficiencies ... 63
8.3.11.1 Time period for detecting Medical Device Deficiencies ... 63
8.3.11.2 Follow-up of Medical Device Deficiencies ... 63
8.3.11.3 Prompt reporting of Medical Device Deficiencies to Sponsor ... 63
8.3.11.4 Regulatory reporting requirements for Medical Device Deficiencies ... 64
8.4 Overdose ... 64
8.4.1 Budesonide ... 64
8.4.2 Glycopyrronium ... 65
8.4.3 Formoterol ... 65
8.5 Optional Genomics Initiative Sample ... 65
9 STATISTICAL CONSIDERATIONS ... 66
9.1 Statistical Hypotheses ... 66
9.2 Sample Size Determination ... 66
9.3 Populations for Analyses ... 66
9.4 Statistical Analyses ... 66
9.4.1 General Considerations ... 67
9.4.2 Efficacy Analysis ... 67
9.4.2.1 Exploratory CAT assessment ... 67
9.4.3 Safety Analysis ... 67
9.4.3.1 Adverse events ... 68
9.4.3.2 12-lead ECG ... 68
9.4.3.3 Digital 12-lead Holter ECG ... 68
9.4.3.4 Clinical laboratory values ... 68
9.4.3.5 Vital signs ... 68
9.4.3.6 Spirometry measurement ... 68
9.5 Interim Analyses ... 69
9.6 Data Monitoring Committee ... 69
10 SUPPORTING DOCUMENTATION AND OPERATIONAL CONSIDERATIONS ... 70
11 REFERENCES ... 101

LIST OF FIGURES

Figure 1 Study Design 15

LIST OF TABLES

Table 1 Schedule of Activities for the 12-week and 52-week extended study........ 16
Table 2 Schedule of Activities for Procedures Related to Holter ECG, Spirometry and Study Intervention Dosing 22
Table 3 Objectives and Endpoints 26
Table 4 Investigational Products 38
Table 5 Prohibited medications 43
Table 6 Restricted medications 44
Table 7 Clinical Laboratory Tests 53
Table 8 Populations for Analysis 66

LIST OF APPENDICES

Appendix A Regulatory, Ethical, and Study Oversight Considerations 70
Appendix B Adverse Events: Definitions and Procedures for Recording, Evaluating, Follow-up, and Reporting 78
Appendix C Handling of Human Biological Samples 84
Appendix D Optional Genomics Initiative Sample 86
Appendix E Medical Device AEs, ADEs, SAEs, SADEs, USADEs and Medical Device Deficiencies: Definitions and Procedures for Recording, Evaluating, Follow-up, and Reporting in Medical Device Studies 89
Appendix F Changes Related to Mitigation of Study Disruptions Due to Cases of Civil Crisis, Natural Disaster, or Public Health Crisis 96
Appendix G Abbreviations 98

1 PROTOCOL SUMMARY

1.1 Synopsis

Protocol Title: A Randomized, Double-Blind, 12-Week (with an Extension to 52 weeks in a subset of Participants), Multi-Center Study to Assess the Safety of Budesonide, Glycopyrronium, and Formoterol Fumarate (BGF) Delivered by MDI HFO Compared to BGF delivered by MDI HFA in Participants with Moderate to Very Severe Chronic Obstructive Pulmonary Disease (COPD)

Short Title: A Study to Assess the Safety of Budesonide/Glycopyrronium/Formoterol Fumarate with the Hydrofluoroolefin Propellant in Participants with Moderate to Very Severe Chronic Obstructive Pulmonary Disease

Rationale:
A critical component of a pressurized metered-dose inhalers (MDIs) formulation is the propellant (liquified compressed gas) which facilitates actuation of the device and subsequent delivery of the pharmaceutical ingredient(s). Hydrofluoroalkanes (HFAs) were introduced into MDI applications beginning with Proventil-HFA in 1994. Prior to HFAs, hydrofluorocarbons (HFCs) (or chlorofluorocarbons [CFCs]) were used in MDIs but were replaced in order to address concerns about their ozone-depleting properties. Currently used propellant gases include HFA-134a and HFA-227ea. Since their introduction, HFA-MDIs have been safely used by millions of people, primarily for the purpose of treating respiratory diseases such as COPD.

While MDI use accounts for a very small proportion of global HFA emissions, it is desirable to replace the currently used propellant gases with more environment-friendly alternatives, without compromising patient outcomes. AstraZeneca is therefore evaluating an alternative propellant (HFO-1234ze). This propellant has a near-zero Global Warming Potential (GWP) and very low photochemical reactivity. In addition, it has very similar physical properties to HFA134a which will ensure comparable performance of the delivery of the BGF dose in the MDI product.

To assess safety and tolerability, and to continue the effective delivery of the metered BGF dose, all participants (with moderate to very severe COPD) will receive either the near-zero GWP HFO1234ze (BGF MDI HFO) 320/14.4/9.6 µg or the current standard BGF MDI hydrofluoroalkane (HFA) 320/14.4/9.6 µg. Hence the only difference between treatment arms in this study is the propellant.

Objectives and Endpoints

Objectives	Endpoints
Primary	
• To assess the safety and tolerability of BGF MDI HFO as compared to BGF MDI HFA over 12 to 52 weeks in participants with moderate to very severe COPD	• AEs (including SAEs, DAEs, AEOSIs, non-serious AEs)<br>• Digital 12-lead Holter electrocardiogram (ECG)<br>• 12-lead ECG<br>• Clinical laboratory testing<br>• Vital signs
Exploratory	
• To assess the safety and tolerability of BGF MDI HFO compared to BGF MDI HFA over 12 to 52 weeks in participants with moderate to very severe COPD	• Change from pre-dose value in FEV1 at 5, 15, 30, and 60 min post dose
• To explore the effect of BGF MDI HFO compared to BGF MDI HFA over 12 to 52 weeks on respiratory health status	• Change from baseline COPD Assessment Test (CAT) at 12 and 52 weeks

Abbreviations: AEs = adverse events; AEOSIs = adverse event of special interest; BGF = budesonide, glycopyrronium, and formoterol fumarate; CAT = COPD Assessment Test; COPD = chronic obstructive pulmonary disease; DAE = discontinuation due to AE; ECG = electrocardiogram; HFA = hydrofluoroalkane; HFO = hydrofluoroolefin; MDI = metered-dose inhaler.

Overall Design

This is a Phase 3 randomized, double-blind, 12-week (with an extension to 52 weeks in a subset of participants) study comparing the safety of BGF MDI HFO 320/14.4/9.6 µg twice daily (BID) with BGF MDI HFA 320/14.4/9.6 µg BID in participants with moderate to very severe COPD.

This study will be conducted at approximately 120 sites worldwide and will randomize approximately 542 participants.

Eligible participants are at least 40 years of age and no older than 80 years of age at Visit 1, and are required to have an established clinical history of COPD as defined by the American

Thoracic Society (ATS) and European Respiratory Society (ERS) or by locally applicable guidelines. Participants are required to have a forced expired volume in one second (FEV1)/forced vital capacity (FVC) ratio of less than 0.70, and a post-bronchodilator FEV1 ≥ 25 to < 80% predicted normal value (ie, moderate to very severe COPD) and be current or former smokers with a history of at least 10 pack-years of cigarette smoking. Participants are also required to be receiving inhaled corticosteroid/long-acting beta2-agonist (ICS/LABA), long-acting muscarinic antagonist (LAMA)/LABA or ICS/LAMA/LABA inhaled maintenance therapies.

At Visit 1, participants continue taking any current COPD maintenance medications. All participants will receive Sponsor-provided SABA, albuterol, or salbutamol for rescue use throughout the screening.

At Visit 3, participants meeting all inclusion criteria and none of the exclusion criteria will be randomized to BGF HFO 320/14.4/9.6μg or BGF HFA 320/14.4/9.6μg in a 1:1 ratio. All participants will receive Sponsor-provided albuterol/salbutamol for rescue use (Section 6.5.4).

Study interventions (during treatment period) will be orally administered as 2 inhalations BID (every morning and evening approximately 12 hours apart).

Adverse events will be collected at clinic visits and telephone contact visits.

Digital 12-lead Holter ECG will be performed at Visit 3 and Visit 6 in order to collect timely relevant ECG and cardiac rhythm data and capture potential pro-arrhythmic reactions.

Adverse events of special interest (AEOSI) in this study are respiratory events such as dysphonia, cough, dyspnea, wheezing, paradoxical bronchospasm, bronchospasm, and COPD exacerbations. If any of the AEOSI are reported, the participants will be asked about time relatedness to inhalation.

Paradoxical bronchospasm is defined as a reduction in FEV1 of > 15% from baseline (ie, the FEV1 value obtained within 30 minutes prior to study intervention administration) that occurs shortly after dosing with associated symptoms of wheezing, shortness of breath, or cough. A drop in FEV1 > 15% from baseline with associated symptoms should be reported as an AE of paradoxical bronchospasm.

Monitoring for paradoxical bronchospasm will occur at each in-clinic visit by spirometry assessments at 5- and 15-minutes post-dose. If FEV1 drops > 15% from baseline, measurements should be repeated until FEV1 values have normalized. Monitoring of FEV1 will also be done at 30 and 60 min post-dose in order to provide more robust post-dose surveillance than is possible with the 5- and 15-minutes post-dose recordings included in the paradoxical bronchospasm definition.

The study is designed to collect 52 weeks of data from at least 100 participants per arm for the extended study. Continuation of 120 randomized participants per arm in the extended study is estimated to result in at least 100 completers per arm to support extended study objectives.

Disclosure Statement: This is a multi-center, double-blind, parallel group treatment study with 2 arms.

Number of Participants:

A planned total of 542 participants will be randomized to receive study treatment. From the 271 participants enrolled in each treatment arm of the initial 12-week study, 120 participants in each arm will continue (on first-in-study basis) participation in the extended study (52-week treatment) to allow for at least 100 participants to complete 52 weeks of treatment. Participants in the extended study will remain on randomized treatment.

Note: "Enrolled" means a participant's, or their legally acceptable representative's, agreement to participate in a clinical study following completion of the informed consent process. Potential participants who are screened for the purpose of determining eligibility for the study, but are not randomly assigned/assigned in the study, are considered "screen failures", unless otherwise specified by the protocol.

Intervention Groups (12-week and 52-week):

Initial 12 weeks:

- BGF MDI HFO on Day(s) 1 through 84 (271 participants).
- BGF MDI HFA on Day(s) 1 through 84 (271 participants).

Treatment extension to 52 weeks (subset of initial participants):

- BGF MDI HFO on Days 85 through 364 (120 participants).
- BGF MDI HFA on Days 85 through 364 (120 participants).

Participants will remain on study for a minimum of 16 weeks (including the screening and treatment period and a follow-up visit), to a possible maximum of 56 weeks if the participant is included in the extended study.

Data Monitoring Committee: No

Statistical Methods

Analyses will be performed by the Sponsor or its representatives. Demographic and baseline characteristics data will be summarized by treatment. Categorical variables will be summarized using frequency and percentages, where the denominator for calculation is the underlying analysis set population unless otherwise specified. Continuous variables will be summarized with descriptive statistics using number of available observations, mean, standard deviation, median, minimum, and maximum, and quartiles where appropriate.

All AEs beginning during the treatment or follow-up period will be summarized descriptively by treatment arm, at the level of the Medical Dictionary for Regulatory Activities (MedDRA) preferred term (PT) and system organ class (SOC), for the Safety analysis set. The summaries for each treatment arm will include the number and percentage of participants experiencing the event and the number of events.

For adverse events of special interest (AEOSI) incident rates and the associated 95% confidence interval (CI) will be presented. The rate difference between treatment arms and the associated 95% CIs will also be reported. No formal hypothesis tests will be performed. Details will be provided in the statistical analysis plans.

1.2 Schema

Figure 1 Study Design

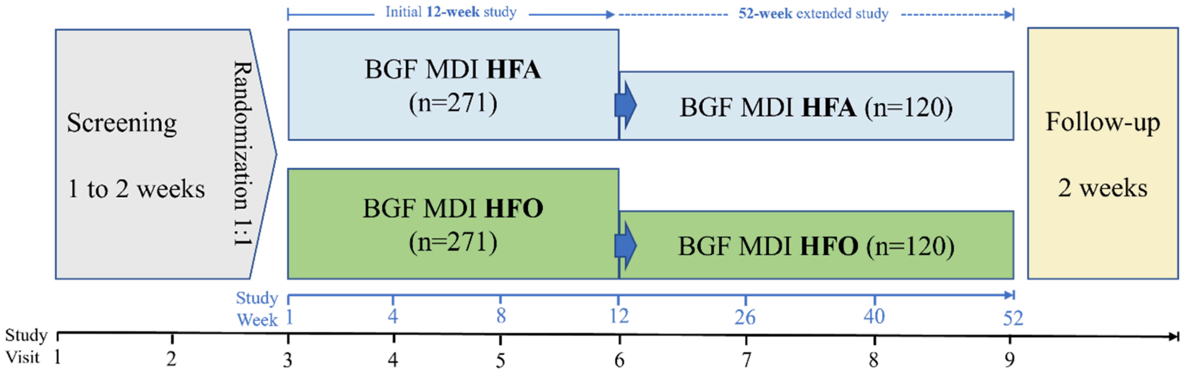

Note: Telephone contact required every 2 weeks outside of clinic visits following the start of study treatment.
Abbreviations: BGF= budesonide, glycopyrronium, and formoterol fumarate; HFA=hydrofluoroalkane; HFO=hydrofluoroolefin; MDI= metered-dose inhaler.

1.3 Schedule of Activities

Table 1 Schedule of Activities for the 12-week and 52-week extended study

**Study visit**	**1**	**2**	**3**	**4**	**5**	**6**	**Follow-up TC[a]**	**7[b]**	**8[b]**	**9[b]**	**Unsch. visit(s)[c]**	**WD visit**	**Follow-up TC[a]**	**Details in CSP Section or Appendix**
	Screening		Random -ization			12-week EOT visit				52-week EOT visit				
Study Week [d, e]	-2 to -1	-1[f]	0	4	8	12	14	26	40	52	As required	As required	54	
Study Day [g]	-14 to -7 (±2)	-7 to -1 (±2)	1	28 (±2)	56 (±5)	84 (±5)	98 (±2)	182 (±7)	280 (±7)	364 (±7)	As required	last dose (+7) [h]	378(±2)	
Informed consent	X													Appendix A 3
Inclusion/ exclusion criteria	X	X	X											Sections 5.1 and 5.2
Verify continued eligibility			X	X	X	X		X	X	X	X			Sections 5.1 and 5.2
Routine clinical procedures														
Demography and medical/surgical history	X													Sections 5.1 and 5.2
Smoking status	X	X	X	X	X	X		X	X	X	X	X		Section 8.2.3
Prior/concomitant medication review [i]	X	X	X	X	X	X		X	X	X	X	X		Section 6.5

Table 1 Schedule of Activities for the 12-week and 52-week extended study

Study visit	1	2	3	4	5	6	Follow-up TC[a]	7[b]	8[b]	9[b]	Unsch. visit(s)[c]	WD visit	Follow-up TC[a]	Details in CSP Section or Appendix
	Screening		Random -ization			12-week EOT visit				52-week EOT visit				
Study Week [d, e]	-2 to -1	-1[f]	0	4	8	12	14	26	40	52	As required	As required	54	
Study Day [g]	-14 to -7 (±2)	-7 to -1 (±2)	1	28 (±2)	56 (±5)	84 (±5)	98 (±2)	182 (±7)	280 (±7)	364 (±7)	As required	last dose (+7) [h]	378(±2)	
Spirometry	X [j]	X	X [k]	X [k]	X [k]	X [k]		X [k]	X [k]	X [k]	X [k]	X		Section 8.2.5
Reversibility to albuterol		X												Section 8.2.5
Troponin assessment			X [l]											Section 8.2.6
Routine safety measurements														
AE[m]	X	X	X	X	X	X	X	X	X	X	X	X	X	Section 8.3
TC AE follow-up[m]			X	X	X	X	X	X	X	X	X	X	X	Section 8.3.1
Pregnancy testing[n]	X	X	X	X	X	X		X	X	X		X		Section 8.3.9
Clinical safety laboratory assessment (blood)	X		X			X				X	X	X		Section 8.2.6

Table 1 Schedule of Activities for the 12-week and 52-week extended study

Study visit	1	2	3	4	5	6	Follow-up TC[a]	7[b]	8[b]	9[b]	Unsch. visit(s)[c]	WD visit	Follow-up TC[a]	Details in CSP Section or Appendix
	Screening		Random -ization			12-week EOT visit				52-week EOT visit				
Study Week [d, e]	-2 to -1	-1[f]	0	4	8	12	14	26	40	52	As required	As required	54	
Study Day [g]	-14 to -7 (±2)	-7 to -1 (±2)	1	28 (±2)	56 (±5)	84 (±5)	98 (±2)	182 (±7)	280 (±7)	364 (±7)	As required	last dose (+7) [h]	378(±2)	
Physical examination	X		X			X				X		X		Section 8.2.1
Brief physical examination		X		X	X			X	X		X			Section 8.2.1
Height and weight	X													Section 8.2.2
Vital signs	X	X	X	X	X	X		X	X	X		X		Section 8.2.2
12-Lead ECG [o]	X			X	X					X		X		Section 8.2.4
Digital 12-lead Holter ECG [o, p]			X			X								Section 8.2.4
COPD Assessment Test (CAT)	X					X				X	X	X		Section 8.1.1
Genomics initiative, optional exploratory genetic sample			X											Appendix D

Table 1 Schedule of Activities for the 12-week and 52-week extended study

Study visit	1	2	3	4	5	6	Follow-up TC[a]	7[b]	8[b]	9[b]	Unsch. visit(s)[c]	WD visit	Follow-up TC[a]	Details in CSP Section or Appendix
	Screening		Random-ization			12-week EOT visit				52-week EOT visit				
Study Week [d, e]	-2 to -1	-1[f]	0	4	8	12	14	26	40	52	As required	As required	54	
Study Day [g]	-14 to -7 (±2)	-7 to -1 (±2)	1	28 (±2)	56 (±5)	84 (±5)	98 (±2)	182 (±7)	280 (±7)	364 (±7)	As required	last dose (+7) [h]	378(±2)	
Study intervention administration														
Dispense/collect Sponsor-provided albuterol (as needed) [q]	X	X	X	X	X	X		X	X	X	X			Section 6.5.4
Discontinue COPD maintenance treatment			X											Section 6.5
Restart maintenance COPD medication after study intervention has been stopped						X [r]				X [r]		X [r]		Section 6.5
Dispense/collect blinded study intervention [s]			X	X	X	X		X	X	X	X	X		Section 6.1
Inhalation technique and cleaning training and verification		X	X	X	X	X		X	X	X	X			Section 6.1
Medical Device deficiency check			X	X	X	X		X	X	X	X			Section 8.3.11

Table 1 Schedule of Activities for the 12-week and 52-week extended study

Study visit	1	2	3	4	5	6	Follow-up TC[a]	7[b]	8[b]	9[b]	Unsch. visit(s)[c]	WD visit	Follow-up TC[a]	Details in CSP Section or Appendix
	Screening		Random -ization			12-week EOT visit				52-week EOT visit				
Study Week [d, e]	-2 to -1	-1[f]	0	4	8	12	14	26	40	52	As required	As required	54	
Study Day [g]	-14 to -7 (±2)	-7 to -1 (±2)	1	28 (±2)	56 (±5)	84 (±5)	98 (±2)	182 (±7)	280 (±7)	364 (±7)	As required	last dose (+7) [h]	378(±2)	
Administer blinded study intervention in clinic			X	X	X	X		X	X	X	X			Section 6.1

a. Follow-up TC is to be at Week 14 (Day 98±2) (applicable only for 12 weeks study participants), Week 54 (Day 378±2) or 2 weeks post last dose, depending on whether the participant completed the 12 week treatment period, 52 week treatment period, or was an early withdrawal.
b. Study Visits 7, 8 & 9 are only applicable to the 52-week extended study.
c. Assessments done at Unscheduled Visits are at the discretion of the Principal Investigator (PI).
d. Risk assessments for the SARS-CoV-2 pandemic must be made prior to every in-clinic visit in line with the Study Disruptions Mitigation Instructions.
e. Sites should call participants 1 to 2 days before each scheduled in-clinic visit to remind them of the upcoming visit and related restrictions/requirements, including withholding morning study intervention dose on the day of on-site study visit.
f. Visit 2 will be scheduled following Visit 1 based on the time to receive the participant's clinical laboratory results.
g. Site should make every effort to maintain participants within the scheduled visit windows. Participants who fall outside the visit window should be placed in the protocol-defined window at the next scheduled visit.
h. Withdrawal visit is recommended as soon as possible after last dose, but within 7 days post dose.
i. At all in-clinic visits, note time of last dose of study intervention and albuterol/salbutamol; if less than 12 hours since last dose of study intervention or 6 hours since last dose of albuterol/salbutamol, the visit should be rescheduled.
j. At Visit 1 and 2 spirometry is performed for eligibility. At Visit 1 and the Withdrawal Visit, only trough spirometry will be done.
k. FEV1 within 30 min pre-dose and at 5, 15, 30, and 60 min post-dose for safety assessments. See Table 2 for a schedule of spirometry assessments.
l. Troponin measured only at baseline (Visit 3) prior study intervention administration. Post randomization troponin should be measured according to the Investigator's decision and only when clinically indicated for potential AE diagnosis/reporting.
m. **Throughout the study AEs will be captured every 2 weeks via telephone contact and/or site visits (telephone: Weeks 2, 6, 10, 14, 16, 18, 20, 22, 24, 28, 30, 32, 34, 36, 38, 42, 44, 46, 48, 50, and 54; in-person site visits: Weeks 0, 4, 8, 12, 26, 40, and 52, as well as at any applicable Unscheduled or Withdrawal visits)**.

n. Serum/ urine pregnancy testing at Visit 1 and urine pregnancy testing at following visits to be performed for women of childbearing potential. Serum LH and FSH are additionally tested (within 21 to 28 days before Visit 3) for women < 50 years of age with amenorrhea for 12 months without an alternative medical cause. See Section 8.2.6.3
o. The same device type will be used to collect data for both the 12-lead ECG and the digital 12-lead Holter ECG (see Section 8.2.4).
p. Digital 12-lead Holter ECG will be connected the day prior to Visit 3 (randomization) and Visit 6 to establish a baseline. At Visit 3 and 6, 12-lead extractions of conventional resting ECG will be done 60 min pre-dose (before spirometry) and 20 min, 60 min (before 60 min post-dose spirometry) and 4 hr post dose (see Table 2 for a schedule of Holter ECG assessments).
q. Study-provided albuterol/salbutamol is allowed for use as needed throughout the Screening and Treatment Periods.
r. COPD maintenance treatment should be prescribed/initiated at Visit 6 for participants completing the 12-week treatment period and not continuing into the Extended treatment period and at Visit 9 for participants completing the 52-week treatment period.
s. Check puff indicator on returned MDI devices to assess study intervention dosing compliance, document results in medical records/eCRF. Report Overdose for cases confirmed by study participant when he/she inhaled more than 2 puffs of BGF MDI twice daily, in the morning and in the evening.

Abbreviations: AEs = adverse events; CAT = COPD Assessment Test; COPD = chronic obstructive pulmonary disease; CSP = Clinical Study Protocol; ECG = electrocardiogram; EOT = End-of-treatment Visit; FSH = follicle-stimulating hormone test; hr = hour(s); min = minutes; SABA = short-acting beta2-agonist; TC = telephone contact; ‘±’ = within a time period (before or after); TC = Telephone contact; Unsch. = Unscheduled Visit(s); WD = Withdrawal.

Table 2 Schedule of Activities for Procedures Related to Holter ECG, Spirometry and Study Intervention Dosing

Study visit	Pre-Visit 3	Visit 3	Pre-Visit 6	Visit 6	Spirometry at Visits 4, 5, 7, 8, 9, and Unsch.
**Study Day**	**0**	**1[b]**	**83 (±5)**	**84 (±5) [b]**	**28 (±2), 56 (±5), 182 (±7), 280 (±7), 364(±7), Unsch.**
Holter monitor hook up procedure[a]	X		X		N/A
60 min pre-dose ECG (extract from Holter)		Dose -60 min		Dose -60 min	N/A
Spirometry 30 min prior to Dose		Dose -30 min		Dose -30 min	Dose -30 min
Study intervention dosing		X		X	X
Spirometry 5 min post dosing		Dose +5 min		Dose +5 min	Dose +5 min
Spirometry 15 min post dosing		Dose +15 min		Dose +15 min	Dose +15 min
ECG +20 min post dosing - extract from 24 Hours Holter		Dose +20 min		Dose +20 min	N/A
Spirometry +30 min post dosing		Dose +30 min		Dose +30 min	Dose +30 min
ECG +60 min post dosing - extract from 24 Hours Holter[c]		Dose +60 min		Dose +60 min	N/A
Spirometry +60 min post dosing		Dose +60 min		Dose +60 min	Dose +60 min
ECG +4 hr post dosing - extract from 24 Hours Holter		Dose +4 hr		Dose +4 hr	N/A

a. Participants will need to visit the site 1 day prior to scheduled Visit 3 and Visit 6 for Holter hook up procedure, after which the participant may return home or, if justified due to travel distance and following prior approval by Sponsor, alternative lodging close to the site may be provided. The suggested time slot for both pre-visits (day before Visit 3 and day before Visit 6) is 13:00 (±2 hr).

b. Note: More than 5 hr is needed to complete assessments required by CSP/SoA, so if Visit 3/6 will start at 07:00, the earliest visit would end is 12:00; similarly for other minimal time slots 08:00 to 13:00; 09:00 to14:00, etc.

c. ECG +60 min post study intervention dosing must be done prior to Spirometry +60 min post study intervention dosing.

Abbreviations: CSP = Clinical Study Protocol; ECG = electrocardiogram; hr = hours; min = minutes; N/A = not applicable; '±' = within a time period (before or after); Unsch. = Unscheduled Visit(s).

2 INTRODUCTION

2.1 Study Rationale

The current HFA propellant (study comparator treatment) is known to have a relatively high GWP compared with some alternative propellants. The reformulation of AstraZeneca's MDI products with HFO have much lower GWP and will allow patients to continue to use MDI type treatments while contributing to AstraZeneca's sustainability efforts.

This study will evaluate the safety and tolerability of BGF MDI HFO 320/14.4/9.6 μg BID compared with BGF MDI HFA 320/14.4/9.6 μg BID in participants with moderate to very severe COPD over 12 to 52 weeks. The formulations to be evaluated in this study are MDIs containing a fixed-dose triple combination of budesonide, glycopyrronium, and formoterol fumarate, that utilize either the current propellant HFA or HFO with lower GWP. The BGF formulations only differ in the propellant excipient and are otherwise identical.

2.2 Background

Chronic obstructive pulmonary disease (COPD) is a common progressive disease with substantial associated morbidity and mortality. As many as 384 million people globally suffer from COPD (WHO 2020). Many of these patients are currently treated using MDIs containing propellants that contribute to the global carbon footprint.

The most important risk factor for COPD is tobacco smoke and other air pollutants; approximately 75% of COPD deaths are attributed to cigarette smoking, however, 1 in 4 people with COPD have never smoked (Wheaton et al 2019). Even a single COPD exacerbation may be associated with a significant increase in the rate of lung function decline and can be associated with reduction in life expectancy (Halpin et al 2017; Suissa et al 2012). Well-managed, stable COPD patients have a lower environmental impact than uncontrolled patients, who may over-use MDI reliever therapies containing older propellants or require visits to healthcare services (Usmani et al 2019; O'Byrne et al 2017).

COPD also has a potentially harmful economic impact. In 2010, COPD resulted in over 10 million office visits, nearly 1.5 million emergency department visits, 700,000 hospitalizations, and 133,575 deaths in the US (Ford et al 2013). In 2010, US total medical treatment costs attributed solely to COPD (ie, excluding comorbidities) were estimated to be $32.1 billion, with an additional $3.9 billion in COPD costs resulting from worker absenteeism (Ford et al 2015). Even in industrialized countries such as the US where anti-smoking initiatives have been relatively successful, the legacy of past smoking behavior in aging populations ensures that the COPD burden will unavoidably continue to climb over the next 20 to 30 years (Feenstra et al 2001).

Pressurized MDIs, also known as pMDIs, are one of the most common types of devices used in inhaled medicines. Inhaled medicines reduce COPD exacerbations, which are potentially life-threatening events (Sastre et al 2016 and Beasley et al 2019). Although HFA (and previously CFC) propellants have been proven to have minimal adverse effects and are currently fit-for-purpose, they contain small quantities of a type of greenhouse gas (fluorinated gases), which acts as a heat shield in the Earth's atmosphere (Pritchard 2020). They are an important device option for patients, particularly where familiarity with the device, limited lung function, young or advanced age, reduced dexterity or cognition are considerations (Schreiber et al 2020).

The next-generation MDI to treat COPD should contain a propellant that can deliver a metered dose at a similar level of safety and tolerability as the currently applied propellants (Roche et al 2016). Positive action is required to switch to an alternative propellant for MDIs with low GWP. The desire to reduce any impact to the current level of greenhouse gas emissions is a global ambition, but alternative propellants may also reduce manufacturing costs, resulting in cost benefits to the patient. An alternative mode of dose delivery is possible with dry powder inhalers (DPIs) and Mist inhalers, however, this mode of delivery is not suitable for all medical needs and are more expensive to produce (Wilkinson et al 2019).

Breztri Aerosphere® (budesonide/glycopyrronium/formoterol fumarate), a triple combination therapy for COPD, is the first medicine planned for transition to this next-generation MDI propellant. By offering both next-generation MDIs, with a near-zero GWP propellant, and DPI medicines, important therapeutic options for patients have the potential to achieve ambitious environmental targets. AstraZeneca has made a company-wide commitment to meet climate neutrality goals through reducing the impact of these propellants on the environment, however, it is important to ensure that there is no compromise to patient outcomes.

2.3 Benefit/Risk Assessment

Budesonide, glycopyrronium, and formoterol fumarate are approved in many countries worldwide in multiple formulations for different indications, such as the treatment of COPD.

This study follows from study D5985C00001, an assessment of the pharmacokinetics, safety, and tolerability of a fixed-dose combination of budesonide, glycopyrronium, and formoterol when administered as single doses in 3 different propellant formulations in healthy participants. The study results showed that systemic exposure to budesonide, glycopyrronium, and formoterol was similar for BGF MDI HFO-1234ze (a hydrofluoroolefin propellant) compared with the reference product, BGF MDI HFA-134a (a hydrofluoroalkane propellant), and HFC-152a ([CH3CHF2] an alternative propellant of low GWP).

There were no safety signals observed in Study D5985C00001 when the combination of budesonide, glycopyrronium, and formoterol were administered as single doses in 3 different

propellant formulations. A non-validated scale was used to evaluate participants' perception of the taste of the 3 treatments. There was no indication of meaningful differences between the products in this taste assessment.

In addition, risks to study participants have been minimized in the following ways:

- All participants will be screened and those with any chronic condition that could, in the judgment of the Investigator, represent a risk to safe participation, will be excluded.
- Use of Holter ECG, and reliable spirometry assessments to monitor for the onset of AEs.
- Participants will be routinely monitored for AEs during telephone contacts and clinic visits.

2.3.1 Risk Assessment

The information contained in this study is consistent with current knowledge of the risks and benefits of the study intervention; for additional information, refer to the latest product Investigator's Brochure (IB). Risks, responsibilities, and benefits are additionally detailed in the informed consent form (ICF). For a detailed discussion of the known safety profile of BGF MDI HFO, please refer to the current IB.

This global study may be initiated or conducted during the SARS-CoV-2 pandemic, another civil crisis, natural disaster, or public health crisis; the regional and/or country level risk may vary during the conduct of the study. Guidance will be ratified with local regulations, health authority and relevant professional bodies to minimize the expected direct risks to site personnel and study participants. Alternative measures and procedures may be implemented during the conduct of the study as described in Section 4.1.1. Risk assessments for the SARS-CoV-2 pandemic must be made prior to every in-clinic visit in line with the Study Disruptions Mitigation Instructions.

2.3.2 Benefit Assessment

The benefit of introducing the next-generation of MDIs, with a near-zero GWP propellant, is to maintain the patient health care standards of BGF dose delivery, and at the same time reducing the impact of older more environmentally harmful propellants.

2.3.3 Overall Benefit: Risk Conclusion

For the safety of participants, the protocol has incorporated various risk mitigation measures including appropriate inclusion and exclusion criteria and close monitoring of participants.

3 OBJECTIVES AND ENDPOINTS

The following information relates to the objectives of the study and the rationale behind their selection:

Table 3 Objectives and Endpoints

**Objectives**	**Endpoints**
Primary	
• Safety objectives: To assess the safety and tolerability of BGF MDI HFO as compared to BGF MDI HFA over 12 to 52 weeks in participants with moderate to very severe COPD	• AEs (including SAEs, DAEs, AEOSIs, non-serious AEs)<br>• Digital 12-lead Holter electrocardiogram (ECG)<br>• 12-lead ECG<br>• Clinical laboratory testing<br>• Vital signs
Exploratory	
• To assess the safety and tolerability of BGF MDI HFO compared to BGF MDI HFA over 12 to 52 weeks in participants with moderate to very severe COPD	• Change from pre-dose value in FEV1 at 5, 15, 30, and 60 min post dose
• To explore the effect of BGF MDI HFO compared to BGF MDI HFA over 12 to 52 weeks on respiratory health status	• Change from baseline COPD Assessment Test (CAT) at 12 and 52 weeks

Abbreviations: AEs = adverse events; AEOSIs = adverse event of special interest; BGF = budesonide, glycopyrronium, and formoterol fumarate; CAT = COPD Assessment Test; COPD = chronic obstructive pulmonary disease; DAE = discontinuation due to AE; ECG = electrocardiogram; MDI = metered-dose inhaler.

4 STUDY DESIGN

4.1 Overall Design

The formulations to be evaluated in this study are MDIs containing a fixed-dose triple combination of BGF that utilize either the current propellant HFA or HFO with lower GWP. The BGF formulations only differ in the propellant excipient and are otherwise identical.

This is a Phase 3 randomized, double-blind, 12-week (with extension to 52 weeks in a subset of participants) study comparing the safety of BGF MDI HFO 320/14.4/9.6 μg BID with BGF MDI HFA 320/14.4/9.6 μg BID in participants with moderate to very severe COPD.

For an overview of the study design see Figure 1. For details on treatments given during the study, see Section 6.1.

This study will be conducted at approximately 120 sites worldwide and will randomize approximately 542 participants.

Eligible participants are at least 40 years of age and no older than 80 years of age and are required to have an established clinical history of COPD as defined by the American Thoracic Society (ATS)/European Respiratory Society (ERS) (Celli et al 2004, Louis et al 2022). Participants are required to have an FEV1/FVC ratio of <0.70 and a post-bronchodilator FEV1 ≥25 to <80% predicted normal value (ie, moderate to very severe COPD) and be current or former smokers with a history of at least 10 pack-years of cigarette smoking. Participants are also required to be receiving ICS/LABA, LAMA/LABA or ICS/LAMA/LABA inhaled maintenance therapies.

At Visit 1, participants continue taking any current COPD maintenance medications. All participants will receive Sponsor-provided SABA consisting of albuterol or salbutamol for rescue use throughout the screening (refer to Section 6.5.4).

At Visit 3, participants meeting all inclusion criteria and none of the exclusion criteria will be randomized to BGF HFO 320/14.4/9.6 μg or BGF HFA 320/14.4/9.6 μg in a 1:1 ratio. All participants will receive Sponsor-provided albuterol/salbutamol for rescue use.

Study interventions (during treatment period) will be orally administered as 2 inhalations BID (every morning and evening approximately 12 hours apart).

Adverse events will be collected at clinic visits and telephone contact visits throughout the study.

Digital 12-lead Holter ECG will be performed at Visit 3 and Visit 6 in order to collect timely relevant ECG and cardiac rhythm data and capture potential pro-arrhythmic reactions.

The AEOSIs in this study are respiratory events such as dysphonia, cough, dyspnea, wheezing, paradoxical bronchospasm, bronchospasm, and COPD exacerbations. If any of the AEOSI are reported, the participants will be asked about time relatedness to inhalation.

Paradoxical bronchospasm is defined as a reduction in FEV1 of > 15% from baseline (ie, the FEV1 value obtained within 30 minutes prior to study intervention administration) that occurs shortly after dosing with associated symptoms of wheezing, shortness of breath, or cough. For the purpose of this study, a drop in FEV1 > 15% from baseline at 5 and/or 15 minutes post-dose, with associated respiratory symptoms should be reported as an AE of paradoxical bronchospasm.

Monitoring for paradoxical bronchospasm will occur at each in-clinic visit by spirometry assessments at 5- and 15-minutes post-dose. If FEV1 drops > 15% from baseline, measurements should be repeated until FEV1 values have normalized. Monitoring of FEV1 will also be done at 30 and 60 min post-dose in order to provide more robust post-dose surveillance than is possible with the 5- and 15-minutes post-dose recordings including in the paradoxical bronchospasm definition.

The study is designed to collect 52 weeks of data from approximately 100 participants per arm for the extended study. Continuation of 120 randomized participants per arm in the extended study is estimated to result in approximately 100 completers per arm to support the extended study objectives.

4.1.1 Study Conduct Mitigation During Study Disruptions Due to Cases of Civil Crisis, Natural Disaster, or Public Health Crisis

The guidance given below supersedes instructions provided elsewhere in this Clinical Study Protocol (CSP) and should be implemented only during cases of civil crisis, natural disaster, or public health crisis (eg, during quarantines and resulting site closures, regional travel restrictions, and considerations if site personnel or study participants become infected with SARS-CoV-2 or similar pandemic infection) which would prevent the conduct of study-related activities at study sites, thereby compromising the study site staff or the participant’s ability to conduct the study. The Investigator or designee should contact the Sponsor/CRO to discuss whether the mitigation plans below should be implemented.

To ensure continuity of the clinical study during a civil crisis, natural disaster, or public health crisis, changes may be implemented to ensure the safety of study participants, maintain compliance with Good Clinical Practice (GCP), and minimize risks to study integrity.

Where allowable by local health authorities, ethics committees, healthcare provider guidelines (eg, hospital policies) or local government, these changes may include the following options:

Obtaining reconsent for the mitigation procedures
(Note: in the case of verbal consent/reconsent, the ICF should be signed at the participant's next contact with the study site).

Rescreening: Additional rescreening for screen failure and to confirm eligibility to participate in the clinical study may be performed in previously screened participants. The Investigator should confirm this with the Sponsor/CRO's designated Medical Monitor. If rescreening takes place within 30 days after the initial screen, participants must have COVID-19 and serum pregnancy tests repeated. If rescreening takes place more than 30 days after the initial screen, all screening eligibility assessments must be redone. Participants with a positive reverse transcription polymerase chain reaction test (RT-PCR) for COVID-19 at screening may be rescreened after 12 weeks (3 months) provided that there is no development of severe COVID-19 infection or sequalae.

Home or remote visit: Performed by a site qualified Health Care Professional (HCP) or HCP provided by a third-party vendor (TPV).

Telemedicine visit: Remote contact with the participants using telecommunications technology including telephone contacts, virtual or video visits, and mobile health devices.

Home intervention supplies in case on-site visit is not possible due to COVID-19 site issues/logistics. Visit can be done remotely as TC or Video call.

For further details on study conduct during civil crisis, natural disaster, or public health crisis, refer to Appendix F.

4.2 Scientific Rationale for Study Design

The suitability of HFO-1234ze as an alternative propellant to HFA134a in the BGF MDI 320/14.4/9.6 µg formulation will be assessed in this clinical study. All other formulation components will remain the same. The components of the container closure system (MDI) used to deliver the formulation to the participant will also remain unchanged. The drug product formulation containing HFO1234ze should be stored according to the instructions on the label and the instruction for use (IFU).

All completed non-clinical studies conducted to date were designed to evaluate the toxicology (including safety pharmacology) and are described below. Unless otherwise stated, all studies were conducted via inhalation exposure. The preclinical species used (mouse, rat, rabbit, and dog) were chosen as they are recognized as appropriate non-clinical species to predict toxicological change in man and are acceptable to regulatory agencies. Due to absence of pharmacological activity of HFO-1234ze (as with existing propellant gases), limited pharmacokinetic or metabolism studies have been conducted.

4.3 Justification for Dose

The BGF dose approved for the treatment of COPD, is 2 inhalations twice daily (320/14.4/9.6 µg [BID]). Each dose of the propellant to which participants will be exposed, will thus reflect a 12-hour cycle which will be part of the 2 inhalations twice daily dosage of BGF.

In this clinical study, participants will receive BGF treatment including either the HFO or HFA propellant gas. Participants will receive 2 BGF inhalations twice daily, including an emitted dose of 58.6 mg HFO-1234ze/actuation, or 1.95 mg/kg twice daily, assuming a 60 kg body weight.

No-Observable-Effect-Levels (NOELs) and/or No-Observable-Adverse-Effect-Levels (NOAELs) identified in repeat dose and reproductive toxicology studies have shown safety margins at > 1000 times a dose of 1.95 mg/kg BID.

Based on data for HFA-134a (a structurally related gas currently used as a propellant in MDIs), HFO-1234ze would be expected to be cleared from the circulation very rapidly following administration.

Preclinical exposure data with HFA-134a showed an estimated half-life of no more than 7 minutes in preclinical studies (Alexander et al 1995).

4.4 End of Study Definition

For the purpose of Clinical Trial Transparency, the definition of the end of the study differs under FDA and EU regulatory requirements:

European Union requirements define study completion as the last visit of the last subject for any protocol-related activity.

Food and Drug Administration requirements define two completion dates:

Primary Completion Date – the date that the final participant is examined or receives an intervention for the purposes of final collection of data for the primary outcome measure, whether the clinical study concluded according to the pre-specified protocol or was terminated. In the case of clinical studies with more than one primary outcome measure with different completion dates, this term refers to the date on which data collection is completed for all of the primary outcomes.

Study Completion Date – the date the final participant is examined or receives an intervention for the purposes of final collection of data for the primary and secondary outcome measures and AEs (for example, the last participant's last visit), whether the clinical study concludes according to the pre-specified protocol or is terminated.

A participant is considered to have completed this study if they have completed all phases of the study including the last scheduled procedure shown in the SoA (Table 1).

The study will end when the last remaining participant from approximately 100 participants per treatment arm completes his/her Week 52/Visit 9 and subsequent 2-week follow-up telephone contact. If study intervention is discontinued prior to the Week 52/Visit 9, then study will end at the completion of the Withdrawal Visit and subsequent 2-week follow-up telephone contact.

Participants contributing only to the 12 weeks safety study will need to complete their Week 12/Visit 6 and subsequent 2-week follow-up telephone contact. If study intervention was discontinued prior to the Week 12/Visit 6, then study will end at the completion of the Withdrawal Visit and subsequent 2-week follow-up telephone contact.

5 STUDY POPULATION

The PI is to maintain a screening log of all potential participants who consent and are subjected to screening procedures. Participants who fail to meet the inclusion criteria or meet any of the exclusion criterion are refused entry into the study. There are no exceptions to this rule. One repeat assessment can be requested at the discretion of the PI (including any laboratory tests, ECG measurements or vital sign measurements) at the screening visit and on admission to the study center.

5.1 Inclusion Criteria

Participants are eligible to be included in the study only if all of the following criteria apply:

Age

1. Participant must be 40 to 80 years of age inclusive, at the time of signing the ICF;

Type of Participant and Disease Characteristics

2. Participants who have a documented history of physician-diagnosed COPD as defined by the ATS/ERS (Celli et al 2004) or by locally applicable guidelines;
3. Participants who have been regularly using dual ICS/LABA, LAMA/LABA, or ICS/LAMA/LABA (open or fixed-dose combinations) inhaled maintenance therapies for the management of their COPD for at least 6 weeks prior to Screening;
4. Participants who have pre-bronchodilator FEV1 of $<$ 80% predicted normal at Visit 1;
5. Participants who have post-bronchodilator FEV1/FVC ratio of $<$ 0.70 and post-bronchodilator FEV1 of $\geq$ 25% to $<$ 80% predicted normal at Visit 2;
6. Participants who have CAT score $\geq$ 10 at Visit 1;
7. Participants who are current/former smokers with a history of at least 10 pack-years of tobacco smoking (1 pack year = 20 cigarettes smoked per day for 1 year);

8. Participants who are willing and, in the opinion of the Investigator, able to adjust current COPD therapy, as required by the protocol;
9. Participants must be able to demonstrate acceptable MDI administration and spirometry technique;
10. Participants who are willing to remain at the study center as required per protocol to complete all visit assessments;

Sex

11. Females must either be not of childbearing potential, or using a form of highly effective birth control as defined below:
    - Women not of childbearing potential are defined as women who are either permanently sterilized (hysterectomy, bilateral oophorectomy, or bilateral salpingectomy), or who are postmenopausal. Women will be considered postmenopausal if they have been amenorrhoeic for 52 weeks (12 months) prior to the planned date of randomization without an alternative medical cause. The following age-specific requirements apply:
    - Women < 50 years old would be considered postmenopausal if they have been amenorrhoeic for 52 weeks (12 months) or more following cessation of exogenous hormonal treatment and follicle stimulating hormone levels in the postmenopausal range.
    - Women ≥ 50 years old would be considered postmenopausal if they have been amenorrhoeic for 52 weeks (12 months) or more following cessation of all exogenous hormonal treatment.
12. Female participants of childbearing potential must use one highly effective form of birth control. A highly effective method of contraception is defined as one that can achieve a failure rate of less than 1% per year when used consistently and correctly. At enrollment, women of childbearing potential who are sexually active with a non-sterilized male partner should be stable on their chosen method of highly effective birth control, as defined below, and willing to remain on the birth control until at least 14 days after last dose of study intervention. Cessation of contraception after this point should be discussed with a responsible physician. Periodic abstinence (calendar, symptothermal, post-ovulation methods), withdrawal (coitus interruptus), spermicides only, and lactational amenorrhea method are not acceptable methods of contraception. Female condom and male condom should not be used together.
    - All women of childbearing potential must have a negative serum pregnancy test result at Visit 1
    - Women <50 years of age with amenorrhea for 12 months without an alternative medical cause must have a serum LH and FSH test (within 21-28 days before Visit 3) for study eligibility

  Highly effective birth control methods are listed below:

- Sexual abstinence defined as complete abstinence from heterosexual intercourse during the entire period of risk associated with the study treatments. The reliability of sexual abstinence needs to be evaluated in relation to the duration of the clinical trial and the preferred and usual lifestyle of the participant.
- Combined (estrogen and progestogen containing) hormonal contraception associated with inhibition of ovulation:
    - Oral
    - Intravaginal
    - Transdermal
- Progestogen-only hormonal contraception associated with inhibition of ovulation:
    - Oral
    - Injectable
    - Implantable
- Intrauterine device or intrauterine hormone-releasing system
- Male partner sterilization/vasectomy with documentation of azoospermia prior to the female participant's entry into the study, and this male is the sole partner for that participant. The documentation on male sterility can come from the site personnel's review of participant's medical records, medical examination and/or semen analysis or medical history interview provided by her or her partner.
- Bilateral tubal ligation

Informed Consent

13. Capable of giving signed informed consent as described in Appendix A which includes compliance with the requirements and restrictions listed in the ICF and in this protocol.

5.2 Exclusion Criteria

Participants are excluded from the study if any of the following criteria apply:

Medical Conditions

1. Participants who have a documented history of physician-diagnosed asthma in the opinion of the Investigator based on thorough review of medical history and medical records, within 5 years of Visit 1;
2. Participants who have COPD due to α1-Antitrypsin Deficiency;
3. Participants with historical or current evidence of a clinically significant disease including, but not limited to: cardiovascular, hepatic, renal, hematological, neurological,

endocrine, gastrointestinal, or pulmonary. Significant is defined as any uncontrolled disease or any disease that, in the opinion of the Investigator, would put the safety of the participant at risk through participation, or that could affect the efficacy or safety analyses;

4. Sleep apnea that, in the opinion of the Investigator, cannot be controlled;
5. Other respiratory disorders including known active tuberculosis, lung cancer, cystic fibrosis, significant bronchiectasis (high resolution CT evidence of bronchiectasis that causes repeated acute exacerbations), immune deficiency disorders, severe neurological disorders affecting control of the upper airway, sarcoidosis, idiopathic interstitial pulmonary fibrosis, primary pulmonary hypertension, or pulmonary thromboembolic disease;
6. Participant with moderate or severe COPD exacerbation or respiratory infection ending within 4 weeks prior to Visit 1 or during the Screening period;
7. Participant who has had a SARS-CoV-2 infection in the 8 weeks prior to Visit 1 or during the Screening Period or that required hospitalization at any time prior to Visit 1 or during the Screening Period;
8. Pulmonary resection or lung volume reduction surgery during the 26 weeks (6 months) prior to Visit 1 (ie, lobectomy, bronchoscopy lung volume reduction [endobronchial blockers, airway bypass, endobronchial valves, thermal vapor ablation, biological sealants, and airway implants]);
9. Long-term oxygen therapy;
10. Imminent life-threatening COPD (eg, need for mechanical ventilation);
11. Participant who has significant or unstable ischemic heart disease, arrhythmia, cardiomyopathy, heart failure, uncontrolled hypertension as defined by the Investigator, or any other relevant cardiovascular disorder as judged by the Investigator;
12. Participant with narrow angle glaucoma not adequately treated and/or change in vision that may be relevant, in the opinion of the Investigator;
    Note: All medications approved for control of intraocular pressures are allowed including topical ophthalmic nonselective beta-blockers and prostaglandin analogs.
13. Symptomatic prostatic hypertrophy or bladder neck obstruction/urinary retention that, in the opinion of the Investigator, is clinically significant;
    Note: Participants with trans-urethral resection of prostate or full resection of the prostate within 26 weeks (6 months) prior to Visit 1 are excluded from the study
14. Unresectable cancer that has not been in complete remission for at least 5 years prior to Visit 1;
    Note: Squamous cell and basal cell carcinomas of the skin are not exclusionary

Prior/Concomitant Therapy

15. Known history of drug or alcohol abuse within 52 weeks (12 months) of Visit 1;

16. Unable to withhold short-acting bronchodilators for 6 hours prior to lung function testing at each applicable study visit;
17. Participant is unable to abstain from protocol-defined prohibited medications during Screening and Treatment Periods;
18. Using any herbal products either by inhalation or nebulizer within 2 weeks of Visit 1 and does not agree to stop for the duration of the study;
19. Participants with a known hypersensitivity to beta2-agonists, muscarinic antagonists, or corticosteroids, or any component of the MDI;

Prior/Concurrent Clinical Study Experience

20. Participation in another clinical study with an intervention administered in the last 30 days or 5 half-lives, whichever is longer;
21. Previous randomization in any study using BGF MDI HFO (budesonide/glycopyrronium/formoterol fumarate – HFO);

Diagnostic Assessments

22. Participants with calculated eGFR ≤ 30 mL/minute/1.73m^2 using the Chronic Kidney Disease Epidemiology Collaboration (CKD-EPI) formula;
23. Any clinically relevant abnormal findings in physical examination, clinical chemistry, hematology, vital signs, or ECG, which in the opinion of the Investigator, may put the participant at risk because of his/her participation in the study;
    Note: Participants with ECG QTcF interval (corrected for heart rate using Fridericia's formula [QTcF]) > 480 msec will be excluded. Participants with high degree atrioventricular block II or III, or with sinus node dysfunction with clinically significant pauses who are not treated with pacemaker will also be excluded.

Other Exclusions

24. Planned hospitalization during the study;
25. Involvement in the planning and/or conduct of the study (applies to both AstraZeneca staff and/or staff at the study site);
26. Study Investigators, sub-Investigators, coordinators, and their employee or immediate family members;
27. Judgment by the Investigator that the participant is unlikely to comply with study procedures, restrictions and requirements;
28. For women only – currently pregnant (confirmed with positive pregnancy test), breast feeding, or planned pregnancy during the study or women of childbearing potential not using acceptable contraception measures (see Inclusion criterion 12 in Section 5.1).

5.3 Inclusion Criteria Confirmation Prior to Randomization at Visit 3

In order to be randomized, participants must continue to meet inclusion criteria for randomization at Visit 3.

5.4 Lifestyle Considerations

Participants must follow the contraception requirements outlined in Inclusion criterion 12. Restrictions relating to concomitant medications are described in Section 6.5.

5.4.1 Caffeine, Alcohol, and Tobacco

- Participants should not smoke one hour prior to lung function assessments on clinic visit days.
- Participants will abstain from ingesting caffeine- or xanthine-containing products (eg, coffee, tea, cola drinks, and chocolate) for 6 hours before and for the duration of each in-clinic study visit.

5.4.2 Activity

- Participants will abstain from strenuous exercise during Holter ECG collection for at least 30 min prior to ECG and lung function assessments at site.

5.5 Screen Failures

Screen failures are defined as participants who consent to participate in the clinical study but are not subsequently randomly assigned to study intervention. A minimal set of screen failure information is required to ensure transparent reporting of screen failure participants to meet the Consolidated Standards of Reporting Trials (CONSORT) publishing requirements and to respond to queries from regulatory authorities. Minimal information includes demography, screen failure details, eligibility criteria, and any serious AE (SAE).

Individuals who do not meet the criteria for participation in this study (screen failure) may be rescreened. Only a single rescreening is allowed in the study. Rescreened participants should be assigned the same participant number as for the initial screening.

In the following situations, the participant must be screen failed:

- Participants who experience moderate or severe COPD exacerbation or respiratory infection during screening period. They may be rescreened but no sooner than 28 days after the last day of antibiotic or systemic corticosteroids treatment or the last date of hospitalization, whatever occurred later
(Exclusion criterion 6).
- Participants who had SARS-CoV-2 infection (positive nasopharyngeal test) or that required hospitalization at Visit 1 or during screening should be screen failed. They may

be rescreened once recovered from the infection, upon approval of the AZ Study Physician (Exclusion criterion 7).

Rescreening of a participant for any reason will be allowed only upon approval of the AstraZeneca Study Physician. A documented approval for rescreening should be filed in the Investigator Study File.

6 STUDY INTERVENTION

Study intervention is defined as any investigational intervention(s), marketed product(s) or placebo intended to be administered to or medical device(s) utilized by a study participant according to the study protocol.

Placebo MDI devices with HFA will be provided for training purposes.

6.1 Study Intervention(s) Administered

6.1.1 Investigational Products

Table 4 Investigational Products

**Arm name**	BGF MDI HFO	BGF MDI HFA	Albuterol/salbutamol
**Intervention name**	Budesonide/ Glycopyrronium/ Formoterol fumarate pressurized inhalation suspension, HFO	Budesonide/ Glycopyrronium/ Formoterol fumarate pressurized inhalation suspension, HFA	Albuterol sulfate inhalation aerosol (US)/ Salbutamol sulfate inhalation aerosol (EU/RoW)
**Type**	Combination product	Combination product	Combination product
**Dose formulation**	Inhaler	Inhaler	Inhaler
**Unit dose strength(s) (Delivered dose)**	160/7.2/4.8 µg per actuation	160/7.2/4.8 µg per actuation	90 µg per actuation (US) 100 µg per actuation (EU/RoW)
**Dosage level(s)**	2 inhalations BID	2 inhalations BID	Rescue: Variable (2 inhalations as needed)<br>Reversibility: as directed for reversibility testing at Visit 2
**Route of administration**	Oral inhalation	Oral inhalation	Oral inhalation
**Use**	Experimental	Comparator	Rescue medication
**IMP and NIMP**	IMP	IMP	NIMP
**Sourcing**	Provided centrally by the Sponsor	Provided centrally by the Sponsor	Provided locally by the study site, subsidiary, or designee
**Packaging and labeling**	Study Intervention will be provided in MDI. Each MDI will be	Study Intervention will be provided in MDI. Each MDI will be	N/A

	labeled as required per country requirement	labeled as required per country requirement	

Abbreviations: BGF= budesonide, glycopyrronium, and formoterol fumarate; BID= twice daily (‘*bis in die*’); HFA=hydrofluoroalkane; HFO=hydrofluoroolefin; IMP= Investigational Product; MDI= metered-dose inhaler; N/A= Not applicable; NIMP=Non-Investigational Medicinal Product.

The study interventions (BGF MDI HFO and BGF MDI HFA) are each formulated as a suspension with micronized budesonide, micronized glycopyrronium bromide, and micronized formoterol fumarate crystals co-suspended with spray-dried porous particles in the respective propellant (HFO or HFA). The spray-dried porous particles are composed of distearoylphosphatidylcholine and calcium chloride. Each formulation is contained within a coated aluminum canister fitted with a metering valve, desiccated flow path collar, and plastic actuator. The canister has an attached dose indicator which indicates how many inhalations remain. The products are individually foil overwrapped with desiccant and are formulated with sufficient suspension to ensure delivery of 120 inhalations from the nominal 50-µL valve over the product shelf life. The products contain an additional 10 inhalations to allow for device standard maintenance procedures as described in the IFU.

Dosing instructions and dispensing details will be provided by AstraZeneca.

Details of the batch numbers will be included in the trial master file and the final CSR.

6.1.1.1 HFO excipient

HFO refrigerants are unsaturated HFC refrigerants and widely recognized as the next generation of refrigerants because of their environmental friendliness, cost-effectiveness, and greater energy efficiencies. Common synonyms include HFO-1234ze and R-1234ze(E). HFO-1234ze is also known as trans-1,3,3,3-tetrafluoropropene, tetrafluoropropylene and 1,3,3,3-tetrafluoro-1 propene.

Refer to the latest IB for more information.

6.1.1.2 HFA excipient

HFA-134a is a widely used propellant used in MDI inhalation products. Laboratory studies for up to 26 weeks (6 months) did not show any clinically meaningful differences, compared to placebo, in toxicology or carcinogenic effects.

The lack of adverse findings supports the safety of this excipients. HFA-134a is not inert as the now redundant chlorofluorocarbons, as it acts as a smooth muscle relaxant, partial pressure effects in the lungs and inhaled, and has anesthetic effects (Sellers 2017).

Refer to the latest IB for more information.

6.1.2 Medical Devices

1. The medical device constituent of the combination products to be used in this study will be an MDI (Approved).
2. Instructions for medical device use are provided in the Investigator Brochure and Combination Products section of the Summary of Medical Product Characteristics.
3. All device constituent deficiencies (including malfunction, use error and inadequate labeling) shall be documented and reported by the Investigator throughout the clinical investigation (see Section 8.3.11) and appropriately managed by the Sponsor.

6.1.3 Active Pharmaceutical Ingredient (API): BGF (PT010) Triple Combination Dose

6.1.3.1 Budesonide

Budesonide is a well-established corticosteroid approved as monotherapy and in combination worldwide in both intranasal and orally inhaled formulations for different indications, including COPD.

6.1.3.2 Glycopyrronium

Glycopyrronium (the active moiety of glycopyrronium bromide, also referred to as glycopyrrolate) is a LAMA, which exerts its bronchodilatory effect via muscarinic receptors located on smooth muscle cells within the trachea and bronchi. Glycopyrronium is approved in many countries in multiple formulations for different indications, including COPD.

6.1.3.3 Formoterol fumarate

Formoterol fumarate is a potent and selective LABA and is approved in the US and worldwide in combination with budesonide as well as with glycopyrronium for use in patients with COPD. When inhaled, formoterol fumarate acts locally in the lung as a bronchodilator. Formoterol fumarate stimulates β_2 adrenoreceptors in the airways, inducing airway smooth muscle relaxation and reducing or preventing bronchoconstriction.

Although formoterol fumarate is classified as a LABA, it has a rapid onset of action similar to SABAs. Formoterol fumarate is highly potent, displays high intrinsic activity, and can result in greater than 80% relaxation even under induced tone (Naline et al 2007). Studies in patients with COPD have demonstrated that the onset of action with formoterol fumarate is faster than with anticholinergic agents or salmeterol and similar to that of SABAs, such as albuterol, and that the duration of action is ≥ 12 hours (Berger et al 2008). Five large, placebo-controlled clinical studies of up to 52 weeks (12 months) in duration in nearly 2500 patients demonstrated that formoterol fumarate is effective and well tolerated in patients with COPD (Berger et al 2008).

6.2 Preparation/Handling/Storage/Accountability

- The Investigator or designee must confirm appropriate temperature conditions have been maintained during transit for all study intervention received and any discrepancies are reported and resolved before use of the study intervention.
- Only participants enrolled in the study may receive study intervention and only authorized site staff may supply or administer study intervention. All study intervention must be stored in a secure, environmentally controlled, and monitored (manual or automated) area in accordance with the labeled storage conditions with access limited to the Investigator and authorized site staff.
- The Investigator, institution, or the head of the medical institution (where applicable) is responsible for study intervention accountability, reconciliation, and record maintenance (ie, receipt, reconciliation, and final disposition records).
- Further guidance and information for the final disposition of unused study interventions are provided in the IP handling instruction.

6.3 Measures to Minimize Bias: Randomization and Blinding

All participants in this international multi-center study will be centrally assigned to randomized study intervention using an Interactive Response Technology/Randomization and Trial Supply Management (IRT/RTSM). Before the study is initiated, user guides, the log-in information, and directions of the IRT will be provided to each study site. Randomization will be stratified by region (Americas, Europe) and COPD disease severity (percent predicted FEV1 ≥ 50%, percent predicted FEV1 < 50%). The IRT/RTSM will provide to the Investigator(s) or pharmacists the kit identification number to be allocated to the participant at the dispensing visit. Routines for this will be described in the IRT/RTSM user manual that will be provided to each center.

This study is double-blinded with regard to treatment; ie, the Sponsor, the Investigator, all clinical staff involved in the clinical study, the participants, and the Study Monitor will remain blinded, unless safety concerns or a regulatory requirement necessitate unblinding.

The Supply Chain Study Manager, who is unblinded to manage drug supply effectively and perform transactions that take effect immediately, may have access to the RTSM system. Neither the Supply Chain Study Manager nor the IRT Delivery Team Lead will be granted access to the EDC system.

The randomization code should not be broken except in medical emergencies when the appropriate management of the participant requires knowledge of the treatment randomization.

AstraZeneca retains the right to break the code for SAEs that are unexpected and are suspected to be causally related to an investigational product and that potentially require

expedited reporting to regulatory authorities. A copy of the report, identifying the participant's intervention assignment, may be sent to Investigators in accordance with local regulations and/or Sponsor policy. Randomization codes will not be broken for the planned analyses of data until all decisions on the evaluability of the data from each individual participant have been made and documented.

The IRT will be programmed with blind-breaking instructions. In case of an emergency, in which the knowledge of the specific blinded study intervention will affect the immediate management of the participant's condition (eg, antidote available), the Investigator has the sole responsibility for determining if unblinding of a participant's intervention assignment is warranted. The participant's safety must always be the first consideration in making such a determination. If a participant's intervention assignment is unblinded, the Sponsor must be notified within 24 hours after breaking the blind. The Investigator documents and reports the action to AstraZeneca, without revealing the treatment given to participant to the AstraZeneca staff.

Where a participant does not meet all the eligibility criteria but incorrectly received study intervention, the Investigator should inform the study Sponsor immediately, and a discussion should occur between the Study Physician and the Investigator regarding whether to continue or discontinue the participant.

Returned study intervention should not be re-dispensed to the participants.

6.4 Study Intervention Compliance

Study participants will be trained by authorized site personnel on how to use MDI device and self-administer the study treatment.

When participants are dosed at the site, they will self-administer and inhale study intervention under medical supervision from the Investigator or designee.

The date, and time of dose administered during on-site visit will be recorded in the source documents and in the electronic Case Report Form (eCRF).

Compliance with at home study intervention self-administration will be assessed at each on-site visit.

Authorized site staff representative will assess study intervention compliance by reviewing puff indicators (number of remaining doses) on returned MDI devices. The puff indicator reading should be documented in the source documents and in the eCRF to allow to monitor study intervention compliance and for reconciliation.

The number of MDI devices dispensed and used/returned will be recorded at each on-site visit.

Study intervention start date/time and stop date/time will also be recorded in the eCRF.

6.5 Concomitant Therapy

6.5.1 Permitted During the Course of the Study

Participant's may continue current maintenance treatment for COPD, during the screening/run-in period, Visit 1 to Visit 3.

- SABA - Sponsor provided to be used as needed during study conduct from Visit 1 to Visit 9

6.5.2 Prohibited Medications

A list of prohibited medications is provided in Table 5.

Table 5 Prohibited medications

**COPD Medications - Minimum Required Washout Periods Prior to Lung Function Assessment at Visit 2 to Visit 3**	
SABA	6 hours[a]
SAMA	6 hours
LABA (mono and in any combination)	12 hours (24 hours for once daily medications)
LAMA (mono and in any combination)	12 hours (24 hours for once daily medications)
**COPD Medications - Minimum Required Washout Periods Prior to Study Intervention Administration at Visit 4 to Visit 9**	
SABA	6 hours[a]
Study Medication	12 hours
**COPD medications prohibited prior Visit 1 and throughout the study**	
Combinations of SAMA/SABA	2 weeks
Oral beta-agonists	2 weeks
Xanthine derivatives	2 weeks
Maintenance/prophylactic treatment with oral, systemic, or depot corticosteroids[b]	26 weeks (6 months)
Maintenance/prophylactic treatment with antibiotics[c]	26 weeks (6 months)
Leukotriene antagonists/modifiers (eg, zafirlukast, montelukast, Zileuton®)	7 days
**Prohibited Non-COPD and Non-Respiratory Medications [d] prior to Visit 1 and throughout the study**	
Intranasal ipratropium bromide	7 days

Any drug with potential to significantly prolong the QT interval [e]	14 days or 5 half-lives, whichever is longer
Other investigation drugs	30 days or 5 half-lives, whichever is longer
Nonselective beta-blocking agents (except carvedilol)	7 days
Monoamine oxidase inhibitors	14 days
Monoclonal antibodies [f]	30 days or 5 half-lives, whichever is longer
Systemic treatment with strong CYP3A4-inhibitors (eg, ketoconazole, itraconazole, ritonavir)	30 days
Systemic anticholinergics [g]	7 days
Maintenance/prophylactic treatment with oral, systemic, or depot corticosteroids	12 months
Herbal remedies for the treatment of allergic, inflammatory, or respiratory disease (eg, Chinese complementary and alternative bronchodilatory medications)	14 days
Live attenuated vaccines	30 days
COVID-19 vaccines (any dose from regimen)	7 days prior to randomization and any spirometry assessment.

a. All SABA use will be stopped at Visit 1 and participants will be switched to Sponsor-provided albuterol/salbutamol for use as needed throughout the study.
b. Short-term, occasional use of corticosteroids for the treatment of a COPD exacerbation is allowed.
c. Short-term, occasional use of antibiotics for infections or the treatment of a COPD exacerbation is allowed.
d. Any medications that, in the opinion of the Investigator, would impact the safety of the study or the outcome of the study is prohibited.
e. Participants who are on medication that have the potential to prolong the QTc interval may be enrolled provided the dose has remained stable for at least 12 weeks (3 months) prior to Visit 1, the participant meets none of the ECG exclusion criteria and if, in the opinion of the Investigator, there are no safety concerns for the participant in the study. Initiations of medications with potential to significantly prolong the QT interval is prohibited throughout the Screening and Randomized Treatment Periods. Note: Short courses (≤ 4 weeks) of antibiotics with the potential to prolong the QT interval (eg, azithromycin, clarithromycin) are permitted.
f. Investigators should contact the Study Physician to determine the appropriateness and safety of continuing study drug on a case-by-case basis (a monoclonal antibody for another indication, such as osteoporosis, may be allowed after consultation with the Study Physician).
g. If systemic anticholinergics are used for the treatment of overactive bladder and the treatment has been constant for at least 1 month, they are allowed.

6.5.3 Restricted Medications

Medications only allowed with defined stable dosing period prior to Visit 1 are listed in Table 6.

Table 6 Restricted medications

Medications Only Allowed with Defined Stable Dosing Period Prior to Visit 1	
Selective serotonin reuptake inhibitors/serotonin and norepinephrine reuptake inhibitors	4 weeks
Tricyclic antidepressants	6 weeks

Medications Only Allowed with Defined Stable Dosing Period Prior to Visit 1	
Antipsychotics	6 weeks
Anticonvulsants	52 weeks for seizure disorders [a]<br>12 weeks for other conditions
Non-sedating long- and short-acting antihistamines	7 days
Intranasal corticosteroids	7 days
Intranasal antihistamines or combination products of intranasal antihistamines/corticosteroids	7 days
Roflumilast	8 weeks
Inactive or killed vaccines per local policies including annual influenza vaccines	Allowed during study conduct but not 7 days prior visits with planned spirometry assessments

[a] Must be free of seizures for 1 year prior to Visit 1.

6.5.4 Rescue Medicine

6.5.4.1 SABA

Albuterol sulfate (US)/Salbutamol sulfate (EU/RoW) inhalation aerosol; (Albuterol 90 µg per actuation/Salbutamol 100 µg actuation).

Two inhalations as needed for rescue medication. As directed for eligibility testing.

6.6 Intervention After the End of the Study

Participants who complete Week 52/Visit 9/Withdrawal Visit (or Week 12/Visit 6/Withdrawal Visit for those not entering the extension study) should be given locally available standard-of-care treatment at the discretion of the Investigator.

7 DISCONTINUATION OF STUDY INTERVENTION AND PARTICIPANT DISCONTINUATION/WITHDRAWAL

7.1 Discontinuation of Study Intervention

It may be necessary for a participant to permanently discontinue study intervention. If study intervention is permanently discontinued, the participant should be encouraged to attend the on-site Withdrawal Visit (not later than within 7 days from last dose) and subsequent follow-up telephone contact within 2 weeks from last dose. See the SoA Table 1 for data to be collected at the time of discontinuation of study intervention and follow-up and for any further evaluations that need to be completed.

Note that discontinuation from study intervention is NOT the same thing as a withdrawal from the study.

If a participant experiences any of the changes listed below, the study intervention must be discontinued:

- Development of exclusion criteria or other safety reasons as judged by the Investigator during the treatment period;
- Pregnancy or breastfeeding;
- Paradoxical bronchospasm.

If a participant experiences the change of concern listed below, a repeat assessment should be obtained, and if confirmed, the study intervention must be discontinued:

- Calculated QTcF of either > 500 msec OR a ≥ 60 msec change from the value obtained at Visit 1;
- Decrease in eGFR to a value ≤ 30 mL/minute using CKD-EPI formula for participants 18 to 80 years of age, or a clinically relevant change from the Visit 1 value, as determined by the Investigator.

Participant may be discontinued from study intervention in the following situations:

- Participant decision. The participant is at any time free to discontinue study intervention, without prejudice to further treatment;
- An adverse event considered to jeopardize the safety of a participant participating in the study;
- Severe noncompliance with the CSP;
- Lost to follow up;
- Development of any study specific criteria for discontinuation;

- Any malignancy, except participants who develop basal cell carcinoma or localized squamous cell carcinoma of the skin, provided that the malignancy has been excised and determined to have clear margins;
- Other reasons.

See the SoA Table 1 for data to be collected at the Withdrawal (post study intervention discontinuation) and follow-up visits and for any further evaluations that need to be completed.

If a participant discontinues study intervention due to a study specific discontinuation criterion, this should always be recorded as ‘Development of Study Specific Discontinuation Criteria’ on the Discontinuation of Investigation Product form in the eCRF.

7.1.1 Temporary Discontinuation

The Sponsor reserves the right to temporarily suspend or permanently terminate this study or a component of the study at any time. The reasons for temporarily suspending the study may include, but are not limited, to any death, serious adverse events (SAE), or other safety finding assessed as related to study intervention that in the opinion of the Sponsor may preclude further study intervention.

7.2 Participant Withdrawal from the Study

A participant may withdraw from the study at any time at his/her own request, or at the request of their legally authorized representative, or may be withdrawn at any time at the discretion of the Investigator for safety, behavioral, compliance, or administrative reasons. This is expected to be uncommon.

A participant and/or their legally authorized representative who considers withdrawing from the study must be informed by the Investigator about modified follow-up options (eg, telephone contact, a contact with a relative or treating physician, or information from medical records).

If the participant and/or their legally authorized representative withdraws consent for disclosure of future information, the Sponsor may retain and continue to use any data collected before such a withdrawal of consent.

If a participant and/or their legally authorized representative withdraws from the study, it should be confirmed if the participant and/or their legally authorized representative still agrees for existing samples to be used in line with the original consent. If he/she and/or their legally authorized representative requests withdrawal of consent for use of samples, destruction of any samples taken and not tested should be carried out in line with what was stated in the informed consent and local regulation. The Investigator must document the decision on use of existing samples in the site study records and inform the Global Study Team.

7.3 Lost to Follow Up

A participant will be considered lost to follow-up if he or she repeatedly fails to return for scheduled visits and is unable to be contacted by the study site.

The following actions must be taken if a participant fails to return to the clinic for a required study visit:

- The site must attempt to contact the participant/legally authorized representative and reschedule the missed visit as soon as possible and counsel the participant/legally authorized representative on the importance of maintaining the assigned visit schedule and ascertain whether or not the participant/legally authorized representative wishes to and/or should continue in the study.
- Before a participant is deemed lost to follow-up, the Investigator or designee must make every effort to regain contact with the participant/legally authorized representative (where possible, 3 telephone calls and, if necessary, a certified letter to the participant's/legally authorized representative's last known mailing address or local equivalent methods). These contact attempts should be documented in the participant's medical record.
- Should the participant continue to be unreachable, he/she will be considered to have withdrawn from the study.

Discontinuation of specific sites or of the study as a whole are handled as part of Appendix A.

8 STUDY ASSESSMENTS AND PROCEDURES

Study procedures and their timing are summarized in the SoA (Table 1). Protocol waivers or exemptions are not allowed.

Immediate safety concerns should be discussed with the Sponsor immediately upon occurrence or awareness to determine if the participant should continue or discontinue study intervention.

Adherence to the study design requirements, including those specified in the SoA, is essential and required for study conduct.

All screening evaluations must be completed and reviewed to confirm that potential participants meet all eligibility criteria. The Investigator will maintain a screening log to record details of all participants screened and to confirm eligibility or record reasons for screening failure, as applicable.

Procedures conducted as part of the participant's routine clinical management (eg, blood count) and obtained before signing of the ICF may be utilized for screening or baseline

purposes provided the procedures met the protocol-specified criteria and were performed within the time frame defined in the SoA (Table 1).

8.1 Efficacy Assessments

8.1.1 Exploratory COPD Assessment Test (CAT)

The CAT is an 8-item patient reported outcome (PRO) developed to measure the impact of COPD on health status (Jones 2009). The instrument uses semantic differential six-point response scales which are defined by contrasting adjectives to capture the impact of COPD. Content includes items related to cough, phlegm, chest tightness, breathlessness going up hills/stairs, activity limitation at home, confidence leaving home, sleep, and energy.

A CAT total score is the sum of item responses. Scores range from 0-40 with higher scores indicative of greater COPD impact on health status. The CAT will be completed by the patient at Visits 1, 6, 9 and Withdrawal Visit as specified in the SoA (Table 1). If the subject is not able to visit the site, CAT may be completed by the subject at home/alternative location. Refer to Appendix F for details.

8.2 Safety Assessments

Planned time points for all safety assessments are provided in the SoA (see Table 1).

8.2.1 Physical Examinations

A complete physical examination and a brief physical examination will be performed at times as specified in the SoA (see Table 1). A complete physical examination will include an assessment of the following: general appearance, skin, head, eyes, ears, nose, throat, neck (including thyroid), lymph nodes, chest, heart, abdomen, extremities, and nervous system. A brief physical examination will include the evaluation of following: general appearance, respiratory, cardiovascular and abdomen.

Height and weight will be measure at Visit 1 (Screening).

8.2.2 Vital Signs

Vital sign measurements in this study will include sitting systolic and diastolic blood pressure, pulse rate, respiration rate, and body temperature.

Pulse rate should be measured before the blood pressure measurement. The pulse rate and blood pressure should be measured after the subject has been resting for at least 5 minutes. The measurement will be taken in a sitting position.

The respiration rate will be obtained after the subject has been resting for at least 5 minutes, by counting the number of breaths (how many times the chest rises) for 1 minute.

Body temperature will be recorded in Celsius in accordance with local standards.

Vital signs will be performed at times as specified in the SoA (see Table 1).

8.2.2.1 Weight and height

Weight and height will be measured in accordance with the SoA in Table 1. The subject's weight will be recorded in kilograms, and height will be recorded in centimeters. Weight and height measurements will be performed in light clothing and with shoes off.

8.2.3 Smoking Status

Smoking status will be assessed at every visit starting from enrollment (Visit 1) until their End-of-Treatment or Withdrawal Visit by collecting the subject's response to a single yes/no question from study personnel: 'What is your smoking status as of today, do you currently smoke?' Smoking status changes during Visit 3 until Withdrawal Visit will be captured on the eCRF but the subject will be permitted to continue in the study.

8.2.4 Electrocardiograms and Holter Monitoring - 24-Hour Continuous Electrocardiography

The 12-lead ECG will be taken in supine position after 10 minutes of rest at Visits 1, 4, 5, 9/Final Visit and Withdrawal Visit as specified in the SoA (Table 1). The Investigator or authorized delegate will be responsible for the overall interpretation and determination of clinical significance of any potential ECG findings. In case of discrepancy between the Investigator's interpretation and that provided by the ECG machine (if applicable), the Investigator's interpretation will take precedence and should be noted on the printout and recorded in the eCRF. A copy of the ECG will be produced, quality checked by site representatives and kept in case of further need for re-evaluation. ECG to be collected prior to any blood draws, spirometry, and IP administration.

The calculated QTcF intervals will be reviewed and checked for gross inaccuracies by the Investigator or designated ECG reviewer.

Participants who experience a change of QTcF intervals from their Visit 1 ECG may need to discontinue study intervention per criteria specified in Section 7.1.

A digital 12-lead Holter ECG will be performed at Visit 3 (randomization)/Visit 6 with the purpose of gathering timely relevant ECG, cardiac rhythm data and capture potential pro-arrhythmic reactions.

Continuous Digital 12-lead Holter ECG will be connected the day prior to Visit 3 (randomization)/Visit 6 to allow for 24 hours ECG recordings and ECG extractions at the following time points: 60 min pre-dose and 20 min, 60 min & 4 hr post dose (see Table 2).

Note that the 60 min pre-dose and 60 min post dose ECG extractions should be completed prior to the spirometry assessments at those timepoints.

Holter hook up procedure should be completed/ECG recordings are to be initiated 1 day prior to scheduled Visit 3 and 6 at approximately the same time within the following time window 13:00 (±2 hr) for both visits. Holter Monitor will be removed from the study participant at Visit 3 and 6 following collection of ECG recordings at 4h post IP dose as specified in SoA Table 1 and Table 2. All Holter Monitor recordings will be assessed for cardiac arrhythmias by an independent cardiologist.

The same device type will be used across all sites in this study to collect all 12-lead safety ECGs, 24-hour Holter recordings, and related digital 12-lead ECG extractions from Holter recordings. Twenty-four-hour Holter recordings (collected at Visit 3 and Visit 6) will be transferred to an external vendor, where qualified personnel will extract digital 12-lead ECGs from the recordings (to support timepoints specified in Table 2) and perform centralized assessment of provided data to assure data quality and consistency in data interpretation.

The same centralized assessment and data interpretation will be applied to all 12-lead safety ECGs (collected at Visits 1, 4, 5, 9/Final Visit, and Withdrawal Visit), as related ECG recordings in digital/electronic format will be shared with the external vendor. Results of related assessments will be shared with Investigators. In case of significant abnormalities identified in collected ECG/Holter data, Investigators will be contacted by the vendor to provide information on detected abnormalities.

More details will be present in manual covering details for 12-lead ECG/Holter monitoring.

8.2.5 Spirometry

Forced expiratory spirometry maneuvers for derivation of FEV1, FVC, FEV1/FVC ratio as defined in American Thoracic Society (ATS)/European Respiratory Society (ERS) guidelines will be performed in accordance with ATS/ERS acceptability and repeatability criteria will be assessed using a spirometer that meets or exceeds minimum performance recommendations. Spirometry must meet both acceptability and repeatability criteria according to ATS/ERS 2019 recommendations. Calculated predicted spirometry results will be obtained using the Global Lung Initiative (GLI) equations (Quanjer 2012).

All study staff responsible for performing spirometry assessments will receive standardized training and will be required to pass spirometry proficiency test to demonstrate proficiency in the use of the equipment and the ability to perform technically acceptable spirometry assessments (ATS criteria) prior to performing any spirometry assessments for the purpose of this study.

Spirometry collection is briefly outlined below. For exact spirometry collection and specifications, please refer to the SoA, refer to Table 1 and Table 2. Spirometry will be conducted at all site visits.

At Visit 1, trough spirometry will be conducted approximately 60 to 30 min prior to standard time for morning COPD maintenance treatment administration; at Visit 2, spirometry will be conducted approximately 60 to 30 min prior to bronchodilator administration and approximately 30 to 60 min post bronchodilator. If participants are not reversible to albuterol at 30 minutes post-dose, the post-dose spirometry should be repeated at 60 minutes post-dose.

At Visits 3 to 9 (including any needed Unscheduled Visits, if needed), spirometry will be obtained 30 min before the study drug administration, and post dosing of study drug at 5, 15, 30 and 60 min (see Table 2).

During withdrawal (WD) visit, trough spirometry will be conducted approximately 60 to 30 min prior to standard time for morning dose of discontinued study intervention or new standard COPD maintenance treatment.

The procedure for testing reversibility to albuterol at Visit 2 is as follows:

1. Confirm that COPD medication washout criterion was met, including salbutamol or albuterol was withheld for at least 6 hours prior to the start time of the spirometry.
2. Perform pre-bronchodilator spirometry at approximately 60 to 30 minutes prior to administration of bronchodilator.
3. Administer 4 puffs of albuterol.
4. Perform post-bronchodilator spirometry 30 minutes after the administration of albuterol. If participants are not reversible to albuterol at 30 minutes, the post-dose spirometry should be repeated at 60 minutes post-dose.

Reversibility will be a comparison of the average best FEV1 effort obtained at approximately 60 to 30-minutes pre-bronchodilator to the best FEV1 effort obtained at 30 minutes (or up to 60 minutes, if repeated) post-bronchodilator following administration of albuterol.

8.2.6 Clinical Safety Laboratory Assessments

The clinical chemistry and hematology will be performed at a central laboratory, except for urine human chorionic gonadotropin (hCG) test which will be performed at study site (dipstick). All protocol-required laboratory assessments must be conducted in accordance with the laboratory manual.

All clinical laboratory tests will be obtained at Visit 1 (Screening) and at Visit 3 (Randomization), Visit 6 (Week 12), and Visit 9 (Week 52), End-of-treatment/Withdrawal Visit, and any Unscheduled Visit (if needed, at the discretion of the Investigator), see SoA Table 1.

The Investigator should assess the available results regarding clinically relevant abnormalities. The laboratory results should be signed and dated and retained at center as source data for laboratory variables. The laboratory variables listed in Table 7 will be measured.

For information on how AEs based on laboratory tests should be recorded and reported, see Section 8.3.5.

If an emergent event results in centralized laboratory testing of samples not being available, the Sponsor may decide to use local laboratories.

Table 7 Clinical Laboratory Tests

**Screening (and as applicable during 12-week and 52-week Treatment periods [see Table 1])**	
**Hematology/Hemostasis (whole blood)**	**Clinical Chemistry (serum or plasma)**
B-Hemoglobin (Hb)	Creatinine
B-Leukocyte count	Bilirubin, total
B-Leukocyte differential count (absolute count)	Alkaline phosphatase (ALP)
B-Platelet count	Aspartate transaminase (AST)
	Alanine transaminase (ALT)
S-β-hCG (Pregnancy test) at Visit 1	Potassium
U-β-hCG pregnancy for all visits except Visit 1	Sodium
S-FSH[a] at Visit 1 only	Glucose
eGFR[b]	
Troponin[c]	

a. Serum FSH test is only required for eligibility check. FSH is tested at Visit 1 for women <50 years of age with amenorrhea for 12 months without an alternative medical cause.
b. eGFR estimated by the CKD-EPI formula (Levey 2009) for participants 18 to 80 years of age to be calculated at Visit 1, 3, 6, 9 and Withdrawal Visit.
c. Troponin use only as a baseline measurement in the study at Visit 3 prior study intervention administration. Post randomization troponin should be checked according to the Investigator's decision and only when clinically indicated for potential Adverse Events diagnosis/reporting.

8.2.6.1 Hematology

Hemoglobin, white blood cell count with differential, platelet count will be measured at Visit 1 (Screening) and at Visit 3 (Randomization), Visit 6 (Week 12) and Visit 9 (Week 52) and the End-of-treatment/Withdrawal Visit.

8.2.6.2 Clinical chemistry

Creatinine, total bilirubin, alkaline phosphatase (ALP), aspartate aminotransferase (AST), alanine aminotransferase (ALT), glucose, potassium and sodium, will be measured at Visit 1 (Screening) and at Visit 3 (Randomization), Visit 6 (Week 12) and Visit 9 (Week 52) and the End-of-treatment/Withdrawal Visit.

8.2.6.3 Pregnancy test

Serum pregnancy test will be performed at Visit 1 (Screening) for women of childbearing potential.

The following tests are applicable to female subjects only and will be conducted in accordance with the schedule provided in SoA Table 1.

- Serum ß-human chorionic gonadotropin (ß-hCG) – the test done at enrollment (Visit 1) only, for WOCBP (analyzed at central laboratory).
- FSH – the test done at enrollment (Visit 1) only, for female subjects to confirm postmenopausal status in women <50 years who have been amenorrhoeic for > 12 months (analyzed at central laboratory).
- Urine β-hCG – the test will be performed at the study site for WOCBP at each visit before IP administration using a dipstick. Positive urine β-hCG test result must be confirmed with serum β-hCG analyzed at central laboratory.

8.3 Adverse Events and Serious Adverse Events

The PI is responsible for ensuring that all staff involved in the study are familiar with the content of this section.

The definitions of an AE or SAE can be found in Appendix B.

The definitions of medical device-related safety events, (Medical device (SAE), adverse device effects [ADEs] and SADEs), can be found in Appendix F. Medical Device deficiencies are covered in Section 8.3.11.

Adverse events will be reported by the participant (or, when appropriate, by a caregiver, surrogate, or the participant's legally authorized representative).

The Investigator and any designees are responsible for detecting, documenting, and recording events that meet the definition of an AE.

8.3.1 Time Period and Frequency for Collecting AE and SAE Information

Adverse events will be collected from randomization, Visit 3 throughout the treatment period and including the follow-up period/last contact.

Throughout the study AEs will be captured every 2 weeks via telephone contact and/or site visits (telephone: Weeks 2, 6, 10, 14, 16, 18, 20, 22, 24, 28, 30, 32, 34, 36, 38, 42, 44, 46, 48, 50, and 54; in-person site visits: Weeks 0, 4, 8, 12, 26, 40, and 52, as well as at any applicable Unscheduled or Withdrawal visits; see SoA in Table 1).

All SAEs will be recorded from the time of signing of the ICF, throughout the treatment period and including the follow-up period, up to the final visit.

All AEOSIs will be recorded from the time of the first dose, throughout the treatment period and including the follow-up period, up to the final visit.

If the Investigator becomes aware of an SAE with a suspected causal relationship to the investigational medicinal product that occurs after the end of the clinical study in a participant treated by him or her, the Investigator shall, without undue delay, report the SAE to the Sponsor.

8.3.2 Follow-up of AEs and SAEs

Any AEs that are unresolved at the participant’s last AE assessment in the study are followed-up by the Investigator for as long as medically indicated, but without further recording in the eCRF. AstraZeneca retains the right to request additional information for any participant with ongoing AE(s)/SAE(s) at the end of the study, if judged necessary.

Adverse event variables

The following variables will be collected for each AE:

- AE (verbatim)
- The date when the AE started and stopped
- Maximum intensity
- Whether the AE is serious or not
- Investigator causality rating against the Investigational Product(s) (yes or no)
- Action taken with regard to Investigational Product(s)

- AE caused participant's withdrawal from study (yes or no)
- Outcome

In addition, the following variables will be collected for SAEs:

- Date AE met criteria for SAE
- Date Investigator became aware of SAE
- AE is serious due to
- Date of Hospitalization
- Date of discharge
- Probable cause of death
- Date of death
- Autopsy performed
- Causality assessment in relation to study procedure(s)
- Causality assessment to other medication.

Maximum intensities will be reported for each AE. Maximum intensity refers to the complete course of the AE. The participants will be asked to assess the maximum intensity of the reported AEs according to the following scale:

- Mild (awareness of sign or symptoms, but easily tolerated)
- Moderate (discomfort sufficient to cause interference with normal activities)
- Severe (incapacitating, with inability to perform normal activities).

8.3.3 Causality Collection

The Investigator should assess causal relationship between study intervention and each AE, and answer 'yes' or 'no' to the question 'Do you consider that there is a reasonable possibility that the event may have been caused by the investigational product?'

For SAEs, causal relationship should also be assessed for other medication and study procedures. Note that for SAEs that could be associated with any study procedure the causal relationship is implied as 'yes'.

A guide to the interpretation of the causality question is found in Appendix B to this CSP.

8.3.4 Adverse Events Based on Signs and Symptoms

All AEs spontaneously reported by the participant or legally authorized representative or reported in response to the open question from the study site staff: 'Have you had any health problems since the previous visit/you were last asked?', or revealed by observation will be collected and recorded in the eCRF. When collecting AEs, the recording of diagnoses is

preferred (when possible) to recording a list of signs and symptoms. However, if a diagnosis is known and there are other signs or symptoms that are not generally part of the diagnosis, the diagnosis and each sign or symptom will be recorded separately.

8.3.5 Adverse Events Based on Examinations and Tests

The results from the CSP-mandated laboratory tests and vital signs will be summarized in the Clinical Study Report (CSR).

Deterioration as compared to baseline in protocol-mandated vital signs should therefore only be reported as AEs if they fulfill any of the SAE criteria, are the reason for discontinuation of treatment with the investigational product or are considered to be clinically relevant as judged by the Investigator (which may include but not limited to consideration as to whether treatment or non-planned visits were required or other action was taken with the study intervention, eg, dose adjustment or drug interruption).

If deterioration in a laboratory value/vital sign is associated with clinical signs and symptoms, the sign or symptom will be reported as an AE and the associated laboratory result/vital sign will be considered as additional information. Wherever possible the reporting Investigator uses the clinical, rather than the laboratory term (eg, anemia versus low hemoglobin value). In the absence of clinical signs or symptoms, clinically relevant deteriorations in non-mandated parameters should be reported as AE(s).

All 12-lead ECG abnormal findings and digital 12-lead Holter ECG abnormal findings obtained within the study will be summarized in the CSR; therefore, they should not by default be reported individually as AEs. Abnormal findings from 12-lead ECGs or from digital 12-lead Holter ECGs should only be reported as AEs if they fulfill any of the SAE criteria, are the reason for discontinuation of treatment with the investigational product, or are considered to be clinically relevant as judged by the Investigator (which may include but is not limited to consideration as to whether treatment or non-planned visits were required or other action was taken with the study intervention, eg, drug interruption).

Any new or aggravated clinically relevant abnormal medical finding at a physical examination as compared with the baseline assessment will be reported as an AE.

8.3.6 Adverse Events of Special Interest

The AEOSIs in this study are respiratory events such as dysphonia, cough, dyspnea, wheezing, paradoxical bronchospasm, bronchospasm, and COPD exacerbations.

For handling of AEOSI that are also symptoms of Disease under Study (DUS) please refer to Section 8.3.7.

If any of the AEOSI are reported the participants will be asked about time relatedness to inhalation.

8.3.6.1 Paradoxical bronchospasm

Paradoxical bronchospasm is defined as a reduction in FEV1 of >15% from baseline (ie, the FEV1 value obtained within 30 minutes prior to study intervention administration on that same day) that occurs shortly after dosing with associated symptoms of wheezing, shortness of breath, or cough. A drop in FEV1 >15% from baseline with associated symptoms should be reported as an AE of paradoxical bronchospasm.

Monitoring for paradoxical bronchospasm will occur at each in-clinic visit by spirometry assessments at 5 and 15 minutes post-dose. If FEV1 drops >15% from baseline, measurements should be repeated until FEV1 values have normalized.

Monitoring of FEV1 will also be done at 30 and 60 minutes post-dose in order to provide more robust post-dose surveillance than is possible with the 5- and 15-minutes post-dose recordings included in the paradoxical bronchospasm definition.

8.3.6.2 Level of COPD severity by airflow limitation

Clinic Site Investigator will document most recent post-bronchodilator FEV1 % predicted.

8.3.6.3 COPD exacerbation assessment

<u>Assessment of COPD exacerbation</u>

For the purpose of this CSP, a moderate or severe COPD exacerbation will be defined as a worsening in the participant's usual COPD symptoms that is beyond normal day-to-day variation, is acute in onset, lasts 2 or more days (or less if the worsening is so rapid and profound that the treating physician judges that intensification of treatment cannot be delayed), may warrant a change in regular medication, and leads to any of the following:

- Use of systemic corticosteroids for at least 3 days; a single depot injectable dose of corticosteroids will be considered equivalent to a 3-day course of systemic corticosteroids
- Use of antibiotics to treat COPD exacerbation for at least 3 days
- An inpatient hospitalization due to COPD (defined as an inpatient admission ≥ 24 hours in the hospital, an observation area, the emergency department, or other equivalent healthcare facility depending on the country and healthcare system)
- Admission in emergency department or emergency room due to COPD for < 24 hours requiring intensive treatment; add per study requirement. The list of intensive treatment will be included in the eCRF instruction, please refer to the standard
- Results in death.

During a COPD exacerbation, it is important for participants to be treated as deemed appropriate by the treating healthcare provider. However, all supplemental medication used to treat the COPD exacerbation should be discontinued as soon as it is considered safe by the Investigator and the participant should return to their pre-exacerbation medication regimen as soon as it is practical to do.

The participant may remain in the study after an exacerbation and continue to receive study intervention if the Investigator judges that it is medically appropriate.

Study site evaluations for a COPD worsening may occur as a part of a pre-scheduled site visit, or as an Unscheduled visit, if deemed necessary by the Investigator. A copy of the medical record should be obtained for exacerbations evaluated and treated at non-study sites (eg, by the primary care HCP or at an emergency department/hospital) and details entered into the eCRF exacerbation module in a timely fashion. Changes in concomitant medication due to an exacerbation must be recorded in the appropriate eCRF.

Severity of COPD exacerbations

A COPD exacerbation will be considered moderate if it requires treatment with systemic corticosteroids and/or antibiotics for at least 3 days, or resulted in emergency room visit < 24h requiring intensive treatment; and does not result in hospitalization or death.

A COPD exacerbation will be considered severe if it results in hospitalization (defined as an inpatient admission ≥ 24h in the hospital, an observation area, the emergency department, or other equivalent healthcare facility depending on the country and healthcare system) or death due to COPD.

Symptoms reporting

Participant will be encouraged to contact the study site and/or their primary care physician in case of COPD symptoms worsening. The participant will be asked to inform the Investigator of any COPD-related events treated outside of the study site. For COPD symptoms reporting, see DUS (Section 8.3.7).

Duration of COPD exacerbation

The start and stop of a moderate or severe exacerbation is defined as:

- The start date of systemic corticosteroids or antibiotic treatment or hospital admission, whichever occurs earlier, and the stop date is defined as the last day of systemic corticosteroids or antibiotic treatment or hospital discharge, whichever occurs later.
- A single depot injectable dose of corticosteroids will be considered equivalent to a 3-day course of systemic corticosteroids. The corresponding stop date for this treatment will consequently be determined as the date of administration plus 2 days.

- If multiple treatments are prescribed for the same exacerbation, the earliest start date and the latest stop date will be used.

For a severe COPD exacerbation with no documented corticosteroid or antibiotics treatment, hospitalization admission/discharge dates, or emergency visit date will be used as start/stop dates.

Participants will be queried at Visit 1, Visit 2 (as applicable), and Visit 3 as to whether they have experienced a COPD exacerbation, defined as an acute event characterized by a worsening of the participant's respiratory symptoms that is beyond normal day-to-day variations and leads to a change in COPD medications. In addition, if the Study Physician responds to calls from the participant or elevated symptoms reported by the participant, they may determine that the participant is experiencing an exacerbation.

8.3.7 Disease Under Study

Symptoms of DUS are those which might be expected to occur as a direct result of COPD. Events which are unequivocally due to DUS should not be reported as an AE during the study unless:

- they meet SAE criteria
- there is a COPD worsening that meets the protocol definition for COPD exacerbation
- the sign or symptom are new and/or different from the participants COPD history/baseline regardless of serious criteria.

8.3.8 Reporting of Serious Adverse Events

All SAEs have to be reported, whether or not considered causally related to the investigational product, or to the study procedure(s). All SAEs will be recorded in the eCRF.

If any SAE occurs in the course of the study, investigators or other site personnel will inform the appropriate AstraZeneca representatives within 1 day, ie, immediately but **no later than 24 hours** of when he or she becomes aware of it.

The designated AstraZeneca representative will work with the Investigator to ensure that all the necessary information is provided to the AstraZeneca Patient Safety data entry site **within 1 calendar day** of initial receipt for fatal and life-threatening events **and within 5 calendar days** of initial receipt for all other SAEs.

For fatal or life-threatening AEs where important or relevant information is missing, active follow-up will be undertaken immediately. Investigators or other site personnel will inform AstraZeneca representatives of any follow-up information on a previously reported SAE

within 1 calendar day, ie, immediately but **no later than 24 hours** of when he or she becomes aware of it.

Once the investigators or other site personnel indicate an AE is serious in the electronic data capture (EDC) system, an automated e-mail alert is sent to the designated AstraZeneca representative. If the EDC system is not available, then the Investigator or other study site staff reports a SAE to the appropriate AstraZeneca representative by telephone. The Sponsor representative will advise the Investigator/study site staff how to proceed.

The reference document for definition of expectedness/listedness is the IB for the AstraZeneca investigational product and the AstraZeneca active comparator.

For further guidance on the definition of a SAE, see Appendix B of the CSP.

8.3.9 Pregnancy

All pregnancies and outcomes of pregnancy should be reported to AstraZeneca except for:

- If the pregnancy is discovered before the study participant has received any study intervention (see Exclusion Criteria, Section 5.2)
- Pregnancies in the partner of male participants.

8.3.9.1 Maternal exposure

Female participants who are pregnant or have a confirmed positive pregnancy test at screening or Day 1 will be excluded from the study (see Section 5.2). If a participant becomes pregnant during the course of the study, IP should be discontinued immediately.

Pregnancy itself is not regarded as an AE unless there is a suspicion that the investigational product under study may have interfered with the effectiveness of a contraceptive medication. Congenital abnormalities/birth defects and spontaneous miscarriages should be reported and handled as SAEs. Elective abortions without complications should not be handled as AEs. The outcome of all pregnancies (spontaneous miscarriage, elective termination, ectopic pregnancy, normal birth or congenital abnormality) should be followed up and documented even if the participant was discontinued from the study.

If any pregnancy occurs in the course of the study, then the Investigator or other site personnel informs the appropriate AstraZeneca representatives within **1 day**, ie, immediately but **no later than 24 hours** of when he or she becomes aware of it.

The designated Sponsor representative works with the Investigator to ensure that all relevant information is provided to the AstraZeneca Patient Safety data entry site **within 1 or 5 calendar days** for SAEs (see Section 8.3.8) and **within 30 days** for all other pregnancies.

The same timelines apply when outcome information is available.

The PREGREP module in the eCRF is used to report the pregnancy and the paper based PREGOUT module is used to report the outcome of the pregnancy.

8.3.9.2 Paternal exposure

There is no restriction on fathering children or donating sperm during the study.

8.3.10 Medication Error, Drug Abuse, and Drug Misuse

8.3.10.1 Timelines

If an event of medication error, drug abuse, **or** drug misuse occurs during the study, then the investigator or other site personnel informs the appropriate AstraZeneca representatives within **one calendar day**, ie, immediately but **no later than 24 hours** of when they become aware of it.

The designated AstraZeneca representative works with the investigator to ensure that all relevant information is completed within **one** (initial fatal/life-threatening or follow-up fatal/life-threatening) **or 5** (other serious initial and follow-up) **calendar days** if there is an SAE associated with the event of medication error, drug abuse, or misuse (see Section 8.3.8) and **within 30 days** for all other events.

8.3.10.2 Medication Error

For the purposes of this clinical study a medication error is an unintended failure or mistake in the treatment process for an IMP or AstraZeneca NIMP that either causes harm to the participant or has the potential to cause harm to the participant.

The full definition and examples of medication error can be found in Appendix B 4.

8.3.10.3 Drug Abuse

Drug abuse is the persistent or sporadic **intentional**, non-therapeutic excessive use of IMP or AstraZeneca NIMP for a perceived reward or desired non-therapeutic effect.

The full definition and examples of drug abuse can be found in Appendix B 4.

8.3.10.4 Drug Misuse

Drug misuse is the **intentional** and inappropriate use (by a study participant) of IMP or AstraZeneca NIMP for medicinal purposes outside of the authorized product information, or for unauthorized IMPs or AstraZeneca NIMPs, outside the intended use as specified in the protocol and includes deliberate administration of the product by the wrong route.

The full definition and examples of drug misuse can be found in Appendix B 4.

8.3.11 Medical Device Deficiencies

Medical devices, the MDIs, are being provided for use in this study as the study intervention is supplied in the visiting clinic by AstraZeneca and are being utilized to deliver the intervention under study. In order to fulfill regulatory reporting obligations worldwide, the Investigator is responsible for the detection and documentation of events meeting the definitions of Medical Device Deficiency that occur during the study with such medical devices.

The definition of a Medical Device Deficiency can be found in Appendix E.

NOTE: Incidents and deficiencies fulfilling the definition of an AE/SAE will also follow the processes outlined in Appendix E of this protocol.

The AstraZeneca or manufacturers medical device complaint report will be used to collect the deficiency.

8.3.11.1 Time period for detecting Medical Device Deficiencies

Medical device incidents or malfunctions of the medical device will be detected, documented, and reported during all periods of the study in which the medical device is used.

If the Investigator learns of any Medical Device Deficiency at any time after a participant has been discharged from the study, and such incident is considered reasonably related to a medical device provided for the study, the Investigator will promptly notify the Sponsor.

The method of documenting Medical Device Deficiency is provided in Appendix E.

8.3.11.2 Follow-up of Medical Device Deficiencies

Follow-up applies to all participants, including those who discontinue study intervention.

The Investigator is responsible for ensuring that follow-up includes any supplemental investigations as indicated to elucidate the nature and/or causality of the deficiency.

New or updated information will be recorded on the originally completed form with all changes signed and dated by the Investigator.

8.3.11.3 Prompt reporting of Medical Device Deficiencies to Sponsor

Medical device deficiencies will be reported to the Sponsor within 24 hours after the Investigator determines that the event meets the protocol definition of a Medical Device Deficiency.

The Medical Device Deficiency Paper Report Form will be sent to the Sponsor by e-mail. If e-mail is unavailable, then telephone should be utilized.

The Sponsor will be the contact for the receipt of Medical Device Deficiency reports.

8.3.11.4 Regulatory reporting requirements for Medical Device Deficiencies

The Investigator will promptly report all medical device deficiencies occurring with any medical device provided for use in the study in order for the Sponsor to fulfil the legal responsibility to notify appropriate regulatory authorities and other entities about certain safety information relating to medical devices being used in clinical studies.

The Investigator, or responsible person according to local requirements (eg, the head of the medical institution), will comply with the applicable local regulatory requirements relating to the reporting of medical device deficiencies to the IRB/Independent Ethics Committee (IEC).

For further guidance on the definition of an SAE, see Appendix B 2 of the CSP.

8.4 Overdose

For guidance refer to AstraZeneca SOP Reporting of Individual Safety Events in Clinical Studies.

More than 2 inhalations of BGF MDI (budesonide 160 micrograms/ glycopyrronium bromide 7.2 micrograms/ formoterol fumarate dihydrate 4.8 micrograms per actuation) twice daily, in the morning and in the evening, is considered an overdose.

- An overdose with associated AEs is recorded as the AE diagnosis/symptoms on the relevant AE modules in the eCRF and on the Overdose eCRF module.
- An overdose without associated symptoms is only reported on the Overdose eCRF module.

If an overdose on an AstraZeneca study intervention occurs in the course of the study, the Investigator or other site personnel inform appropriate AstraZeneca representatives immediately, but **no later than 24 hours** of when he or she becomes aware of it.

The designated AstraZeneca representative works with the Investigator to ensure that all relevant information is provided to the AstraZeneca Patient Safety data entry site **within one or 5 calendar days** for overdoses associated with an SAE (see Section 8.3.8) and **within 30 days** for all other overdoses.

8.4.1 Budesonide

Acute overdosage with budesonide, even in excessive doses, is not expected to be a clinical problem. When used chronically in excessive doses, systemic glucocorticosteroid effects may appear.

8.4.2 Glycopyrronium

An overdose of glycopyrronium may lead to exaggerated anticholinergic signs and symptoms, the most frequent of which include blurred vision, dry mouth, nausea, headache, palpitations, and systolic hypertension. However, there were no systemic anticholinergic adverse effects following single inhaled doses up to 144 μg in patients with COPD.

8.4.3 Formoterol

An overdose of formoterol would likely lead to effects that are typical for β_2-adrenergic agonists: tremor, headache, palpitations, and tachycardia. Hypotension, metabolic acidosis, hypokalemia, and hyperglycemia may also occur. Supportive and symptomatic treatment may be indicated. A dose of 90 μg administered during 3 hours in patients with acute bronchial obstruction raised no safety concerns.

If overdose occurs, including any combination of the above, the participant should be treated supportively with appropriate monitoring as necessary.

8.5 Optional Genomics Initiative Sample

- Collection of optional samples for Genomics Initiative research is also part of this study as specified in the SoA and is subject to agreement in the ICF addendum.

A blood sample for DNA isolation will be collected from participants who have consented to participate in the genetic analysis component of the study at randomization/baseline (Visit 3) (Table 1). Participation is optional. Participants who do not wish to participate in the genetic research may still participate in the study. Samples can be collected at any time after the genetic ICF is signed but is recommended at Visit 3.

See Appendix D for information regarding genetic research. Details on processes for collection and shipment and destruction of these samples are provided in Appendix D and in the Laboratory Manual.

9 STATISTICAL CONSIDERATIONS

9.1 Statistical Hypotheses

No formal statistical hypotheses will be tested.

9.2 Sample Size Determination

For the 12-week study, 542 participants will be randomized to treatments BGF MDI HFO and BGF MDI HFA in a 1:1 ratio. This is to achieve a 90% probability of observing at least 1 AE in each treatment arm with an expected cumulative incidence of 1% (CPMP/ICH/375/95), and assuming a 15% dropout rate.

Subsequently, the 120 participants per treatment arm who were randomized to the extended study will continue and remain on the randomized treatment to 52 weeks. Accounting for the dropouts, the aim is to achieve approximately 100 completers per arm at 52 weeks (ICH E1A).

The randomization scheme of 542 participants during 12-week study and the selection of 120 participants per treatment arm for the extended study will be planned prior to the first dose of study treatment.

9.3 Populations for Analyses

For the purposes of analysis, the following populations are defined:

Table 8 Populations for Analysis

Population	Description
12-week safety analysis set	All participants who were randomized to study treatment and received at least one dose, irrespective of their protocol adherence and whether they would continue the extended study. Participants will be analyzed according to their treatment received.
52-week safety analysis set	A subset of participants who were randomized to study treatment and received at least one dose, and were selected to continue the extended study, irrespective of their protocol adherence. Participants will be analyzed according to their treatment received.

9.4 Statistical Analyses

The analysis for 12-week study will be performed on the 12-week safety analysis set.

The analysis for 52-week study will be performed on the 52-week safety analysis set.

The analyses described in this section will apply to both the 12-week study and 52-week study, unless specified otherwise.

Details of all statistical analyses will be documented in the Statistical Analysis Plan (SAP). The SAP will be finalized prior to unblinding of the data for the analysis.

9.4.1 General Considerations

Analyses will be performed by the Sponsor or its representatives. Demographic and baseline characteristics data will be summarized by treatment. Categorical variables will be summarized using frequency and percentages, where the denominator for calculation is the underlying analysis set population unless otherwise specified. Continuous variables will be summarized with descriptive statistics using number of available observations, mean, standard deviation, median, minimum, and maximum, and quartiles where appropriate.

9.4.2 Efficacy Analysis

9.4.2.1 Exploratory CAT assessment

The CAT assessment will be summarized by treatment and visit. The change from baseline in CAT total score will be summarized by treatment and visit.

9.4.3 Safety Analysis

The safety analyses will be based on the safety analysis sets and will include all the patients who had received at minimum one exposure to the intervention. All AEs should be reported, whether or not they are considered to be related to treatment. All available data in the study population should be accounted for in the evaluation. Exposure extent, safety, and tolerability will be compared qualitatively and reported between arms. The following will be reported:

All AEs beginning during the treatment or follow-up period will be summarized descriptively by treatment arm, at the level of the MedDRA PT and SOC, for the Safety analysis sets. The summaries for each treatment arm will include the number and percentage of participants experiencing the event and the number of events.

For AEOSIs, AEs leading to discontinuation of IP, and SAEs, exposure-adjusted incident rates and the associated 95% CI will be presented. The rate difference between treatment arms and the associated 95% CIs will also be reported. No formal hypothesis tests will be performed. Details will be provided in the statistical analysis plan.

Additional safety variables, such as concomitant medications, vital signs, physical examination, clinical laboratory, and ECG status will be summarized with the percentage of patients in different categories.

9.4.3.1 Adverse events

The rate of AEs during the treatment period will be summarized by means of descriptive statistics and qualitative summaries. In addition to all AEs, the summary will include SAEs, AEs leading to treatment discontinuation, and AEOSIs as defined in Section 8.3.6.

- **Paradoxical Bronchospasm** - The exposure-adjusted incidence rate of paradoxical bronchospasm (defined in AEOSI Section 8.3.6) will be summarized. The rate difference between treatment arms and the 95% CI will be evaluated.

9.4.3.2 12-lead ECG

Summary statistics for absolute values and change from baseline will be tabulated by ECG parameter and treatment for each scheduled assessment time. For analysis of 12-lead ECG parameters, baseline will be defined as the mean of the latest triplicate measurements extracted from the Holter monitor at 60 minutes prior to dosing at Visit 3. Assessments that are not evaluable will be excluded. In addition, potentially clinically significant values will be identified and summarized.

For bedside assessments made by the Investigator, 12-lead ECG parameter baseline values are defined as those collected at screening (Visit 1).

9.4.3.3 Digital 12-lead Holter ECG

Summary statistics for raw values and changes from baseline in QTcF intervals will be calculated. The assessments will be tabulated for each treatment and assessment time. Baseline definition and details of digital 12-lead Holter ECG analyses will be provided in the SAP.

9.4.3.4 Clinical laboratory values

Summary statistics of change from baseline values will be tabulated for each treatment and scheduled assessment time. For clinical laboratory measurements, baseline will be defined as the last available value prior to dosing on the day of or prior to randomization. Potentially clinically significant values will be identified and summarized.

9.4.3.5 Vital signs

Summary statistics of change from baseline will be tabulated by vital sign parameter and treatment for each scheduled assessment time. For vital signs, baseline will be defined as the last available value prior to dosing on the day of or prior to randomization. In addition, potentially clinically significant values will be identified and summarized.

9.4.3.6 Spirometry measurement

The comparison of FEV1 post-dose evaluation at 5, 15, 30, 60 minutes with pre-dose FEV1 obtained on the same day will be summarized.

9.5 Interim Analyses

No Interim Analysis is applied to this study.

9.6 Data Monitoring Committee

Not applicable - no DMC is planned.

10 SUPPORTING DOCUMENTATION AND OPERATIONAL CONSIDERATIONS

Appendix A Regulatory, Ethical, and Study Oversight Considerations

A 1 Regulatory and Ethical Considerations

- This study will be conducted in accordance with the protocol and with the following:
  - Consensus ethical principles derived from international guidelines including the Declaration of Helsinki and Council for International Organizations of Medical Sciences International Ethical Guidelines
  - Applicable International Council for Harmonisation (ICH) GCP Guidelines
  - Applicable laws and regulations
- The protocol, protocol amendments, ICF, Investigator Brochure, and other relevant documents (eg, advertisements) must be submitted to an IRB/IEC by the Investigator and reviewed and approved by the Institutional Review Board (IRB)/IEC before the study is initiated.
- Any amendments to the protocol will require IRB/IEC and applicable Regulatory Authority approval before implementation of changes made to the study design, except for changes necessary to eliminate an immediate hazard to study participants.
- AstraZeneca will be responsible for obtaining the required authorizations to conduct the study from the concerned Regulatory Authority. This responsibility may be delegated to a Contract Research Organization but the accountability remains with AstraZeneca.
- The Investigator will be responsible for providing oversight of the conduct of the study at the site and adherence to requirements of 21 Code of Federal Regulations (CFR), ICH guidelines, the IRB/IEC, European Regulation 536/2014 for clinical studies (if applicable), European Medical Device Regulation 2017/745 for clinical device research (if applicable), and all other applicable local regulations

Regulatory Reporting Requirements for SAEs

- Prompt notification by the Investigator to the Sponsor of a SAE is essential so that legal obligations and ethical responsibilities towards the safety of participants and the safety of a study intervention under clinical investigation are met.
- The Sponsor has a legal responsibility to notify both the local regulatory authority and other regulatory agencies about the safety of a study intervention under clinical investigation. The Sponsor will comply with country-specific regulatory requirements relating to safety reporting to the regulatory authority, IRB/IEC, and investigators.

- For all studies except those utilizing medical devices, Investigator safety reports must be prepared for suspected unexpected serious adverse reactions according to local regulatory requirements and Sponsor policy and forwarded to investigators as necessary.
  - European Medical Device Regulation 2017/745 for clinical device research (if applicable), and all other applicable local regulations
- An Investigator who receives an Investigator safety report describing a SAE or other specific safety information (eg, summary or listing of SAEs) from the Sponsor will review and then file it along with the IB and will notify the IRB/IEC, if appropriate according to local requirements.

Regulatory Reporting Requirements for Serious Breaches of Protocol or GCP

Prompt notification by the Investigator to AstraZeneca of any (potential) serious breach of the protocol or regulations is essential so that legal obligations and ethical obligations are met.

- A "serious breach" means a breach likely to affect to a significant degree the safety and rights of a participant or the reliability and robustness of the data generated in the clinical trial.

AstraZeneca will comply with country-specific regulatory requirements relating to serious breach reporting to the regulatory authority, IRB/IEC, and Investigators.

- Where the EU Clinical Trials Regulation 536/2014 applies, AstraZeneca has in place processes to enter details of serious breaches into the European Medicines Agency CTIS. It is important to note that redacted versions of serious breach reports will be available to the public via CTIS.

If any (potential) serious breach occurs in the course of the study, Investigators or other site personnel will inform the appropriate AstraZeneca representatives immediately.

In certain regions/countries, AstraZeneca has a legal responsibility to notify both the local regulatory authority and other regulatory agencies about such breaches.

The investigator should have a process in place to ensure that:

- The site staff or service providers delegated by the investigator/institution are able to identify the occurrence of a (potential) serious breach.
- A (potential) serious breach is promptly reported to AstraZeneca or delegated party, through the contacts (e-mail address or telephone number) provided by AstraZeneca.

A 2 Financial Disclosure

Investigators and sub-investigators will provide the Sponsor with sufficient, accurate financial information as requested to allow the Sponsor to submit complete and accurate financial

certification or disclosure statements to the appropriate regulatory authorities. Investigators are responsible for providing information on financial interests during the course of the study and for 1 year after completion of the study.

A 3 Informed Consent Process

- The Investigator or his/her representative will explain the nature of the study to the participant or his/her legally authorized representative and answer all questions regarding the study.
- Participants or their legally authorized representative must be informed that their participation is voluntary, and they are free to refuse to participate and may withdraw their consent at any time and for any reason during the study. The participant's legally authorized representative will be required to sign a statement of informed consent that meets the requirements of 21 CFR 50, local regulations, ICH guidelines, Health Insurance Portability and Accountability Act requirements, where applicable, and the IRB/IEC or study center.
- The medical record must include a statement that written informed consent was obtained before the participant was enrolled in the study and the date the written consent was obtained. The authorized person obtaining the informed consent must also sign the ICF.
- Participants must be re-consented to the most current version of the ICF(s) during their participation in the study.
- A copy of the ICF(s) must be provided to the participant’s legally authorized representative.

Participants who are rescreened are required to sign a new ICF (or signed by their legally authorized representative).

A 4 Data Protection

- Participants will be assigned a unique identifier by the Sponsor. Any participant records or datasets that are transferred to the Sponsor will contain the identifier only; participant names or any information which would make the participant identifiable will not be transferred.
- The participant or their legally authorized representative must be informed that his/her personal study-related data will be used by the Sponsor in accordance with local data protection law. The level of disclosure and use of their data must also be explained to the participant or their legally authorized representative in the informed consent
- The participant or their legally authorized representative must be informed that his/her medical records may be examined by Clinical Quality Assurance auditors or other authorized personnel appointed by the Sponsor, by appropriate IRB/IEC members, and by inspectors from regulatory authorities.

- The participant must be informed that data will be collected only for the business needs. We will only collect and use the minimum amount of personal data to support our business activities and will not make personal data available to anyone (including internal staff) who is not authorized or does not have a business need to know the information.
- The participant must be informed that in some cases their data may be pseudonymized. The General Data Protection Regulation defines pseudonymization as the processing of personal data in such a way that the personal data can no longer be attributed to a specific individual without the use of additional information, provided that such additional information is kept separately and protected by technical and organizational measures to ensure that the personal data are not attributed to an identified or identifiable natural person.

Personal Data Breaches

A ‘personal data breach’ means a breach of security leading to the accidental or unlawful destruction, loss, alteration, unauthorized disclosure of, or access to, personal data transmitted, stored or otherwise processed.

- In compliance with applicable laws, the Data Controller[1] for the processing activity where the personal data breach occurred (AstraZeneca or respectively the site), will notify the data protection authorities without undue delay within the legal terms provided for such notification and within the prescribed form and content.

---

[1] The **data controller** determines the **purposes** for which and the **means** by which personal data are processed, as defined by the European Commission

- While AstraZeneca has processes in place to deal with personal data breaches it is important that investigators that work with AstraZeneca have controls in place to protect patient data privacy.

The Investigator should have a process in place to ensure that:

- allow site staff or service providers delegated by the investigator/institution to identify the occurrence of a (potential) personal data breaches.
- any (potential) personal data breach is promptly reported to AstraZeneca or delegated party, through the contacts (e-mail address or telephone number) provided by AstraZeneca.

AstraZeneca and the site must demonstrate that they:

- have taken all necessary steps to avoid personal data breaches and
- have undertaken measures to prevent such breaches from occurring in the first place and to mitigate the impact of occurred data breaches (eg, applying encryption, maintaining and keeping systems and IT security measures up-to-date, regular reviews and testing, regular training of employees, and developed security policies and standards).
- where possible, have developed an internal data breach reporting and investigation process and internal protocols with guidance on how to respond swiftly and diligently to the occurrence of a personal data breach.
- where it has not been possible to develop an internal data breach reporting and investigation process, the site follows AstraZeneca's instructions.

Notification of Personal Data Breach to Participants:

- Notification to participants is done by the site for the data breaches that occurred within the processing activities for which the site is the Data Controller and for data breaches occurred within the processing activities of AstraZeneca as the Data Controller, the notification is done in collaboration with the site and is performed by the site and/or Principal Investigator, acting on behalf of AstraZeneca, so that AstraZeneca has no access to the identifying personal information of the participants. The site and/or Principal Investigator shall conduct the notification by contacting the participants using the information that they gave for communication purposes in clinical research.
- If a personal data breach occurs in a processor's systems, engaged by AstraZeneca, the processor under contractual obligations with AstraZeneca promptly and in due course after discovering the breach notifies AstraZeneca and provides full cooperation with the investigation. In these cases, to the extent AstraZeneca is the Data Controller for the processing activity where the breach occurred, it will be responsible for the notification to data protection authorities and, if applicable, to participants. If the personal data breach needs to be notified to the participants, the notification to participants is done in

collaboration with the site and is performed by the site and/or Principal Investigator, acting on behalf of the Sponsor, so that AstraZeneca has no access to the identifying personal information of the participants.

- If a personal data breach involving an AstraZeneca's representative device (ie, Study Monitor laptop), AstraZeneca representative will provide AstraZeneca with all of the information needed for notification of the breach, without disclosing data that allows AstraZeneca directly or indirectly to identify the participants. The notification will be done by AstraZeneca solely with the information provided by the Study Monitor and in no event with access to information that could entail a risk of re-identification of the participants. If the data breach must be notified to the data subjects, the notification will be done directly by the Study Monitor in collaboration with the site and/or Principal Investigator, acting on behalf of the Sponsor, so that AstraZeneca has no access to the identifying personal information of the participants. The contract between AstraZeneca and the Study Monitor shall expressly specify these conditions.
- The contract between the site and AstraZeneca for performing the clinical research includes the provisions and rules regarding who is responsible for coordinating and directing the actions in relation to the breaches and performing the mandatory notifications to authorities and participants, where applicable.

A 5 Committees Structure

The safety of all Sponsor clinical studies is closely monitored on an ongoing basis by Sponsor representatives in consultation with Patient Safety. Issues identified will be addressed; for instance, this could involve amendments to the CSP and letters to investigators.

A 6 Dissemination of Clinical Study Data

Any results for this trial, including both technical and lay summaries, will be submitted to the EU CTIS within a year from the global End of Trial Date in all participating countries, due to scientific reasons, as otherwise statistical analysis is not relevant.

A description of this clinical study will be available on www.astrazenecaclinicaltrials.com as well as https://euclinicaltrials.eu and http://www.clinicaltrials.gov, as will the summary of the study results when they are available. The clinical study and/or summary of study results may also be available on other websites according to the regulations of the countries in which the study is conducted.

A 7 Data Quality Assurance

- All participant data relating to the study will be recorded on eCRF unless transmitted to the Sponsor or designee electronically (eg, laboratory data). The Investigator is

responsible for verifying that data entries are accurate and correct by electronically signing the eCRF.

- The Investigator must maintain accurate documentation (source data) that supports the information entered in the eCRF.
- The Investigator must permit study-related monitoring, audits, IRB/IEC review, and regulatory agency inspections and provide direct access to source data documents.
- Monitoring details describing strategy (eg, risk-based initiatives in operations and quality such as Risk Management and Mitigation Strategies and Analytical Risk-Based Monitoring), methods, responsibilities and requirements, including handling of noncompliance issues and monitoring techniques (central, remote, or on-site monitoring) are provided in the Monitoring Plan.
- The Sponsor or designee is responsible for the data management of this study including quality checking of the data.
- The Sponsor assumes accountability for actions delegated to other individuals (eg, Contract Research Organizations).
- Study monitors will perform ongoing source data verification to confirm that data entered into the eCRF by authorized site personnel are accurate, complete, and verifiable from source documents; that the safety and rights of participants are being protected; and that the study is being conducted in accordance with the currently approved protocol and any other study agreements, ICH GCP, and all applicable regulatory requirements.
- Records and documents, including signed ICFs, pertaining to the conduct of this study must be retained by the Investigator for a minimum of 25 years after study archiving or as required by local regulations, according to the AstraZeneca Global Retention and Disposal Schedule. No records may be destroyed during the retention period without the written approval of AstraZeneca. No records may be transferred to another location or party without written notification to AstraZeneca.

A 8 Source Documents

- Source documents provide evidence for the existence of the participant and substantiate the integrity of the data collected. Source documents are filed at the Investigator’s site.
- Data entered in the eCRF that are transcribed from source documents must be consistent with the source documents or the discrepancies must be explained. The Investigator may need to request previous medical records or transfer records, depending on the study. Also, current medical records must be available.

A 9 Study and Site Start and Closure

The study start date is the date on which the clinical study will be open for recruitment of participants.

The first act of recruitment is the first participant screened and will be the study start date.

The Sponsor designee reserves the right to close the study site or terminate the study at any time for any reason at the sole discretion of the Sponsor. Study sites will be closed upon study completion. A study site is considered closed when all required documents and study supplies have been collected and a study site closure visit has been performed.

The Investigator may initiate study site closure at any time, provided there is reasonable cause and sufficient notice is given in advance of the intended termination.

Reasons for the early closure of a study site by the Sponsor or Investigator may include but are not limited to:

- Failure of the Investigator to comply with the protocol, the requirements of the IRB/IEC or local health authorities, the Sponsor's procedures, or GCP guidelines
- Inadequate recruitment of participants by the Investigator
- Discontinuation of further study intervention development

If the study is prematurely terminated or suspended, the Sponsor shall promptly inform the investigators, the IECs/IRBs, the regulatory authorities, and any contract research organization(s) used in the study of the reason for termination or suspension, as specified by the applicable regulatory requirements. The Investigator shall promptly inform the participant and should assure appropriate participant therapy and/or follow-up.

Participants from terminated sites will have the opportunity to be transferred to another site to continue the study.

A 10 Publication Policy

- The results of this study may be published or presented at scientific meetings. If this is foreseen, the Investigator agrees to submit all manuscripts or abstracts to the Sponsor before submission. This allows the Sponsor to protect proprietary information and to provide comments.
- The Sponsor will comply with the requirements for publication of study results. In accordance with standard editorial and ethical practice, the Sponsor will generally support publication of multi-center studies only in their entirety and not as individual site data. In this case, a coordinating Investigator will be designated by mutual agreement.
- Authorship will be determined by mutual agreement and in line with International Committee of Medical Journal Editors authorship requirements.

Appendix B Adverse Events: Definitions and Procedures for Recording, Evaluating, Follow-up, and Reporting

B 1 Definition of Adverse Events

An AE is the development of any untoward medical occurrence in a patient or clinical study participant administered a medicinal product and which does not necessarily have a causal relationship with this treatment. An AE can therefore be any unfavorable and unintended sign (eg, an abnormal laboratory finding), symptom (eg, nausea, chest pain), or disease temporally associated with the use of a medicinal product, whether or not considered related to the medicinal product.

The term AE is used to include both serious and non-serious AEs and can include a deterioration of a pre-existing medical occurrence. An AE may occur at any time, including run-in or washout periods, even if no study intervention has been administered.

B 2 Definition of Serious Adverse Events

An SAE is an AE occurring during any study phase (ie, run-in, treatment, washout, follow-up), that fulfills one or more of the following criteria:

- Results in death
- Is immediately life-threatening
- Requires in-participant hospitalization or prolongation of existing hospitalization
- Results in persistent or significant disability or incapacity
- Is a congenital anomaly or birth defect
- Is an important medical event that may jeopardize the participant or may require medical treatment to prevent one of the outcomes listed above.

Adverse events for **malignant tumors** reported during a study should generally be assessed as **Serious AEs**. If no other seriousness criteria apply, the 'Important Medical Event' criterion should be used. In certain situations, however, medical judgment on an individual event basis should be applied to clarify that the malignant tumor event should be assessed and reported as a **non-serious AE**. For example, if the tumor is included as medical history and progression occurs during the study, but the progression does not change treatment and/or prognosis of the malignant tumor, the AE may not fulfill the attributes for being assessed as serious, although reporting of the progression of the malignant tumor as an AE is valid and should occur. Also, some types of malignant tumors, which do not spread remotely after a routine treatment that does not require hospitalization, may be assessed as non-serious; examples in adults include Stage 1 basal cell carcinoma and Stage 1A1 cervical cancer removed via cone biopsy.

Life-threatening

'Life-threatening' means that the participant was at immediate risk of death from the AE as it occurred or it is suspected that use or continued use of the product would result in the participant's death. 'Life-threatening' does not mean that had an AE occurred in a more severe form it might have caused death (eg, hepatitis that resolved without hepatic failure).

Hospitalization

Outpatient treatment in an emergency room is not in itself a serious AE, although the reasons for it may be (eg, bronchospasm, laryngeal edema). Hospital admissions and/or surgical operations planned before or during a study are not considered AEs if the illness or disease existed before the participant was enrolled in the study, provided that it did not deteriorate in an unexpected way during the study.

Important Medical Event or Medical Treatment

Medical and scientific judgment should be exercised in deciding whether a case is serious in situations where important medical events may not be immediately life threatening or result in death, hospitalization, disability or incapacity but may jeopardize the participant or may require medical treatment to prevent one or more outcomes listed in the definition of serious. These should usually be considered as serious.

Simply stopping the suspect drug does not mean that it is an important medical event; medical judgment must be used.

- Angioedema not severe enough to require intubation but requiring iv hydrocortisone treatment
- Hepatotoxicity caused by paracetamol (acetaminophen) overdose requiring treatment with N-acetylcysteine
- Intensive treatment in an emergency room or at home for allergic bronchospasm
- Blood dyscrasias (eg, neutropenia or anemia requiring blood transfusion, etc.) or convulsions that do not result in hospitalization
- Development of drug dependency or drug abuse

Intensity Rating Scale:

- Mild (awareness of sign or symptom, but easily tolerated)
- Moderate (discomfort sufficient to cause interference with normal activities)
- Severe (incapacitating, with inability to perform normal activities)

It is important to distinguish between serious and severe AEs. Severity is a measure of intensity whereas seriousness is defined by the criteria in Appendix B 2. An AE of severe

intensity need not necessarily be considered serious. For example, nausea that persists for several hours may be considered severe nausea, but not a SAE unless it meets the criteria shown in Appendix B 2. On the other hand, a stroke that results in only a limited degree of disability may be considered a mild stroke but would be a SAE when it satisfies the criteria shown in Appendix B 2.

B 3 A Guide to Interpreting the Causality Question

When making an assessment of causality consider the following factors when deciding if there is a 'reasonable possibility' that an AE may have been caused by the drug.

- Time Course. Exposure to suspect drug. Has the participant actually received the suspect drug? Did the AE occur in a reasonable temporal relationship to the administration of the suspect drug?
- Consistency with known drug profile. Was the AE consistent with the previous knowledge of the suspect drug (pharmacology and toxicology) or drugs of the same pharmacological class? Or could the AE be anticipated from its pharmacological properties?
- De-challenge experience. Did the AE resolve or improve on stopping or reducing the dose of the suspect drug?
- No alternative cause. The AE cannot be reasonably explained by another etiology such as the underlying disease, other drugs, other host or environmental factors.
- Re-challenge experience. Did the AE reoccur if the suspected drug was reintroduced after having been stopped? AstraZeneca would not normally recommend or support a re-challenge.
- Laboratory tests. A specific laboratory investigation (if performed) has confirmed the relationship.

In difficult cases, other factors could be considered such as:

- Is this a recognized feature of overdose of the drug?
- Is there a known mechanism?

Causality of 'related' is made if following a review of the relevant data, there is evidence for a 'reasonable possibility' of a causal relationship for the individual case. The expression 'reasonable possibility' of a causal relationship is meant to convey, in general, that there are facts (evidence) or arguments to suggest a causal relationship.

The causality assessment is performed based on the available data including enough information to make an informed judgment. With no available facts or arguments to suggest a causal relationship, the event(s) will be assessed as ‘not related’.

Causal relationship in cases where the DUS has deteriorated due to lack of effect should be classified as no reasonable possibility.

B 4 Medication Error, Drug Abuse, and Drug Misuse

Medication Error

For the purposes of this clinical study a medication error is an unintended failure or mistake in the treatment process for an AstraZeneca study intervention that either causes harm to the participant or has the potential to cause harm to the participant.

A medication error is not lack of efficacy of the drug, but rather a human or process related failure while the drug is in control of the study site staff or participant.

Medication error includes situations where an error.

- Occurred
- **Was identified and** intercepted before the participant received the drug
- Did not occur, but circumstances were recognized that could have led to an error

Examples of events to be reported in clinical studies as medication errors:

- Drug name confusion
- Dispensing error eg, medication prepared incorrectly, even if it was not actually given to the participant
- Drug not administered as indicated, for example, wrong route or wrong site of administration
- Drug not taken as indicated eg, tablet dissolved in water when it should be taken as a solid tablet
- Drug not stored as instructed eg, kept in the fridge when it should be at room temperature
- Wrong participant received the medication (excluding IRT/RTSM errors)
- Wrong drug administered to participant (excluding IRT/RTSM errors)

Examples of events that **do not** require reporting as medication errors in clinical studies:

- Errors related to or resulting from IRT/RTSM - including those which lead to one of the above listed events that would otherwise have been a medication error

- Participant accidentally missed drug dose(s) eg, forgot to take medication
- Accidental overdose (will be captured as an overdose)
- Participant failed to return unused medication or empty packaging
- Errors related to background and rescue medication, or standard-of-care medication in open label studies, even if an AstraZeneca product

Medication errors are not regarded as AEs, but AEs may occur as a consequence of a medication error.

Drug Abuse

For the purpose of this study, drug abuse is defined as the persistent or sporadic intentional, non-therapeutic excessive use of IMP or AstraZeneca NIMP for a perceived reward or desired non-therapeutic effect.

Any events of drug abuse, with or without associated AEs, are to be captured and forwarded to the data entry site using the Drug Abuse Report Form. This form should be used both if the drug abuse happened in a study participant or if the drug abuse involves a person not enrolled in the study (such as a relative of the study participant).

Examples of drug abuse include but are not limited to:

- The drug is used with the intent of getting a perceived reward (by the study participant or a person not enrolled in the study).
- The drug in the form of a tablet is crushed and injected or snorted with the intent of getting high.

Drug Misuse

Drug misuse is the intentional and inappropriate use (by a study participant) of IMP or AstraZeneca NIMP for medicinal purposes outside of the authorized product information, or for unauthorized IMPs or AstraZeneca NIMPs, outside the intended use as specified in the protocol and includes deliberate administration of the product by the wrong route.

Events of drug misuse, with or without associated AEs, are to be captured and forwarded to the data entry site using the Drug Misuse Report Form. This form should be used both if the drug misuse happened in a study participant or if the drug misuse regards a person not enrolled in the study (such as a relative of the study participant).

Examples of drug misuse include but are not limited to:

- The drug is used with the intention to cause an effect in another person.

- The drug is sold to other people for recreational purposes.
- The drug is used to facilitate assault in another person.
- The drug is deliberately administered by the wrong route.
- The drug is split in half because it is easier to swallow, when it is stated in the protocol that it must be swallowed whole.
- Only half the dose is taken because the study participant feels that he/she is feeling better when not taking the whole dose.
- Someone who is not enrolled in the study intentionally takes the drug.

Appendix C Handling of Human Biological Samples

C 1 Chain of Custody

A full chain of custody is maintained for all samples throughout their lifecycle.

The Investigator keeps full traceability of collected biological samples from the participants while in storage at the center until shipment or disposal (where appropriate) and records relevant processing information related to the samples while at site.

The sample receiver keeps full traceability of the samples while in storage and during use until used or disposed of or until further shipment and keeps record of receipt of arrival and onward shipment or disposal.

AstraZeneca or delegated representatives will keep oversight of the entire life cycle through internal procedures, monitoring of study sites, auditing or process checks, and contractual requirements of external laboratory providers

Samples retained for further use will be stored in the AstraZeneca-assigned biobanks or other sample archive facilities and will be tracked by the appropriate AstraZeneca Team during for the remainder of the sample life cycle.

C 2 Withdrawal of Informed Consent for Donated Biological Samples

If a participant withdraws consent to the use of donated biological samples, the samples will be disposed of/destroyed/repatriated, and the action documented. If samples are already analyzed, AstraZeneca is not obliged to destroy the results of this research.

Following withdrawal of consent for biological samples, further study participation should be considered in relation to the withdrawal processes outlined in the informed consent.

The Investigator:

- Ensures participant’s withdrawal of informed consent to the use of donated samples is highlighted immediately to AstraZeneca or delegate.
- Ensures that relevant human biological samples from that participant, if stored at the study site, are immediately identified, disposed of as appropriate, and the action documented.
- Ensures that the participant and AstraZeneca are informed about the sample disposal.

AstraZeneca ensures the organization(s) holding the samples is/are informed about the withdrawn consent immediately and that samples are disposed of or repatriated as appropriate, and the action is documented and study site is notified.

C 3 International Airline Transportation Association 6.2 Guidance Document

LABELING AND SHIPMENT OF BIOHAZARD SAMPLES

International Airline Transportation Association (IATA) (**https://www.iata.org/whatwedo/cargo/dgr/Pages/download.aspx**) classifies infectious substances into 3 categories: Category A, Category B or Exempt

Category A Infectious Substances are infectious substances in a form that, when exposure to it occurs, is capable of causing permanent disability, life-threatening or fatal disease in otherwise healthy humans or animals.
Category A Pathogens are, eg, Ebola, Lassa fever virus. Infectious substances meeting these criteria which cause disease in humans or both in humans and animals must be assigned to UN 2814. Infectious substances which cause disease only in animals must be assigned to UN 2900.

Category B Infectious Substances are infectious Substances that do not meet the criteria for inclusion in Category A. Category B pathogens are, eg, Hepatitis A, C, D, and E viruses. They are assigned the following UN number and proper shipping name:

- UN 3373 – Biological Substance, Category B
- are to be packed in accordance with UN 3373 and IATA 650

Exempt - Substances which do not contain infectious substances or substances which are unlikely to cause disease in humans or animals are not subject to these regulations unless they meet the criteria for inclusion in another class.

- Clinical study samples will fall into Category B or exempt under IATA regulations
- Clinical study samples will routinely be packed and transported at ambient temperature in IATA 650 compliant packaging (https://www.iata.org/whatwedo/cargo/dgr/Documents/DGR-60-EN-PI650.pdf).
- Biological samples transported in dry ice require additional dangerous goods specification for the dry ice content

Appendix D Optional Genomics Initiative Sample

D 1 Use/Analysis of DNA

- AstraZeneca intends to collect and store DNA for genetic research to explore how genetic variations may affect clinical parameters, risk and prognosis of diseases, and the response to medications. This genetic research may lead to better understanding of diseases, better diagnosis of diseases or other improvements in health care and to the discovery of new diagnostics, treatments or medications. Therefore, where local regulations and IRB/IEC allow, a blood sample will be collected for DNA analysis from consenting participants.
- This optional genetic research may consist of the analysis of the structure of the participant´s DNA, ie, the entire genome.
- The results of genetic analyses may be reported in a separate study summary.
- The Sponsor will store the DNA samples in a secure storage space with adequate measures to protect confidentiality.

D 2 Genetic Research Plan and Procedures

Selection of Genetic Research Population

- All participants will be asked to participate in this genetic research. Participation is voluntary and if a participant declines to participate there will be no penalty or loss of benefit. The participant will not be excluded from any aspect of the main study.

Inclusion Criteria

- For inclusion in this genetic research, participants must fulfill all of the inclusion criteria described in the main body of the CSP and: Provide informed consent for the Genomics Initiative sampling and analyses.

Exclusion Criteria

- Exclusion from this genetic research may be for any of the exclusion criteria specified in the main study or any of the following:

Withdrawal of Consent for Genetic Research

- Participants may withdraw from this genetic research at any time, independent of any decision concerning participation in other aspects of the main study. Voluntary withdrawal will not prejudice further treatment. Procedures for withdrawal are outlined in Section 7.2.

Collection of Samples for Genetic Research

- The blood sample for this genetic research will be obtained from the participants at Visit 3 after randomization. Although DNA is stable, early sample collection is preferred to avoid introducing bias through excluding participants who may withdraw due to an AE. If for any reason the sample is not drawn at Visit 3, it may be taken at any visit until the last study visit. Only one sample should be collected per participant for genetics during the study.

Coding and Storage of DNA Samples

- The processes adopted for the coding and storage of samples for genetic analysis are important to maintain participant confidentiality. Samples will be stored for a maximum of 15 years, from the date of last participant last visit, after which they will be destroyed. DNA is a finite resource that is used up during analyses. Samples will be stored and used until no further analyses are possible or the maximum storage time has been reached.
- An additional second code will be assigned to the sample either before or at the time of DNA extraction replacing the information on the sample tube. Thereafter, the sample will be identifiable only by the second, unique number. This number is used to identify the sample and corresponding data at the AstraZeneca genetics laboratories, or at the designated organization. No personal details identifying the individual will be available to any person (AstraZeneca employee or designated organizations working with the DNA).
- The link between the participant enrollment/randomization code and the second number will be maintained and stored in a secure environment, with restricted access at AstraZeneca or designated organizations. The link will be used to identify the relevant DNA samples for analysis, facilitate correlation of genotypic results with clinical data, allow regulatory audit, and permit tracing of samples for destruction in the case of withdrawal of consent.

Ethical and Regulatory Requirements

- The principles for ethical and regulatory requirements for the study, including this genetics research component, are outlined in Appendix A.

Informed Consent

- The genetic component of this study is optional and the participant may participate in other components of the main study without participating in this genetic component. To participate in the genetic component of the study the participant must sign and date both the consent form for the main study and the addendum for the Genomics Initiative component of the study. Copies of both signed and dated consent forms must be given to

the participant and the original filed at the study center. The PI(s) is responsible for ensuring that consent is given freely and that the participant understands that they may freely withdrawal from the genetic aspect of the study at any time.

Participant Data Protection

- AstraZeneca will not provide individual genotype results to participants, any insurance company, any employer, their family members, general physician unless required to do so by law.
- Extra precautions are taken to preserve confidentiality and prevent genetic data being linked to the identity of the participant. In exceptional circumstances, however, certain individuals might see both the genetic data and the personal identifiers of a participant. For example, in the case of a medical emergency, an AstraZeneca Physician or an Investigator might know a participant's identity and also have access to his or her genetic data. Regulatory authorities may require access to the relevant files, though the participant's medical information and the genetic files would remain physically separate.

Data Management

- Any genetic data generated in this study will be stored at a secure system at AstraZeneca and/or designated organizations to analyse the samples.
- AstraZeneca and its designated organizations may share summary results (such as genetic differences from groups of individuals with a disease) from this genetic research with other researchers, such as hospitals, academic organizations or health insurance companies. This can be done by placing the results in scientific databases, where they can be combined with the results of similar studies to learn even more about health and disease. The researchers can only use this information for health-related research purposes. Researchers may see summary results but they will not be able to see individual participant data or any personal identifiers.
- Some or all of the clinical datasets from the main study may be merged with the genetic data in a suitable secure environment separate from the clinical database.

Appendix E Medical Device AEs, ADEs, SAEs, SADEs, USADEs and Medical Device Deficiencies: Definitions and Procedures for Recording, Evaluating, Follow-up, and Reporting in Medical Device Studies

- The definitions and procedures detailed in this Appendix are in accordance with International Organization for Standardization 14155 and European MDR 2017/745 for clinical device research (if applicable).
- Both the Investigator and the Sponsor will comply with all local reporting requirements for medical devices.
- The detection and documentation procedures described in this protocol apply to all Sponsor medical devices provided for use in the study. See Section 6.1.2 for the list of Sponsor medical devices.

E 1 Definition of Medical Device AE and ADE

Medical Device AE and ADE Definition

- An AE is any untoward medical occurrence in a clinical study participant, users, or other persons, temporally associated with the use of study intervention, whether or not considered related to the investigational medical device. An AE can therefore be any unfavorable and unintended sign (including an abnormal laboratory finding), symptom, or disease (new or exacerbated) temporally associated with the use of an investigational medical device. This definition includes events related to the investigational medical device or comparator and events related to the procedures involved.
- An adverse device effect (ADE) is defined as an AE related to the use of an investigational medical device. This definition includes any AE resulting from insufficient or inadequate instructions for use, deployment, implantation, installation, or operation, or any malfunction of the investigational medical device as well as any event resulting from use error or from intentional misuse of the investigational medical device.

E 2 Definition of Medical Device SAE, SADE and USADE

A Medical Device SAE is an any serious adverse event that:

a. Led to death

b. Led to serious deterioration in the health of the participant, that either resulted in:

- A life-threatening illness or injury. The term "life-threatening" in the definition of "serious" refers to an event in which the participant was at risk of death at the time of the event. It does not refer to an event, which hypothetically might have caused death if it were more severe.
- A permanent impairment of a body structure or a body function.

- Inpatient or prolonged hospitalization. Planned hospitalization for a pre-existing condition, or a procedure required by the protocol, without serious deterioration in health, is not considered an SAE.
- Medical or surgical intervention to prevent life-threatening illness or injury or permanent impairment to a body structure or a body function.
- Chronic disease (MDR 2017/745).

c. Led to fetal distress, fetal death, or a congenital abnormality or birth defect

SADE Definition

- A SADE is defined as an adverse medical device effect that has resulted in any of the consequences characteristic of an SAE.
- Any Medical Device Deficiency that might have led to an SAE if appropriate action had not been taken, intervention had not occurred, or circumstances had been less fortunate.

Unanticipated SADE (USADE) Definition

- An USADE (also identified as UADE in United States Regulations 21 CFR 813.3), is defined as a serious adverse medical device effect that by its nature, incidence, severity, or outcome has not been identified in the current version of the risk analysis report (see Section 2.2).

E 3 Definition of Medical Device Deficiency

Medical Device Deficiency Definition

- A Medical Device Deficiency is an inadequacy of a medical device with respect to its identity, quality, durability, reliability, safety, or performance. Medical device deficiencies include malfunctions, use errors, and information supplied by the manufacturer.

E 4 Recording and Follow-up of AE and/or SAE and Medical Device Deficiencies

AE, SAE, and Medical Device Deficiency Recording

- When an AE/SAE/Medical Device Deficiency occurs, it is the responsibility of the Investigator to review all documentation (eg, hospital progress notes, laboratory reports, and diagnostics reports) related to the event.
- The Investigator will then record all relevant AE/SAE/Medical Device Deficiency information in the participant's medical records, in accordance with the Investigator's normal clinical practice and on the appropriate form.
- It is **not** acceptable for the Investigator to send photocopies of the participant's medical records in lieu of completion of the AE/SAE/Medical Device Deficiency form.
- There may be instances when copies of medical records for certain cases are requested by authorized representatives of AstraZeneca, a regulatory authority, or an EC/IRB official.

In this case, all participant identifiers, with the exception of the participant number, will be redacted on the copies of the medical records before submission.

- The Investigator will attempt to establish a diagnosis of the event based on signs, symptoms, and/or other clinical information. Whenever possible, the diagnosis (not the individual signs/symptoms) will be documented as the AE/SAE.
- For medical device deficiencies, it is very important that the Investigator describes any corrective or remedial actions taken to prevent recurrence of the deficiency.
    - A remedial action is any action other than routine maintenance or servicing of a medical device where such action is necessary to prevent recurrence of a Medical Device Deficiency. This includes any amendment to the medical device design to prevent recurrence.

Assessment of Intensity

The Investigator will make an assessment of intensity for each AE/SAE/Medical Device Deficiency reported during the study and assign it to one of the following categories:

- Mild: An event that is easily tolerated by the participant, causing minimal discomfort and not interfering with everyday activities.
- Moderate: An event that causes sufficient discomfort and interferes with normal everyday activities.
- Severe: An event that prevents normal everyday activities. An AE that is assessed as severe should not be confused with an SAE. "Severe" is a category used for rating the intensity of an event; both AEs and SAEs can be assessed as severe.
- An event is defined as 'serious' when it meets at least 1 of the predefined outcomes as described in the definition of an SAE, **not** when it is rated as severe.
-

Assessment of Causality

- The Investigator is obligated to assess the relationship between study intervention and each occurrence of each AE/SAE/Medical Device Deficiency.
- A "reasonable possibility" of a relationship conveys that there are facts, evidence, and/or arguments to suggest a causal relationship, rather than a relationship, cannot be ruled out.
- The Investigator will use clinical judgment to determine the relationship.
- Alternative causes, such as underlying disease(s), concomitant therapy, and other risk factors, as well as the temporal relationship of the event to study intervention administration will be considered and investigated.
- The Investigator will also consult the IB in his/her assessment.

- For each AE/SAE/Medical Device Deficiency, the Investigator **must** document in the medical notes that he/she has reviewed the AE/SAE/Medical Device Deficiency and has provided an assessment of causality.
- There may be situations in which an SAE has occurred and the Investigator has minimal information to include in the initial report. However, it is very important that the Investigator always make an assessment of causality for every event before the initial transmission of the SAE data.
- The Investigator may change his/her opinion of causality in light of follow-up information and send an SAE follow-up report with the updated causality assessment.
- The causality assessment is one of the criteria used when determining regulatory reporting requirements.

MDCG 2020 Guidance

For the purpose of harmonizing reports, each SAE will be classified according to 5 different levels of causality. The Sponsor and the investigators will use the following definitions to assess the relationship of the serious adverse event to the investigational[2] device or procedures.

1 Not related: Relationship to the device or procedures can be excluded when:

- the event has no temporal relationship with the use of the investigational device or the procedures;

2 Investigational device: any device object of the clinical investigation, including the comparators

- the serious event does not follow a known response pattern to the medical device (if the response pattern is previously known) and is biologically implausible;
- the discontinuation of medical device application or the reduction of the level of activation/exposure - when clinically feasible - and reintroduction of its use (or increase of the level of activation/exposure), do not impact on the serious event;
- the event involves a body-site or an organ not expected to be affected by the device or procedure;
- the serious event can be attributed to another cause (eg, an underlying or concurrent illness/clinical condition, an effect of another device, drug, treatment or other risk factors);
- the event does not depend on a false result given by the investigational device used for diagnosis, when applicable;

In order to establish the non-relatedness, not all the criteria listed above might be met at the same time, depending on the type of device/procedures and the serious event.

2 Unlikely: The relationship with the use of the device seems not relevant and/or the event can be reasonably explained by another cause, but additional information may be obtained.

3 Possible: The relationship with the use of the investigational device is weak but cannot be ruled out completely. Alternative causes are also possible (eg, an underlying or concurrent illness/ clinical condition or/and an effect of another device, drug or treatment). Cases were relatedness cannot be assessed or no information has been obtained should also be classified as possible.

4 Probable: The relationship with the use of the investigational device seems relevant and/or the event cannot be reasonably explained by another cause, but additional information may be obtained.

5 Causal relationship: the serious event is associated with the investigational device or with procedures beyond reasonable doubt when:

- the event is a known side effect of the product category the device belongs to or of similar devices and procedures;
- the event has a temporal relationship with investigational device use/application or procedures;
- the event involves a body-site or organ that
- the investigational device or procedures are applied to;
- the investigational device or procedures have an effect on;
- the serious event follows a known response pattern to the medical device (if the response pattern is previously known);

- the discontinuation of medical device application (or reduction of the level of activation/exposure) and reintroduction of its use (or increase of the level of activation/exposure), impact on the serious event (when clinically feasible);
- other possible causes (eg, an underlying or concurrent illness/ clinical condition or/and an effect of another device, drug or treatment) have been adequately ruled out;
- harm to the participant is due to error in use;
- the event depends on a false result given by the investigational device used for diagnosis, when applicable;

In order to establish the relatedness, not all the criteria listed above might be met at the same time, depending on the type of device/procedures and the serious event.

Follow-up of AE/SAE/Medical Device Deficiency

- The Investigator is obligated to perform or arrange for the conduct of supplemental measurements and/or evaluations as medically indicated or as requested to elucidate the nature and/or causality of the AE/SAE/Medical Device Deficiency as fully as possible. This may include additional laboratory tests or investigations, histopathological examinations, or consultation with other health care professionals.
- Suggested bullet in Variable blue text may not be required for studies where death is an endpoint.
- New or updated information will be recorded in the originally completed form.
- The Investigator will submit any updated SAE data within 24 hours of receipt of the information.

E 5 Reporting of SAEs

SAE Reporting via Paper Data Collection Tool

- Initial notification via telephone does not replace the need for the Investigator to complete and sign the SAE paper data collection tool within the designated reporting time frames.
- Contacts for SAE reporting can be found in latest IB.

E 6 Reporting of SADEs

SADE Reporting

NOTE: There are additional reporting obligations for medical device deficiencies that are potentially related to SAEs that must fulfill the legal responsibility to notify appropriate

regulatory authorities and other entities about certain safety information relating to medical devices being used in clinical studies.

- Any Medical Device Deficiency that is associated with an SAE must be reported to the Sponsor within 24 hours after the Investigator determines that the event meets the definition of a Medical Device Deficiency.
- The Sponsor will review all medical device deficiencies and determine and document in writing whether they could have led to an SAE. These medical device deficiencies will be reported to the regulatory authorities and IRBs/IECs as required by national regulations.
- Contacts for SAE reporting can be found in Section Appendix B.

Appendix F Changes Related to Mitigation of Study Disruptions Due to Cases of Civil Crisis, Natural Disaster, or Public Health Crisis

Note: Changes below should be implemented only during study disruptions due to any of or a combination of civil crisis, natural disaster, or public health crisis (eg, during quarantines and resulting site closures, regional travel restrictions and considerations if site personnel or study participants become infected with severe acute respiratory syndrome coronavirus 2 (SARS-CoV-2) or similar pandemic infection) during which participants may not wish to or may be unable to visit the study site for study visits. These changes should only be implemented if allowable by local/regional guidelines and following agreement from the Sponsor.

Reconsent of Study Participants During Study Interruptions

During study interruptions, it may not be possible for the participants to complete study visits and assessments on site and alternative means for carrying out the visits and assessments may be necessary, eg, remote visits. Reconsent should be obtained for the alternative means of carrying out visits and assessments and should be obtained prior to performing the procedures described in Section 8. Local and regional regulations and/or guidelines regarding reconsent of study participants should be checked and followed. Reconsent may be verbal if allowed by local and regional guidelines (note, in the case of verbal reconsent the ICF should be signed at the participant's next contact with the study site). Visiting the study sites for the sole purpose of obtaining reconsent should be avoided.

Rescreening of Participants to Reconfirm Study Eligibility

Additional rescreening for screen failure due to study disruption can be performed in previously screened participants. The Investigator should confirm this with the AstraZeneca Study Physician.

In addition, during study disruption there may be a delay between confirming eligibility of a participant and either enrollment into the study or commencing of dosing with study intervention.

If this delay is outside the screening window specified in the SoA Table 1, the participant will need to be rescreened to reconfirm eligibility before commencing study procedures. This will provide another opportunity to re-screen a participant in addition to that detailed in Section 5.5. The screening procedures detailed in Sections 1.3 and 5.5 must be undertaken to confirm eligibility using the same randomization number as for the participant.

Home or Remote Visit to Replace On-site Visit (Where Applicable)

A qualified HCP from the study site or TPV service may visit the participants' home/or other remote location as per local write in full if first use SOPs, as applicable.

Supplies will be provided for a safe and efficient visit. The qualified HCP will be expected to collect information per the CSP.

Telemedicine Visit to Replace On-site Visit (Where Applicable)

In this Appendix, the term telemedicine visit refers to remote contact with the participants using telecommunications technology including telephone calls, virtual or video visits, and mobile health devices.

During a civil crisis, natural disaster, or public health crisis, on-site visits may be replaced by a telemedicine visit if allowed by local/regional guidelines. Having a telemedicine contact with the participants will allow adverse events, concomitant medication, add other information including efficacy data where relevant to be collected according to study requirements to be reported and documented.

At-home or Remote Location Study Intervention Administration Instructions

If a site visit is not possible, at-home or remote location administration of study intervention may be performed by a qualified HCP, provided this is acceptable within local regulation/guidance, or by the participant or his/her caregiver. The option of at-home or remote location study intervention administration ensures participants safety in cases of a pandemic where participants may be at increased risk by traveling to the clinic. This will also minimize interruption of study intervention administration during other study disruptions, eg, site closures due to natural disaster.

At-home or Remote Location Study Intervention Administration by a Qualified HCP or TPV Service

A qualified HCP from the study site or TPV service may administer the study intervention at the participant's home or other remote location according to the CSP, and if allowed by local SOPs, as applicable. All necessary supplies and instructions for administration and documentation of study intervention administration will be provided. Additional information related to the visit can be obtained via a telemedicine or home visit.

Data Capture During Telemedicine or Home/Remote Visits

Data collected during telemedicine or home/remote visits will be captured by the qualified HCP from the study site or TPV service in the source documents, or by the participant themselves.

Appendix G Abbreviations

Abbreviation or special term	Explanation
AE	adverse event
AEOSI	adverse event of special interest
ATS	American Thoracic Society
AxMP	auxiliary medicinal product
BGF	budesonide, glycopyrronium, and formoterol fumarate
BID	twice daily (‘*bis in die’*)
CAT	COPD Assessment Test
CFC	Chlorofluorocarbons
CI	confidence interval
CID	clinically important deterioration
CKD-EPI	Chronic Kidney Disease Epidemiology Collaboration
COPD	chronic obstructive pulmonary disease
COVID-19	coronavirus disease 2019
CSP	Clinical Study Protocol
CSR	Clinical Study Report
CT	computed tomography
CTIS	Clinical Trials Information System
CTR	Clinical Trial Regulation
CYP3A4	cytochromeP450 3A4
DAE	discontinuation due to AE
dECG	digital electrocardiogram

Abbreviation or special term	Explanation
DPI	dry powder inhaler
DUS	Disease under Study
ECG	electrocardiogram
EDC	electronic data capture
eGFR	estimated glomerular filtration rate
ERS	European Respiratory Society
EU	European Union
FDA	US Food and Drug Administration
FEV1	forced expiratory volume in 1 second
FVC	forced vital capacity
GCP	Good Clinical Practice
GWP	global warming potential
hCG	(urine) human chorionic gonadotropin test
HFA	hydrofluoroalkane
HFC (HCFC)	hydrochlorofluorocarbons
HFO	hydrofluoroolefin
ICD	Informed consent documents
ICF	informed consent form
ICH	International Council for Harmonisation
ICS	inhaled corticosteroid
IFU	instruction for use
IMP	Investigational medicinal product

Abbreviation or special term	Explanation
IRT/RTSM	Interactive Response Technology/Randomization and Trial Supply Management
IxRS	Interactive Voice/Web Response System
LABA	long-acting $beta_2$-agonist
LAMA	long-acting muscarinic antagonist
MCID	minimal clinically significant difference
MDI	metered dose inhaler
MedDRA	Medical Dictionary for Regulatory Activities
NIMP	Non-Investigational medicinal product
NOAEL	No-Observable-Adverse-Effect-Level
NOEL	No-Observable-Effect-Level
pMDI	Pressurized metered-dose inhaler
PT	preferred term
QTcF	QT corrected for heart rate using Fridericia's formula
RoW	Rest of the World
SAE	serious adverse event
SABA	short-acting $beta_2$-agonist
SAMA	short-acting muscarinic antagonist
SARS-CoV-2	severe acute respiratory syndrome coronavirus 2
SD	standard deviation
SoA	Schedule of Activities
SOC	system organ class
US	United States of America

11 REFERENCES

Alexander et al 1995
Alexander, Dj, and Se Libretto. "An Overview of the Toxicology of HFA-134a (1,1,1,2-Tetrafluoroethane)." *Human & Experimental Toxicology* 14, no. 9 (September 1995): 715–20. https://doi.org/10.1177/096032719501400903.

Beasley et al 2019
Beasley, Richard, Mark Holliday, Helen K. Reddel, Irene Braithwaite, Stefan Ebmeier, Robert J. Hancox, Tim Harrison, et al. "Controlled Trial of Budesonide–Formoterol as Needed for Mild Asthma." New England Journal of Medicine 380, no. 21 (May 23, 2019): 2020–30. https://doi.org/10.1056/NEJMoa1901963.

Berger et al 2008
Berger, W.E., and J.A. Nadel. "Efficacy and Safety of Formoterol for the Treatment of Chronic Obstructive Pulmonary Disease." *Respiratory Medicine* 102, no. 2 (February 2008): 173–88. https://doi.org/10.1016/j.rmed.2007.09.011.

Buist et al 2007
Buist, A.S., McBurnie, M.A., Vollmer, W.M., Gillespie, S., Burney, P., Mannino, D.M., Menezes, A.M., Sullivan, S.D., Lee, T.A., Weiss, K.B., Jensen, R.L., Marks, G.B., Gulsvik, A., Nizankowska-Mogilnicka, E., 2007. International variation in the prevalence of COPD (The BOLD Study): a population-based prevalence study. The Lancet 370, 741–750. https://doi.org/10.1016/S0140-6736(07)61377-4

Celli et al 2004
Celli, B.R., W. MacNee, A. Agusti, A. Anzueto, B. Berg, A.S. Buist, P.M.A. Calverley, et al. "Standards for the Diagnosis and Treatment of Patients with COPD: A Summary of the ATS/ERS Position Paper." *European Respiratory Journal* 23, no. 6 (June 2004): 932–46. https://doi.org/10.1183/09031936.04.00014304.

Chanut et al 2013
Chanut, Franck, Carie Kimbrough, Rick Hailey, Brian Berridge, Angela Hughes-Earle, Rhiannon Davies, Kimberly Roland, et al. "Spontaneous Cardiomyopathy in Young Sprague-Dawley Rats: Evaluation of Biological and Environmental Variability." *Toxicologic Pathology* 41, no. 8 (December 2013): 1126–36. https://doi.org/10.1177/0192623313478692.

Feenstra et al 2001
Feenstra, Talitha L., Marianne L. L. van GENUGTEN, Rudolf T. Hoogenveen, Emiel F. Wouters, and Maureen P. M. H. RUTTEN-van MÖLKEN. "The Impact of Aging and Smoking on the Future Burden of Chronic Obstructive Pulmonary Disease: A Model Analysis in the Netherlands." *American Journal of Respiratory and Critical Care Medicine* 164, no. 4 (August 15, 2001): 590–96. https://doi.org/10.1164/ajrccm.164.4.2003167.

Ford et al 2013
Ford, Earl S., Janet B. Croft, David M. Mannino, Anne G. Wheaton, Xingyou Zhang, and Wayne H. Giles. "COPD Surveillance—United States, 1999-2011." *Chest* 144, no. 1 (July 2013): 284–305. https://doi.org/10.1378/chest.13-0809.

Ford et al 2015
Ford, Earl S., Louise B. Murphy, Olga Khavjou, Wayne H. Giles, James B. Holt, and Janet B. Croft. "Total and State-Specific Medical and Absenteeism Costs of COPD Among Adults Aged 18 Years in the United States for 2010 and Projections Through 2020." *Chest* 147, no. 1 (January 2015): 31–45. https://doi.org/10.1378/chest.14-0972.

Halpin et al 2017
Halpin, David M.G., Marc Decramer, Bartolome R. Celli, Achim Mueller, Norbert Metzdorf, and Donald P. Tashkin. "Effect of a Single Exacerbation on Decline in Lung Function in COPD." *Respiratory Medicine* 128 (July 2017): 85–91. https://doi.org/10.1016/j.rmed.2017.04.013.

Hatcher et al 2003
Hatcher RA, Trussell J, Nelson AL, Cates W Jr, Stewart F, Kowal D, eds. Contraceptive technology. 19th edition. New York: Ardent Media, 2007(a): 24. Table 3-2.Jackson, H., 2003. Detecting chronic obstructive pulmonary disease using peak flow rate: cross sectional survey. BMJ 327, 653–654. https://doi.org/10.1136/bmj.327.7416.653

Jackson 2003
Jackson, H. "Detecting Chronic Obstructive Pulmonary Disease Using Peak Flow Rate: Cross Sectional Survey." BMJ 327, no. 7416 (September 20, 2003): 653–54. https://doi.org/10.1136/bmj.327.7416.653.

Jones 2009
Jones, P.W., Harding, G., Berry, P., Wiklund, I., Chen, W.-H., Kline Leidy, N., 2009. Development and first validation of the COPD Assessment Test. European Respiratory Journal 34, 648–654. https://doi.org/10.1183/09031936.00102509.

Leach 2005
Leach, CL. The CFC to HFA transition and its impact on pulmonary drug development. Respir. Care 2005; 50(9):1201-1208.

Levey 2009
Levey, A.S., Stevens, L.A., Schmid, C.H., Zhang, Y. (Lucy), Castro, A.F., Feldman, H.I., Kusek, J.W., Eggers, P., Van Lente, F., Greene, T., Coresh, J., for the CKD-EPI (Chronic Kidney Disease Epidemiology Collaboration), 2009. A New Equation to Estimate Glomerular Filtration Rate. Annals of Internal Medicine 150, 604. https://doi.org/10.7326/0003-4819-150-9-200905050-00006

Levy 2015
Levy, Mark L. "The National Review of Asthma Deaths: What Did We Learn and What Needs to Change?" *Breathe* 11, no. 1 (March 2015): 14–24. https://doi.org/10.1183/20734735.008914.

Louis et al 2022
Louis, Renaud, Imran Satia, Inigo Ojanguren, Florence Schleich, Matteo Bonini, Thomy Tonia, David Rigau, et al. "European Respiratory Society Guidelines for the Diagnosis of Asthma in Adults." *European Respiratory Journal*, February 15, 2022, 2101585. https://doi.org/10.1183/13993003.01585-2021.

Mullin et al 1979
Mullin, L.S., C.F. Reinhardt, and R.E. Hemingway. "Cardiac Arrhythmias and Blood Levels Associated with Inhalation of Halon 1301." *American Industrial Hygiene Association Journal* 40, no. 7 (July 1979): 653–58. https://doi.org/10.1080/15298667991430118.

Naline et al 2007
Naline, E., Trifilieff, A., Fairhurst, R.A., Advenier, C., Molimard, M., 2007. Effect of indacaterol, a novel long-acting 2-agonist, on isolated human bronchi. European Respiratory Journal 29, 575–581. https://doi.org/10.1183/09031936.00032806

O'Byrne et al 2017
O'Byrne, Paul M., Christine Jenkins, and Eric D. Bateman. "The Paradoxes of Asthma Management: Time for a New Approach?" *European Respiratory Journal* 50, no. 3 (September 2017): 1701103. https://doi.org/10.1183/13993003.01103-2017.

Pritchard 2020
Pritchard, John N. "The Climate Is Changing for Metered-Dose Inhalers and Action Is Needed." *Drug Design, Development and Therapy* Volume 14 (July 2020): 3043–55. https://doi.org/10.2147/DDDT.S262141.

Quanjer 2012
Quanjer, P.H., Stanojevic, S., Cole, T.J., Baur, X., Hall, G.L., Culver, B.H., Enright, P.L., Hankinson, J.L., Ip, M.S.M., Zheng, J., Stocks, J., the ERS Global Lung Function Initiative, 2012. Multi-ethnic reference values for spirometry for the 3–95-yr age range: the global lung function 2012 equations. European Respiratory Journal 40, 1324–1343. https://doi.org/10.1183/09031936.00080312.

Roche et al 2016
Roche, Nicolas, and P.N. Richard Dekhuijzen. "The Evolution of Pressurized Metered-Dose Inhalers from Early to Modern Devices." *Journal of Aerosol Medicine and Pulmonary Drug Delivery* 29, no. 4 (August 2016): 311–27. https://doi.org/10.1089/jamp.2015.1232.

Rusch 2018
Rusch, George M. "The Development of Environmentally Acceptable Fluorocarbons." *Critical Reviews in Toxicology* 48, no. 8 (September 14, 2018): 615–65. https://doi.org/10.1080/10408444.2018.1504276.

Sastre et al 2016
Sastre, Joaquin, Leonardo M. Fabbri, David Price, Hans Ulrich Wahn, Jean Bousquet, James E. Fish, Kevin Murphy, and Malcolm R. Sears. "Insights, Attitudes, and Perceptions about Asthma and Its Treatment: A Multinational Survey of Patients from Europe and Canada." *World Allergy Organization Journal* 9 (2016): 13. https://doi.org/10.1186/s40413-016-0105-4.

Schreiber et al 2020
Schreiber, Jens, Tina Sonnenburg, and Eva Luecke. "Inhaler Devices in Asthma and COPD Patients – a Prospective Cross-Sectional Study on Inhaler Preferences and Error Rates." *BMC Pulmonary Medicine* 20, no. 1 (December 2020). https://doi.org/10.1186/s12890-020-01246-z.

Schuster et al 2009
Schuster, Paul, Rüdiger Bertermann, George M. Rusch, and Wolfgang Dekant. "Biotransformation of Trans-1,1,1,3-Tetrafluoropropene (HFO-1234ze)." *Toxicology and Applied Pharmacology* 239, no. 3 (September 15, 2009): 215–23. https://doi.org/10.1016/j.taap.2009.06.018.

Sellers 2017
Sellers, William F. S. "Asthma Pressurised Metered-Dose Inhaler Performance: Propellant Effect Studies in Delivery Systems." *Allergy, Asthma & Clinical Immunology* 13, no. 1 (December 2017). https://doi.org/10.1186/s13223-017-0202-0.

Suissa et al 2012
Suissa, Samy, Sophie Dell'Aniello, and Pierre Ernst. "Long-Term Natural History of Chronic Obstructive Pulmonary Disease: Severe Exacerbations and Mortality." *Thorax* 67, no. 11 (November 2012): 957–63. https://doi.org/10.1136/thoraxjnl-2011-201518.

Ulm 1990
Ulm, K. "SIMPLE METHOD TO CALCULATE THE CONFIDENCE INTERVAL OF A STANDARDIZED MORTALITY RATIO (SMR)." *American Journal of Epidemiology* 131, no. 2 (February 1990): 373–75. https://doi.org/10.1093/oxfordjournals.aje.a115507.

Usmani et al 2019
Usmani, Omar S, Jane Scullion, and Duncan Keeley. "Our Planet or Our Patients—Is the Sky the Limit for Inhaler Choice?" *The Lancet Respiratory Medicine* 7, no. 1 (January 2019): 11–13. https://doi.org/10.1016/S2213-2600(18)30497-1.

Wheaton et al 2019
Wheaton AG, Liu Y, Croft JB, et al. Chronic Obstructive Pulmonary Disease and Smoking Status — United States, 2017. *MMWR Morb Mortal Wkly Rep* 2019;68:533–538. DOI: http://dx.doi.org/10.15585/mmwr.mm6824a1

Wilkinson et al 2019
Wilkinson, Alexander J K, Rory Braggins, Ingeborg Steinbach, and James Smith. "Costs of Switching to Low Global Warming Potential Inhalers. An Economic and Carbon Footprint Analysis of NHS Prescription Data in England." *BMJ Open* 9, no. 10 (October 2019): e028763. https://doi.org/10.1136/bmjopen-2018-028763.

CPMP/ICH/375/95
ICH Topic E 1. Population Exposure: The Extent of Population. Exposure to Assess Clinical Safety. June 1995

Global strategy for the diagnosis, management, and prevention of COPD. Global Initiative for Chronic Obstructive Lung Disease (GOLD) 2021. https://goldcopd.org/

Workplace Environmental Exposure Level (WEEL). 2012. Workplace Environmental Exposure Level Guide: Difluoroethane. Fairfax (VA): American Industrial Hygiene Association

https://www.cdc.gov/dotw/copd/index.html

https://www.who.int/news-room/fact-sheets/detail/the-top-10-causes-of-death

https://www.thoracic.org/professionals/clinical-resources/asthma-center/guidelines.php

https://www.med.unc.edu/medicine/wp-content/uploads/sites/945/2018/12/inhaler-picture-guide.pdf

http://www.honeywell-solsticelba.com/wp-content/uploads/2011/09/low-GWP-blowing-agent-Honeywell-nextgeneration-paper-2011.pdf

SIGNATURE PAGE

This is a representation of an electronic record that was signed electronically and this page is the manifestation of the electronic signature

**Document Name:** d5985c00003-csp-v2		
**Document Title:**	D5985C00003 Clinical Study Protocol version 2	
**Document ID:**	Doc ID-004816084	
**Version Label:**	2.0 CURRENT LATEST APPROVED	
**Server Date (dd-MMM-yyyy HH:mm 'UTC'Z)**	**Signed by**	**Meaning of Signature**
14-Dec-2023 15:28 UTC	PPD	Content Approval
14-Dec-2023 15:56 UTC	PPD	Content Approval
14-Dec-2023 19:21 UTC	PPD	Content Approval
15-Dec-2023 13:28 UTC	PPD	Content Approval

Notes: (1) Document details as stored in ANGEL, an AstraZeneca document management system.
