# Supplementary material for: Safety of budesonide/glycopyrronium/formoterol fumarate dihydrate delivered by HFO-1234ze versus HFA-134a in chronic obstructive pulmonary disease: a phase 3, multi-site, randomised, double-blind, parallel-group, active-comparator study
Source: eClinicalMedicine. 2025 Aug 12;87:103402. doi: 10.1016/j.eclinm.2025.103402 (PMC12359160; doi:10.1016/j.eclinm.2025.103402)
Supplement: Supplementary Tables and Fig [file mmc2.pdf]

Supplementary material

Participant inclusion and exclusion criteria

Key inclusion criteria were as follows: aged 40–80 years (inclusive); a documented history of physician-diagnosed chronic obstructive pulmonary disease (COPD), as defined by the American Thoracic Society/European Respiratory Society[1] or local guidelines; regular use of dual (inhaled corticosteroid [ICS]/long-acting β_2-agonist [LABA] or long-acting muscarinic antagonist [LAMA]/LABA) or triple (ICS/LAMA/LABA; open or fixed-dose combinations) inhaled COPD maintenance therapies for at least 6 weeks before screening; a pre-bronchodilator forced expiratory volume in 1 second (FEV_1) of <80% predicted normal at visit 1 (screening); a post-bronchodilator FEV_1/forced vital capacity (FVC) ratio of <0.70 and post-bronchodilator FEV_1 of ≥25% to <80% predicted normal at visit 2 (screening); a COPD Assessment Test (CAT) score ≥10 at visit 1 (screening); a current/former smoker with a history of ≥10 pack-years of tobacco smoking; and acceptable pressurised metered dose inhalers (pMDI) administration and spirometry techniques. Female participants were not of childbearing potential or were using a form of highly effective birth control and were willing to remain on the birth control until at least 14 days after the last dose of study intervention.

Key exclusion criteria were as follows: a documented history of physician-diagnosed asthma within 5 years of visit 1 (in the opinion of the investigator) based on reviews of medical history and medical records; COPD due to alpha-1 antitrypsin deficiency or imminent life-threatening COPD (e.g., need for mechanical ventilation); historical or current evidence of clinically significant disease (e.g., cardiovascular, hepatic, renal, haematological, neurological, endocrine or gastrointestinal) or other respiratory disorders (e.g., known active tuberculosis, lung cancer, cystic fibrosis, significant bronchiectasis, immune deficiency disorders, severe neurological disorders affecting upper airway control, sarcoidosis, idiopathic interstitial pulmonary fibrosis, primary pulmonary hypertension, or pulmonary thromboembolic disease); sleep apnoea that, in the opinion of the investigator, could not be controlled; moderate or severe COPD exacerbations or respiratory infections ending within 4 weeks before visit 1 or during screening; severe acute respiratory syndrome coronavirus 2 (SARS-CoV-2) infection in the 8 weeks before visit 1 or during screening, or that required hospitalisation before visit 1 or during screening; pulmonary resection or lung volume reduction surgery during the 26 weeks (6

months) before visit 1; receipt of long-term oxygen therapy; significant or unstable relevant cardiovascular disorder (e.g., ischaemic heart disease, arrhythmia, cardiomyopathy, heart failure, uncontrolled hypertension) as defined/judged by the investigator; narrow-angle glaucoma not adequately treated or a change in vision that may have been relevant in the opinion of the investigator; unable to withhold short-acting bronchodilators for 6 hours before lung function testing at each applicable study visit; clinically significant symptomatic prostatic hypertrophy or bladder neck obstruction/urinary retention; trans-urethral resection of prostate or full resection of the prostate within 26 weeks (6 months) prior to visit 1; unresectable cancer that had not been in complete remission for at least 5 years prior to visit 1; known hypersensitivity to β_2-agonists, muscarinic antagonists or corticosteroids, or any component of the pMDI; clinically relevant abnormal findings in physical examination, clinical chemistry, haematology, vital signs, or electrocardiogram; and currently pregnant, breastfeeding, or planning pregnancy.

Country	Centre no.	Name and address of IEC/IRB	Chairman of IEC/IRB	Date of Approval
Argentina	201	Comité de Ética en Investigación de InAER, Arenales 3146 1 B, Buenos Aires, CABA 1425, Argentina	Carlos Cassini	17-Aug-2022
Argentina	202	Comité de Ética en Investigación de InAER, Arenales 3146 1 B, Buenos Aires, CABA 1425, Argentina	Carlos Cassini	28-Nov-2022
Argentina	203	IRB RESPIRE, Sarmiento 1152 1°piso, Buenos Aires, 1644 Argentina	Leonardo Perelis	29-Dec-2022
Argentina	204	EC Comité de Etica e Investigación, Vicente Lopez 1441, Quilmes, Buenos Aires B1878DVB, Argentina	Damian Percio	29-Nov-2022
Argentina	205	Comité de Ética Independiente – Consultorios Integrados, Italia 424, Rosario, Santa Fe 2000, Argentina	Javier Bilbao	24-Nov-2022
Bulgaria	901	Ethic Committee for Clinical Trials, 8, Damyan Gruev, Str, 1303 Sofia, Bulgaria	Assoc. prof. Maria Staevska-Kotasheva	30-Sep-2022
Bulgaria	902	Ethic Committee for Clinical Trials, 8, Damyan Gruev, Str, 1303 Sofia, Bulgaria	Assoc. prof. Maria Staevska-Kotasheva	26-Jul-2022
Bulgaria	903	Ethic Committee for Clinical Trials, 8, Damyan Gruev, Str, 1303 Sofia, Bulgaria	Assoc. prof. Maria Staevska-Kotasheva	02-Nov-2022
Bulgaria	904	Ethic Committee for Clinical Trials, 8, Damyan Gruev, Str, 1303 Sofia, Bulgaria	Assoc. prof. Maria Staevska-Kotasheva	30-Sep-2022
Bulgaria	905	Ethic Committee for Clinical Trials, 8, Damyan Gruev, Str, 1303 Sofia, Bulgaria	Assoc. prof. Maria Staevska-Kotasheva	30-Sep-2022
Bulgaria	906	Ethic Committee for Clinical Trials, 8, Damyan Gruev, Str, 1303 Sofia, Bulgaria	Assoc. prof. Maria Staevska-Kotasheva	30-Sep-2022
Bulgaria	907	Ethic Committee for Clinical Trials, 8, Damyan Gruev, Str, 1303 Sofia, Bulgaria	Assoc. prof. Maria Staevska-Kotasheva	29-Dec-2022
Bulgaria	908	Ethic Committee for Clinical Trials, 8, Damyan Gruev, Str, 1303 Sofia, Bulgaria	Assoc. prof. Maria Staevska-Kotasheva	29-Dec-2022
Bulgaria	909	Ethic Committee for Clinical Trials, 8, Damyan Gruev, Str, 1303 Sofia, Bulgaria	Assoc. prof. Maria Staevska-Kotasheva	29-Dec-2022
Canada	1001	Advarra, 372 Hollandview Trail, Suite 300, Aurora, ON L4G 0A5, Canada	Susan Ebert	18-Aug-2022
Canada	1002	Institut univ cardiologie et de pneumologie de Quebec, 2725 Chemin Ste-Foy, Quebec, QC G1V 4G5, Canada	Jamila Chakir	07-Oct-2022
Canada	1003	Advarra, 372 Hollandview Trail, Suite 300, Aurora, ON L4G 0A5, Canada	Susan Ebert	18-Aug-2022
Canada	1004	Advarra, 372 Hollandview Trail, Suite 300, Aurora, ON L4G 0A5, Canada	Susan Ebert	31-Aug-2022
Canada	1005	Advarra, 372 Hollandview Trail, Suite 300, Aurora, ON L4G 0A5, Canada	Susan Ebert	08-Sep-2022
Canada	1008	Advarra, 372 Hollandview Trail, Suite 300, Aurora, ON L4G 0A5, Canada	Susan Ebert	18-Oct-2022
Canada	1009	Advarra, 372 Hollandview Trail, Suite 300, Aurora, ON L4G 0A5, Canada	Susan Ebert	13-Oct-2022

Canada	1010	Advarra, 372 Hollandview Trail, Suite 300, Aurora, ON L4G 0A5, Canada	Susan Ebert	15-Aug-2022
Canada	1011	Advarra, 372 Hollandview Trail, Suite 300, Aurora, ON L4G 0A5, Canada	Susan Ebert	23-Sep-2022
Canada	1012	Advarra, 372 Hollandview Trail, Suite 300, Aurora, ON L4G 0A5, Canada	Susan Ebert	17-Nov-2022
Germany	2601	Ethikkommission bei der Landesärztekammer Niedersachsen Unterkommission zur Beurteilung medizinischer Forschung am Menschen, Karl Wiechert-Allee 18-22, Hannover 30625, Germany	Andreas Creutzig	24-Aug-2022
Germany	2602	Ethikkommission bei der Landesärztekammer Niedersachsen Unterkommission zur Beurteilung medizinischer Forschung am Menschen, Karl-Wiechert-Allee 18-22, Hannover 30625, Germany	Andreas Creutzig	24-Aug-2022
Germany	2603	Ethikkommission bei der Landesärztekammer Niedersachsen Unterkommission zur Beurteilung medizinischer Forschung am Menschen, Karl-Wiechert-Allee 18-22, Hannover 30625, Germany	Andreas Creutzig	24-Aug-2022
Germany	2604	Ethikkommission bei der Landesärztekammer Niedersachsen Unterkommission zur Beurteilung medizinischer Forschung am Menschen, Karl-Wiechert-Allee 18-22, Hannover 30625, Germany	Andreas Creutzig	24-Aug-2022
Germany	2605	Ethikkommission bei der Landesärztekammer Niedersachsen Unterkommission zur Beurteilung medizinischer Forschung am Menschen, Karl-Wiechert-Allee 18-22, Hannover 30625, Germany	Andreas Creutzig	24-Aug-2022
Germany	2606	Ethikkommission bei der Landesärztekammer Niedersachsen Unterkommission zur Beurteilung medizinischer Forschung am Menschen, Karl-Wiechert-Allee 18-22, Hannover 30625, Germany	Andreas Creutzig	24-Aug-2022
Germany	2607	Ethikkommission bei der Landesärztekammer Niedersachsen Unterkommission zur Beurteilung medizinischer Forschung am Menschen, Karl-Wiechert-Allee 18-22, Hannover 30625, Germany	Andreas Creutzig	24-Aug-2022
Germany	2608	Ethikkommission bei der Landesärztekammer Niedersachsen Unterkommission zur Beurteilung medizinischer Forschung am Menschen, Karl-Wiechert-Allee 18-22, Hannover 30625, Germany	Andreas Creutzig	24-Aug-2022
Germany	2609	Ethikkommission bei der Landesärztekammer Niedersachsen Unterkommission zur Beurteilung medizinischer Forschung am Menschen, Karl-Wiechert-Allee 18-22, Hannover 30625, Germany	Andreas Creutzig	24-Aug-2022
Germany	2610	Ethikkommission bei der Landesärztekammer Niedersachsen Unterkommission zur Beurteilung medizinischer Forschung am Menschen, Karl-Wiechert-Allee 18-22, Hannover 30625, Germany	Andreas Creutzig	24-Aug-2022

Germany	2611	Ethikkommission bei der Landesärztekammer Niedersachsen Unterkommission zur Beurteilung medizinischer Forschung am Menschen, Karl-Wiechert-Allee 18-22, Hannover 30625, Germany	Andreas Creutzig	24-Aug-2022
Germany	2612	Ethikkommission bei der Landesärztekammer Niedersachsen Unterkommission zur Beurteilung medizinischer Forschung am Menschen, Karl-Wiechert-Allee 18-22, Hannover 30625, Germany	Andreas Creutzig	24-Aug-2022
Germany	2613	Ethikkommission bei der Landesärztekammer Niedersachsen Unterkommission zur Beurteilung medizinischer Forschung am Menschen, Karl-Wiechert-Allee 18-22, Hannover 30625, Germany	Andreas Creutzig	24-Aug-2022
Germany	2614	Ethikkommission bei der Landesärztekammer Niedersachsen Unterkommission zur Beurteilung medizinischer Forschung am Menschen, Karl-Wiechert-Allee 18-22, Hannover 30625, Germany	Andreas Creutzig	24-Aug-2022
Germany	2615	Ethikkommission bei der Landesärztekammer Niedersachsen Unterkommission zur Beurteilung medizinischer Forschung am Menschen, Karl-Wiechert-Allee 18-22, Hannover 30625, Germany	Andreas Creutzig	24-Aug-2022
Germany	2616	Ethikkommission bei der Landesärztekammer Niedersachsen Unterkommission zur Beurteilung medizinischer Forschung am Menschen, Karl-Wiechert-Allee 18-22, Hannover 30625, Germany	Andreas Creutzig	24-Aug-2022
Germany	2617	Ethikkommission bei der Landesärztekammer Niedersachsen Unterkommission zur Beurteilung medizinischer Forschung am Menschen, Karl-Wiechert-Allee 18-22, Hannover 30625, Germany	Andreas Creutzig	24-Aug-2022
Germany	2618	Ethikkommission bei der Landesärztekammer Niedersachsen Unterkommission zur Beurteilung medizinischer Forschung am Menschen, Karl-Wiechert-Allee 18-22, Hannover 30625, Germany	Andreas Creutzig	24-Aug-2022
Germany	2619	Ethikkommission bei der Landesärztekammer Niedersachsen Unterkommission zur Beurteilung medizinischer Forschung am Menschen, Karl-Wiechert-Allee 18-22, Hannover 30625, Germany	Andreas Creutzig	24-Aug-2022
Germany	2620	Ethikkommission bei der Landesärztekammer Niedersachsen Unterkommission zur Beurteilung medizinischer Forschung am Menschen, Karl-Wiechert-Allee 18-22, Hannover 30625, Germany	Andreas Creutzig	24-Aug-2022
Mexico	4903	Comite de Etica en Investigación del Centro de Investigación Farmacéutica Especializada de Occidente S.C. Av. Vallarta No. 1670 Piso 2 PH1, Colonia Americana, Guadalajara, Jalisco, 44160	Dr. Lorenza Ibarrarán Álvarez	23-Aug-2022
Mexico	4905	Comite de Etica en Investigación del Centro de Investigación Farmacéutica Especializada de	Dr. Lorenza Ibarrarán Álvarez	18-Aug-2022

		Occidente S.C. Av. Vallarta No. 1670 Piso 2 PH1, Colonia Americana, Guadalajara, Jalisco, 44160		
Mexico	4906	Comite de Etica en Investigación del Centro de Investigación Farmacéutica Especializada de Occidente S.C. Av. Vallarta No. 1670 Piso 2 PH1, Colonia Americana, Guadalajara, Jalisco, 44160	Dr. Lorenza Ibarrarán Álvarez	23-Aug-2022
Mexico	4907	Comite de Etica en Investigación del Centro de Investigación Farmacéutica Especializada de Occidente S.C. Av. Vallarta No. 1670 Piso 2 PH1, Colonia Americana, Guadalajara, Jalisco, 44160	Dr. Lorenza Ibarrarán Álvarez	11-Jul-2022
Mexico	4910	Comite de Etica en Investigación del Centro de Investigación Farmacéutica Especializada de Occidente S.C. Av. Vallarta No. 1670 Piso 2 PH1, Colonia Americana, Guadalajara, Jalisco, 44160	Dr. Lorenza Ibarrarán Álvarez	11-Jul-2022
Mexico	4911	Comite de Etica en Investigación del Centro de Investigación Farmacéutica Especializada de Occidente S.C. Av. Vallarta No. 1670 Piso 2 PH1, Colonia Americana, Guadalajara, Jalisco, 44160	Dr. Lorenza Ibarrarán Álvarez	11-Jul-2022
Poland	5702	Komisja Bioetyczna przy Okręgowej Izbie Lekarskiej w Łodzi, ul. Czerwona 3, Łódź 93-005, Poland	Prof. dr hab. n. prawnych Urszula Promińska	05-Oct-2022
Poland	5703	Komisja Bioetyczna przy Okręgowej Izbie Lekarskiej w Łodzi, ul. Czerwona 3, Łódź 93-005, Poland	Prof. dr hab. n. prawnych Urszula Promińska	05-Oct-2022
Poland	5705	Komisja Bioetyczna przy Okręgowej Izbie Lekarskiej w Łodzi, ul. Czerwona 3, Łódź 93-005, Poland	Prof. dr hab. n. prawnych Urszula Promińska	05-Oct-2022
Poland	5706	Komisja Bioetyczna przy Okręgowej Izbie Lekarskiej w Łodzi, ul. Czerwona 3, Łódź 93-005, Poland	Prof. dr hab. n. prawnych Urszula Promińska	05-Oct-2022
Poland	5707	Komisja Bioetyczna przy Okręgowej Izbie Lekarskiej w Łodzi, ul. Czerwona 3, Łódź 93-005, Poland	Prof. dr hab. n. prawnych Urszula Promińska	05-Oct-2022
Poland	5708	Komisja Bioetyczna przy Okręgowej Izbie Lekarskiej w Łodzi, ul. Czerwona 3, Łódź 93-005, Poland	Prof. dr hab. n. prawnych Urszula Promińska	05-Oct-2022
Poland	5709	Komisja Bioetyczna przy Okręgowej Izbie Lekarskiej w Łodzi, ul. Czerwona 3, Łódź 93-005, Poland	Prof. dr hab. n. prawnych Urszula Promińska	05-Oct-2022
Poland	5710	Komisja Bioetyczna przy Okręgowej Izbie Lekarskiej w Łodzi, ul. Czerwona 3, Łódź 93-005, Poland	Prof. dr hab. n. prawnych Urszula Promińska	05-Oct-2022
Poland	5712	Komisja Bioetyczna przy Okręgowej Izbie Lekarskiej w Łodzi, ul. Czerwona 3, Łódź 93-005, Poland	Prof. dr hab. n. prawnych Urszula Promińska	05-Oct-2022
Poland	5713	Komisja Bioetyczna przy Okręgowej Izbie Lekarskiej w Łodzi, ul. Czerwona 3, Łódź 93-005, Poland	Prof. dr hab. n. prawnych Urszula Promińska	05-Oct-2022
Poland	5715	Komisja Bioetyczna przy Okręgowej Izbie Lekarskiej w Łodzi, ul. Czerwona 3, Łódź 93-005, Poland	Prof. dr hab. n. prawnych Urszula Promińska	05-Oct-2022
Poland	5716	Komisja Bioetyczna przy Okręgowej Izbie Lekarskiej w Łodzi, ul. Czerwona 3, Łódź 93-005, Poland	Prof. dr hab. n. prawnych Urszula Promińska	05-Oct-2022
Turkey	7601	Mersin University Clinical Research Ethics Committee Mersin University Ciftlikkoy Campus Prof. Dr. Ugur ORAL Cultural Center, 33343, Yenisehir/Mersin	Assoc.Professor Mustafa Azizoglu	21-Dec-2022
Turkey	7602	Mersin University Clinical Research Ethics Committee	Assoc.Professor Mustafa Azizoglu	21-Dec-2022

		Mersin University Ciftlikkoy Campus Prof. Dr. Ugur ORAL Cultural Center, 33343, Yenisehir/Mersin		
Turkey	7604	Mersin University Clinical Research Ethics Committee Mersin University Ciftlikkoy Campus Prof. Dr. Ugur ORAL Cultural Center, 33343, Yenisehir/Mersin	Assoc.Professor Mustafa Azizoglu	21-Dec-2022
Turkey	7605	Mersin University Clinical Research Ethics Committee Mersin University Ciftlikkoy Campus Prof. Dr. Ugur ORAL Cultural Center, 33343, Yenisehir/Mersin	Assoc.Professor Mustafa Azizoglu	21-Dec-2022
Turkey	7606	Mersin University Clinical Research Ethics Committee Mersin University Ciftlikkoy Campus Prof. Dr. Ugur ORAL Cultural Center, 33343, Yenisehir/Mersin	Assoc.Professor Mustafa Azizoglu	21-Dec-2022
United Kingdom	2801	Leicester Central Research Ethics Committee, Equinox House, City Link, Nottingham NG2 4LA, United Kingdom	Rita Patel	12-Sep-2022
United Kingdom	2802	Leicester Central Research Ethics Committee, Equinox House, City Link, Nottingham NG2 4LA, United Kingdom	Rita Patel	12-Sep-2022
United Kingdom	2803	Leicester Central Research Ethics Committee, Equinox House, City Link, Nottingham NG2 4LA, United Kingdom	Rita Patel	12-Sep-2022
United Kingdom	2804	Leicester Central Research Ethics Committee, Equinox House, City Link, Nottingham NG2 4LA, United Kingdom	Rita Patel	12-Sep-2022
United Kingdom	2805	Leicester Central Research Ethics Committee, Equinox House, City Link, Nottingham NG2 4LA, United Kingdom	Rita Patel	12-Sep-2022
United States of America	7801	WCG Institutional Review Board, 1019 39th Avenue SE Suite 120 Puyallup, Washington 98374-215, United States of America	Sharad Adekar	19-Sep-2022
United States of America	7802	WCG Institutional Review Board, 1019 39th Avenue SE Suite 120 Puyallup, Washington 98374-215, United States of America	Sharad Adekar	26-Oct-2022
United States of America	7803	WCG Institutional Review Board, 1019 39th Avenue SE Suite 120 Puyallup, Washington 98374-215, United States of America	Sharad Adekar	02-Sep-2022
United States of America	7805	WCG Institutional Review Board, 1019 39th Avenue SE Suite 120 Puyallup, Washington 98374-215, United States of America	Sharad Adekar	23-Sep-2022
United States of America	7805	WCG Institutional Review Board, 1019 39th Avenue SE Suite 120 Puyallup, Washington 98374-215, United States of America	Sharad Adekar	22-Sep-2022
United States of America	7806	WCG Institutional Review Board, 1019 39th Avenue SE Suite 120 Puyallup, Washington 98374-215, United States of America	Sharad Adekar	25-Oct-2022

United States of America	7807	WCG Institutional Review Board, 1019 39th Avenue SE Suite 120 Puyallup, Washington 98374-215, United States of America	Sharad Adekar	02-Sep-2022
United States of America	7808	WCG Institutional Review Board, 1019 39th Avenue SE Suite 120 Puyallup, Washington 98374-215, United States of America	Sharad Adekar	27-Sep-2022
United States of America	7809	WCG Institutional Review Board, 1019 39th Avenue SE Suite 120 Puyallup, Washington 98374-215, United States of America	Sharad Adekar	20-Sep-2022
United States of America	7810	WCG Institutional Review Board, 1019 39th Avenue SE Suite 120 Puyallup, Washington 98374-215, United States of America	Sharad Adekar	23-Sep-2022
United States of America	7812	WCG Institutional Review Board, 1019 39th Avenue SE Suite 120 Puyallup, Washington 98374-215, United States of America	Sharad Adekar	14-Sep-2022
United States of America	7813	WCG Institutional Review Board, 1019 39th Avenue SE Suite 120 Puyallup, Washington 98374-215, United States of America	Sharad Adekar	17-Sep-2022
United States of America	7814	WCG Institutional Review Board, 1019 39th Avenue SE Suite 120 Puyallup, Washington 98374-215, United States of America	Sharad Adekar	04-Sep-2022
United States of America	7815	WCG Institutional Review Board, 1019 39th Avenue SE Suite 120 Puyallup, Washington 98374-215, United States of America	Sharad Adekar	07-Oct-2022
United States of America	7816	WCG Institutional Review Board, 1019 39th Avenue SE Suite 120 Puyallup, Washington 98374-215, United States of America	Sharad Adekar	18-Sep-2022
United States of America	7817	WCG Institutional Review Board, 1019 39th Avenue SE Suite 120 Puyallup, Washington 98374-215, United States of America	Sharad Adekar	22-Sep-2022
United States of America	7818	WCG Institutional Review Board, 1019 39th Avenue SE Suite 120 Puyallup, Washington 98374-215, United States of America	Sharad Adekar	13-Sep-2022
United States of America	7819	WCG Institutional Review Board, 1019 39th Avenue SE Suite 120 Puyallup, Washington 98374-215, United States of America	Sharad Adekar	03-Nov-2022
United States of America	7820	WCG Institutional Review Board, 1019 39th Avenue SE Suite 120 Puyallup, Washington 98374-215, United States of America	Sharad Adekar	08-Sep-2022
United States of America	7821	WCG Institutional Review Board, 1019 39th Avenue SE Suite 120 Puyallup, Washington 98374-215, United States of America	Sharad Adekar	05-Oct-2022

IEC, Independent Ethics Committee; IRB, Institutional Review Board.

Supplementary Table S1: **Institutional Review Board/Independent Ethics Committee table**

	BGF HFO-1234ze 320/14·4/10 µg	BGF HFA-134a 320/14·4/10 µg
12-week safety analysis set	**N = 280** **n (%)**	**N = 278** **n (%)**
**Any SAE**	15 (5·4)	12 (4·3)
**Infections and infestations**	5 (1·8)	1 (0·4)
Bronchitis	1 (0·4)	0
COVID-19	0	1 (0·4)
COVID-19 pneumonia	1 (0·4)	0
Pneumonia	1 (0·4)	0
Pneumonia haemophilus	1 (0·4)	0
Pneumonia streptococcal	1 (0·4)	0
**Neoplasms benign, malignant, and unspecified (including cysts and polyps)**	0	2 (0·7)
Bladder neoplasm	0	1 (0·4)
Non-small cell lung cancer	0	1 (0·4)
**Cardiac disorders**	5 (1·8)	0
Acute myocardial infarction	2 (0·7)	0
Angina pectoris	1 (0·4)	0
Atrial fibrillation	1 (0·4)	0
Myocarditis	1 (0·4)	0
**Respiratory, thoracic, and mediastinal disorders**	2 (0·7)	5 (1·8)
COPD	2 (0·7)	5 (1·8)
**Injury, poisoning, and procedural complications**	1 (0·4)	2 (0·7)
Hip fracture	0	1 (0·4)
Sternal fracture	1 (0·4)	0
Thoracic vertebral fracture	1 (0·4)	0
Traumatic haemothorax	0	1 (0·4)
52-week safety analysis set	**N = 120** **n (%)**	**N = 120** **n (%)**
**Any SAE**	17 (14·2)	16 (13·3)
**Infections and infestations**	5 (4·2)	3 (2·5)
Atypical pneuomonia	1 (0·8)	0
Bronchitis	1 (0·8)	0
COVID-19	0	1 (0·8)
COVID-19 pneumonia	1 (0·8)	0
Pneumonia	1 (0·8)	1 (0·8)
Pneumonia haemophilus	1 (0·8)	0
Pneumonia pneumococcal	0	1 (0·8)
Sepsis	1 (0·8)	0
**Cardiac disorders**	2 (1·7)	3 (2·5)

Acute myocardial infarction	0	1 (0·8)
Arteriosclerosis coronary artery	0	1 (0·8)
Atrial fibrillation	1 (0·8)	0
Myocardial ischaemia	0	1 (0·8)
Myocarditis	1 (0·8)	0
**Respiratory thoracic and mediastinal disorders**	5 (4·2)	9 (7·5)
Acute respiratory failure	1 (0·8)	0
COPD	5 (4·2)	7 (5·8)
Haemothorax	0	1 (0·8)
Pulmonary embolism	0	1 (0·8)
**Gastrointestinal disorders**	2 (1·7)	0
Gastric varices	1 (0·8)	0
Gastritis	1 (0·8)	0
**Musculoskeletal and connective tissue disorders**	2 (1·7)	1 (0·8)
Fibromyalgia	0	1 (0·8)
Osteoarthritis	2 (1·7)	0
Vertebral lateral recess stenosis	1 (0·8)	0
**Injury, poisoning, and procedural complications**	0	2 (1·7)
Hip fracture	0	1 (0·8)
Thoracic vertebral fracture	0	1 (0·8)

[a]Adverse events (AEs) with an onset date on or after the date of the first dose of investigational product (IP) throughout the treatment period up to and including the date of the last IP dose + 1 day.

[b]Participants with multiple occurrences are counted once regardless of the number of occurrences.

AE, adverse event; BGF, budesonide/glycopyrronium/formoterol fumarate dihydrate; COPD, chronic obstructive pulmonary disease; COVID-19, Coronavirus Disease 2019; HFA-134a, hydrofluoroalkane-134a; HFO-1234ze, hydrofluoroolefin-1234ze; IP, investigational product; MedDRA, Medical Dictionary for Regulatory Activities; N, total number of participants in treatment group; n, number of participants with AE; SAE, serious adverse events.

Supplementary Table S2: **Participants with any serious adverse events (SAE) and SAEs by System Organ Class and Preferred Term (Medical Dictionary for Regulatory Activities [MedDRA] version 26·1) across 12 and 52 weeks[a,b] (reported in ≥2 participants in any treatment group by System Organ Class).**

	BGF HFO-1234ze 320/14·4/10 µg	BGF HFA-134a 320/14·4/10 µg
12-week safety analysis set	N = 280<br>n (%)	N = 278<br>n (%)
**Any AE leading to IP discontinuation**	20 (7·1)	9 (3·2)
**Infections and infestations**	4 (1·4)	0
COVID-19	1 (0·4)	0
Oral fungal infection	1 (0·4)	0
Oropharyngeal candidiasis	1 (0·4)	0
Pneumonia haemophilus	1 (0·4)	0
**Immune system disorders**	1 (0·4)	0
Drug hypersensitivity	1 (0·4)	0
**Nervous system disorders**	1 (0·4)	0
Dizziness	1 (0·4)	0
**Cardiac disorders**	6 (2·1)	1 (0·4)
Acute myocardial infarction	2 (0·7)	0
Angina pectoris	1 (0·4)	0
Ischaemic cardiomyopathy	0	1 (0·4)
Myocarditis	1 (0·4)	0
Tachycardia	1 (0·4)	0
Ventricular tachycardia	1 (0·4)	0
**Respiratory, thoracic, and mediastinal disorders**	7 (2·5)	6 (2·2)
COPD	2 (0·7)	4 (1·4)
Cough	1 (0·4)	0
Dysphonia	1 (0·4)	0
Dyspnoea	1 (0·4)	2 (0·7)
Pulmonary mass	1 (0·4)	0
Increased sputum	1 (0·4)	0
**General disorders and administration site conditions**	1 (0·4)	0
Performance status decreased	1 (0·4)	0
**Injury, poisoning, and procedural complications**	0	2 (0·7)
Hip fracture	0	1 (0·4)
Traumatic haemothorax	0	1 (0·4)
52-week safety analysis set	N = 120<br>n (%)	N = 120<br>n (%)
**Any AE leading to IP discontinuation**	11 (9·2)	9 (7·5)
**Infections and infestations**	3 (2·5)	0
Oral fungal infection	1 (0·8)	0
Oropharyngeal candidiasis	1 (0·8)	0

Pneumonia haemophilus	1 (0·8)	0
**Neoplasms benign, malignant, and unspecified (including cysts and polyps)**	0	1 (0·8)
Tongue neoplasm malignant stage unspecified	0	1 (0·8)
**Nervous system disorders**	1 (0·8)	0
Dizziness	1 (0·8)	0
**Cardiac disorders**	2 (1·7)	1 (0·8)
Angina unstable[c]	0	1 (0·8)
Myocarditis	1 (0·8)	0
Ventricular tachycardia	1 (0·8)	0
**Respiratory, thoracic, and mediastinal disorders**	4 (3·3)	6 (5·0)
COPD	2 (1·7)	3 (2·5)
Cough	1 (0·8)	1 (0·8)
Dyspnoea	0	1 (0·8)
Haemothorax	0	1 (0·8)
Increased sputum	1 (0·8)	0
**Musculoskeletal and connective tissue disorders**	0	1 (0·8)
Mobility decreased	0	1 (0·8)
**General disorders and administration site conditions**	1 (0·8)	0
Performance status decreased	1 (0·8)	0

[a]Adverse events (AEs) with an onset date on or after the date of the first dose of investigational product (IP) throughout the treatment period up to and including the date of the last IP dose + 1 day.

[b]Participants with multiple occurrences are counted once regardless of the number of occurrences.

[c]One additional participant receiving hydrofluoroalkane-134a (HFA-134a) discontinued due to an AE after completing 12 weeks of treatment. Since this participant died on the same day as the occurrence of the discontinuation due to an AE, they are categorised as having discontinued due to death as the last available reason.

AE, adverse event; BGF, budesonide/glycopyrronium/formoterol fumarate dihydrate; COPD, chronic obstructive pulmonary disease; COVID-19, Coronavirus Disease 2019; HFA-134a, hydrofluoroalkane-134a; HFO-1234ze, hydrofluoroolefin-1234ze; IP, investigational product; MedDRA, Medical Dictionary for Regulatory Activities; N, total number of participants in treatment group; n, number of participants with AE.

Supplementary Table S3: **Participants with any discontinuation due to an adverse event (AE) and discontinuations due to an AE by System Organ Class and Preferred Term (Medical Dictionary for Regulatory Activities [MedDRA] version 26·1) across 12 and 52 weeks.[a,b]**

	BGF HFO-1234ze 320/14·4/10 µg	BGF HFA-134a 320/14·4/10 µg
12-week safety analysis set	**N = 280** **n (%)**	**N = 278** **n (%)**
COPD	39 (13·9)	40 (14·4)
Dysphonia	11 (3·9)	8 (2·9)
Cough	5 (1·8)	3 (1·1)
Dyspnoea	4 (1·4)	3 (1·1)
Increased sputum	2 (0·7)	2 (0·7)
Allergic cough	1 (0·4)	0
Bronchospasm	1 (0·4)	0
Dyspnoea exertional	1 (0·4)	0
Productive cough	1 (0·4)	0
Throat irritation	0	1 (0·4)
52-week safety analysis set	**N = 120** **n (%)**	**N = 120** **n (%)**
COPD	34 (28·3)	43 (35·8)
Dysphonia	5 (4·2)	3 (2·5)
Cough	3 (2·5)	4 (3·3)
Dyspnoea	2 (1·7)	2 (1·7)
Increased sputum	2 (1·7)	2 (1·7)

[a]Adverse events (AEs) with an onset date on or after the date of the first dose of investigational product (IP) throughout the treatment period up to and including the date of the last IP dose + 1 day.

[b]Participants with multiple occurrences are counted once regardless of the number of occurrences.

AE, adverse event; BGF, budesonide/glycopyrronium/formoterol fumarate dihydrate; COPD, chronic obstructive pulmonary disease; HFA-134a, hydrofluoroalkane-134a; HFO-1234ze, hydrofluoroolefin-1234ze; IP, investigational product; MedDRA, Medical Dictionary for Regulatory Activities; N, total number of participants in treatment group; n, number of participants with AE.

Supplementary Table S4: **All adverse events (AEs) of special interest by Preferred Term (Medical Dictionary for Regulatory Activities [MedDRA] version 26·1) across 12 and 52 weeks.[a,b]**

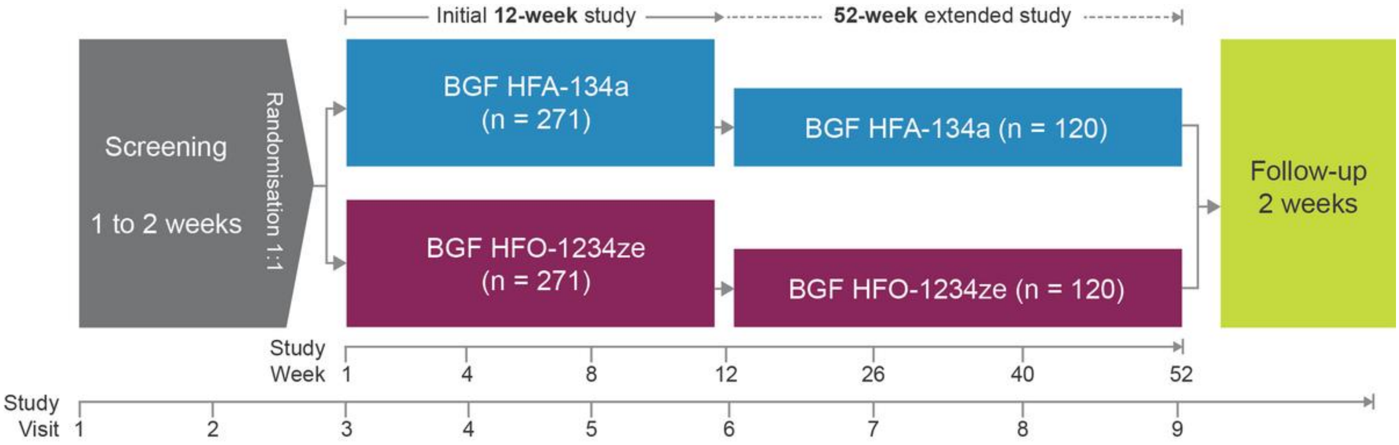

Supplementary Fig. S1: **Study design.** Note that telephone contact was required every 2 weeks outside of clinic visits following the start of study treatment. BGF, budesonide/glycopyrronium/formoterol fumarate dihydrate; HFA-134a, hydrofluoroalkane-134a; HFO-1234ze, hydrofluoroolefin-1234ze.

References

1 Celli BR, MacNee W, ATS/ERS Task Force. Standards for the diagnosis and treatment of patients with COPD: a summary of the ATS/ERS position paper. *Eur Respir J* 2004; **23**: 932–46.
